# Supplementary material for: Design and Synthesis of a Novel 4-aryl-N-(2-alkoxythieno [2,3-b]pyrazine-3-yl)-4-arylpiperazine-1-carboxamide DGG200064 Showed Therapeutic Effect on Colon Cancer through G2/M Arrest
Source: Pharmaceuticals (Basel). 2022 Apr 20;15(5):502. doi: 10.3390/ph15050502 (PMC9143821; doi:10.3390/ph15050502)
Supplement: Supplementary file 1 [file pharmaceuticals-15-00502-s001.zip › pharmaceuticals-1657853-supplementary.pdf]

Supplementary Materials

Contents

1. ^{1}H NMR, ^{13}C NMR, LC/MS, and HRMS Spectra of Compounds........2

2. Figures S1 and S2.........40

3. Raw Biological Data........41

Table S1. *In vivo* evaluation of the DGG200066 suppressed colon cancer tumor growth against HCT1116 cells.........56

1. ^{1}H NMR, ^{13}C NMR, LC/MS, and HRMS Spectra of Compounds

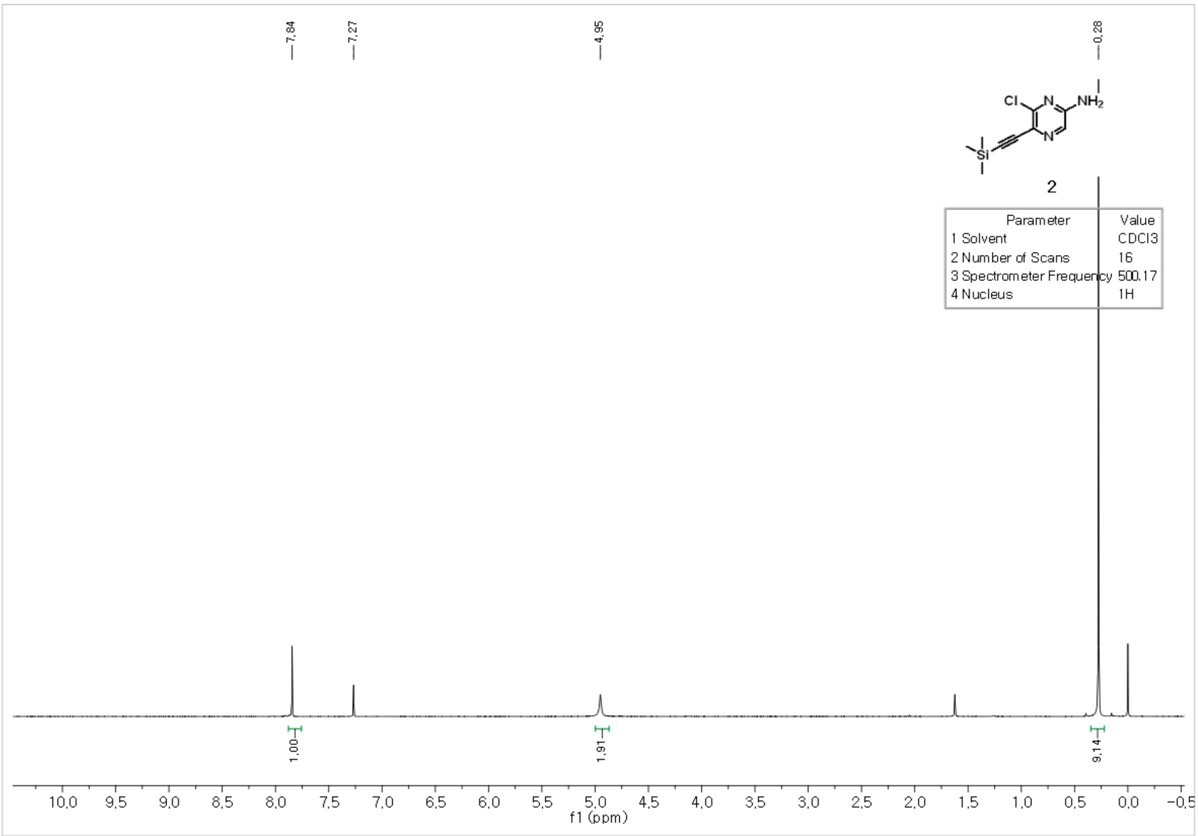

^{1}H-NMR-2

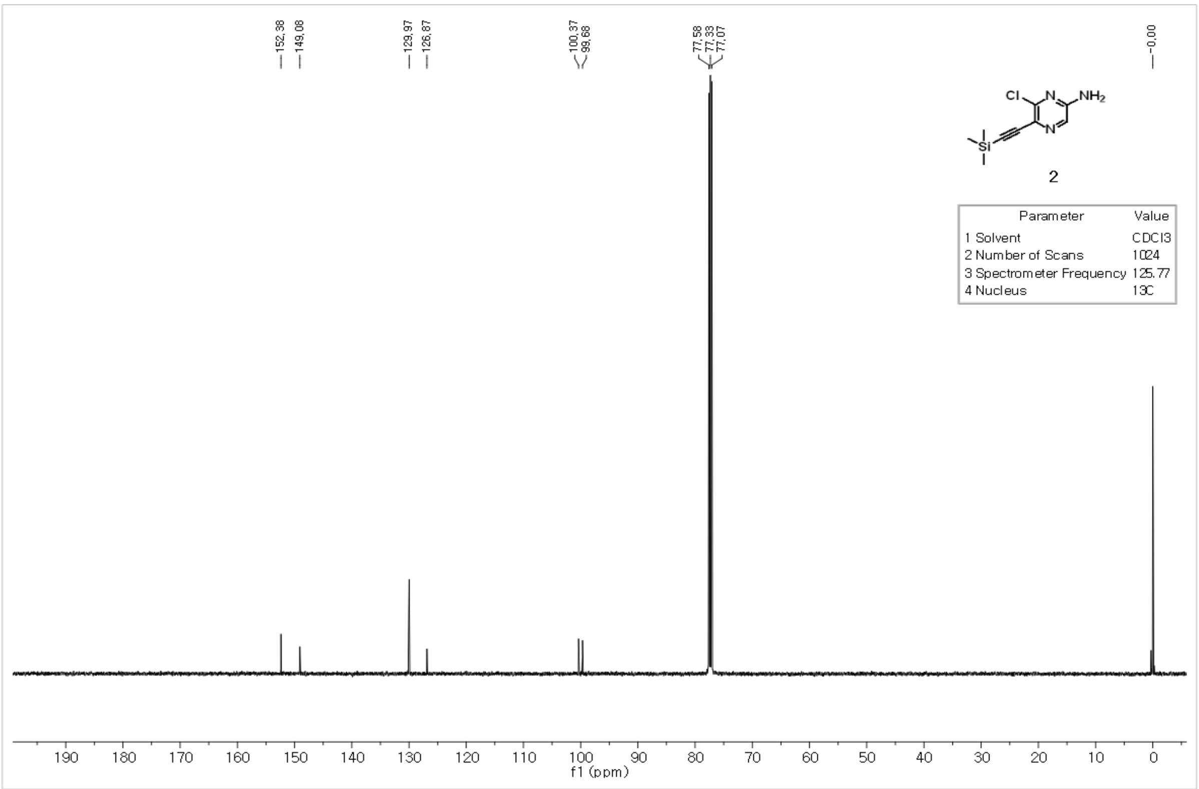

^{13}C-NMR-2

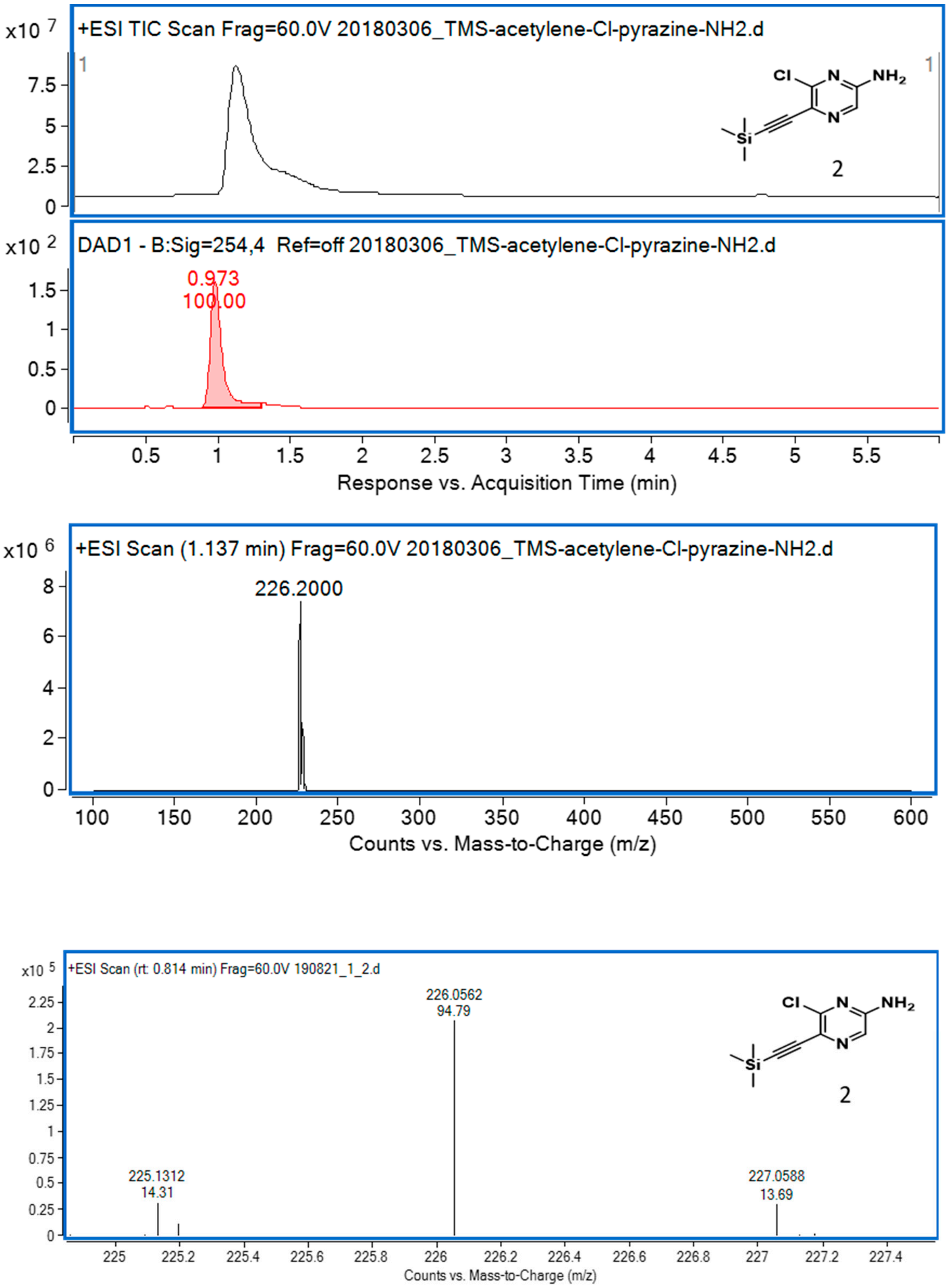

x10 7
+ESI TIC Scan Frag=60.0V 20180306_TMS-acetylene-Cl-pyrazine-NH2.d
Cl
N
NH2
N
Si
2
x10 2
DAD1 - B:Sig=254,4 Ref=off 20180306_TMS-acetylene-Cl-pyrazine-NH2.d
0.973
100.00
Response vs. Acquisition Time (min)
x10 6
+ESI Scan (1.137 min) Frag=60.0V 20180306_TMS-acetylene-Cl-pyrazine-NH2.d
226.2000
Counts vs. Mass-to-Charge (m/z)
x10 5
+ESI Scan (rt: 0.814 min) Frag=60.0V 190821_1_2.d
226.0562
94.79
225.1312
14.31
227.0588
13.69
Cl
N
NH2
N
Si
2
Counts vs. Mass-to-Charge (m/z)

LC/MS, HR/MS-2

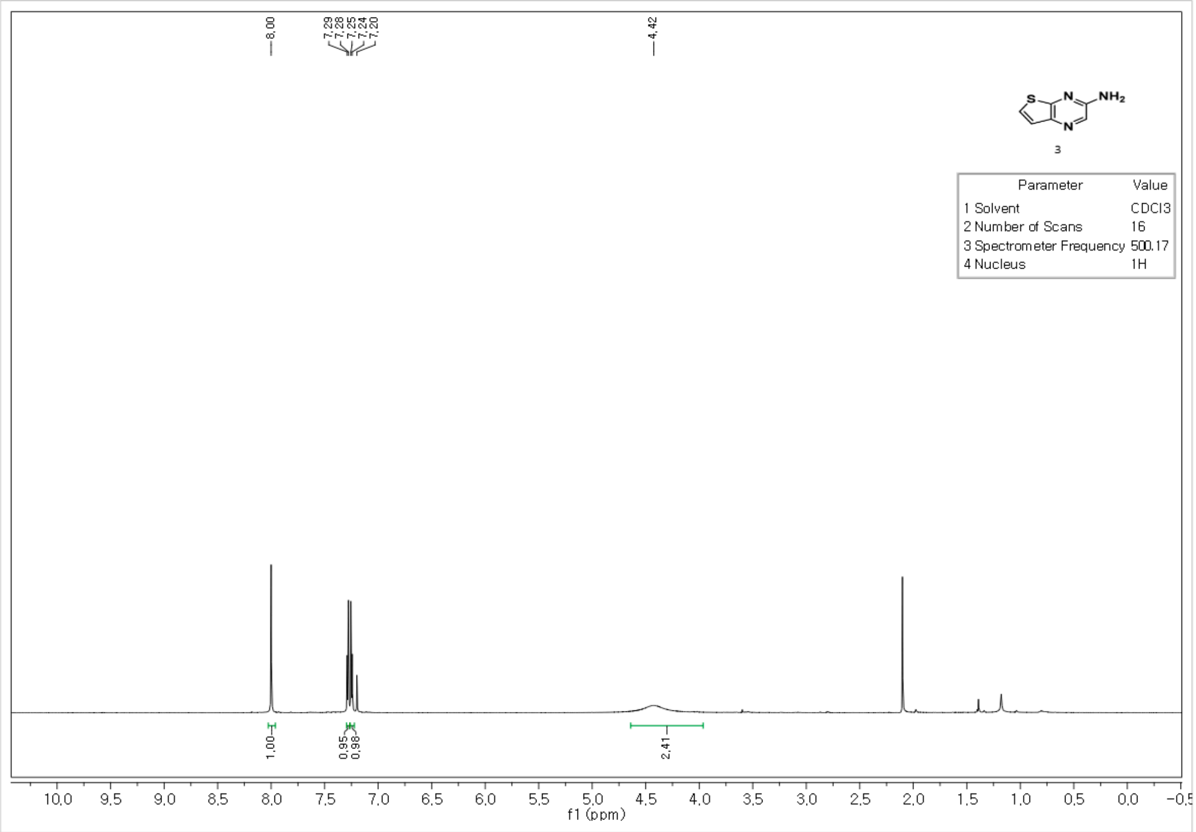

^{1}H-NMR-3

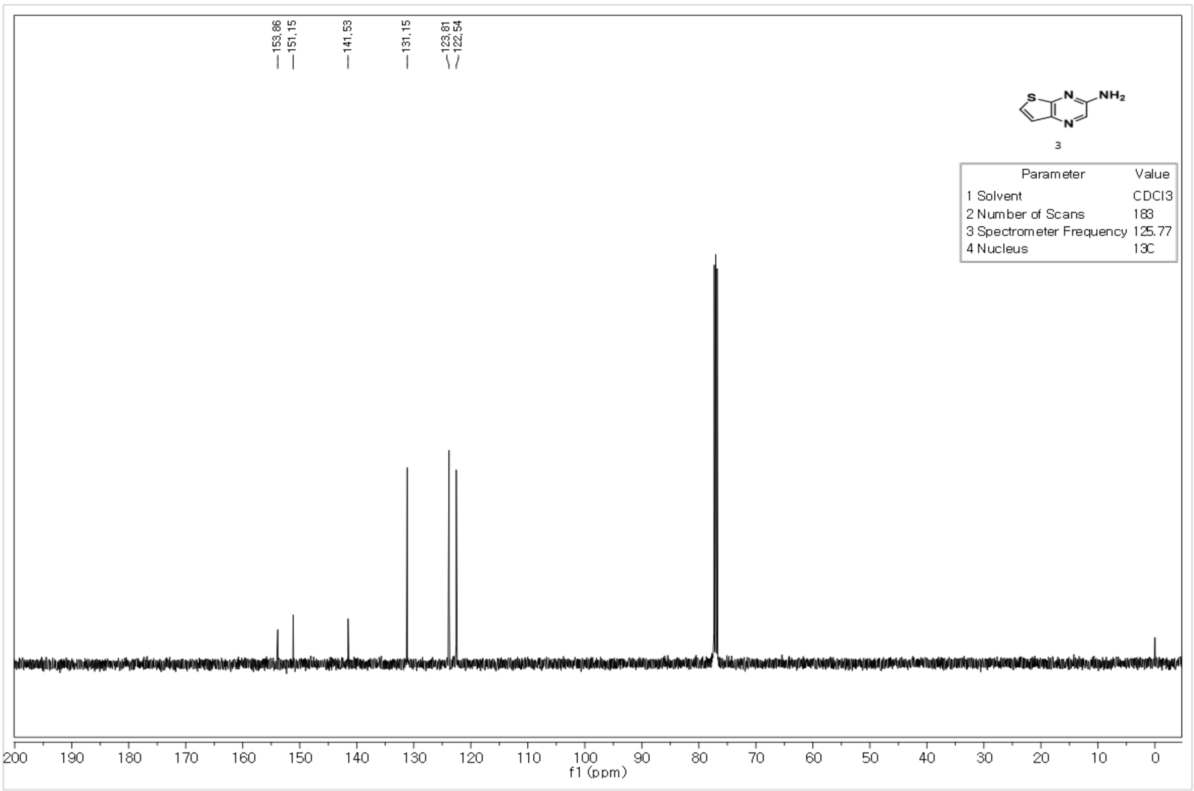

^{13}C-NMR-3

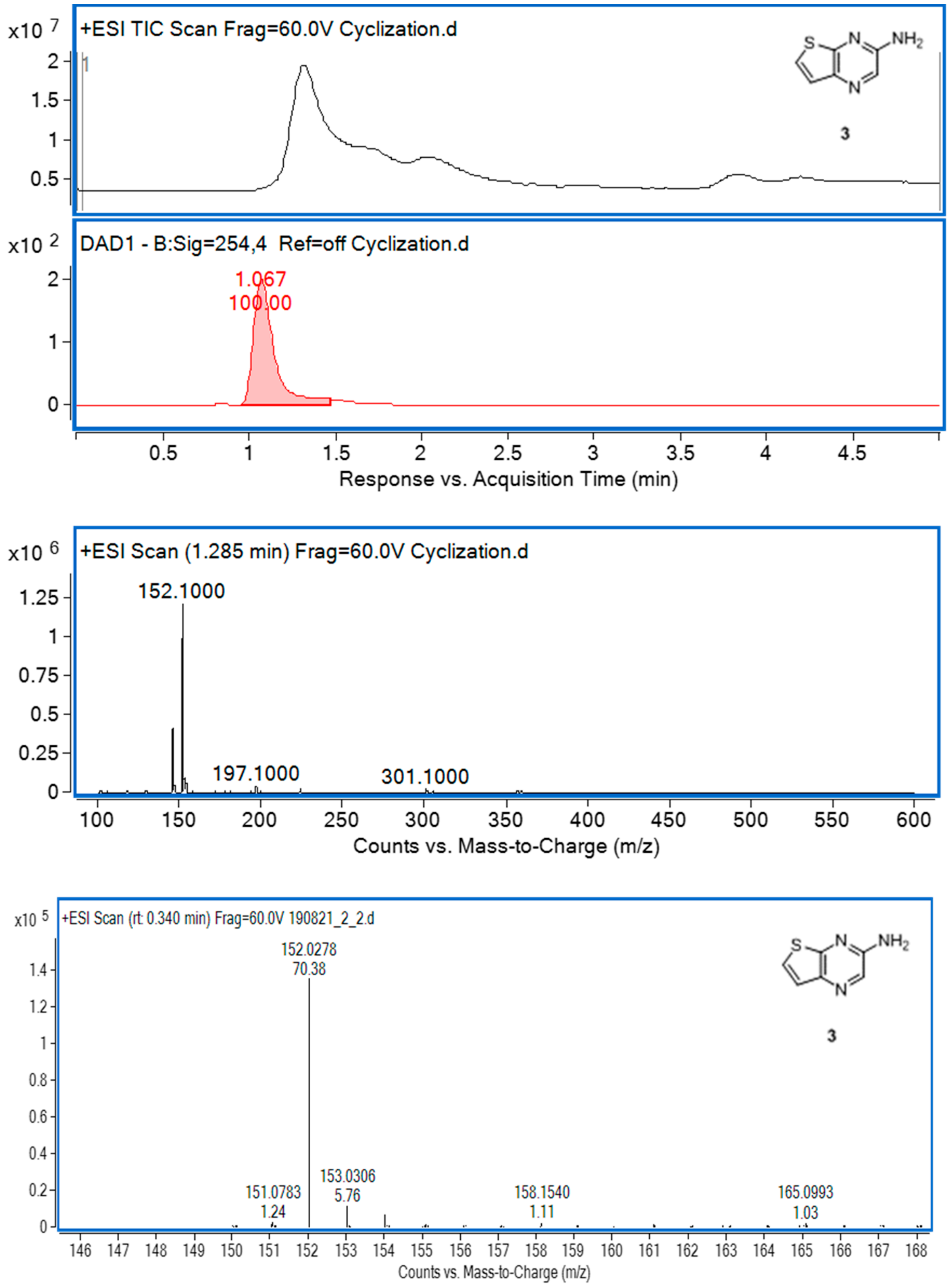
x10 7
+ESI TIC Scan Frag=60.0V Cyclization.d
NH2
S
N
N
3
x10 2
DAD1 - B:Sig=254,4 Ref=off Cyclization.d
1.067
100.00
Response vs. Acquisition Time (min)
x10 6
+ESI Scan (1.285 min) Frag=60.0V Cyclization.d
152.1000
197.1000
301.1000
Counts vs. Mass-to-Charge (m/z)
x10 5
+ESI Scan (rt: 0.340 min) Frag=60.0V 190821_2_2.d
152.0278
70.38
151.0783
1.24
153.0306
5.76
158.1540
1.11
165.0993
1.03
Counts vs. Mass-to-Charge (m/z)

LC/MS, HR/MS-3

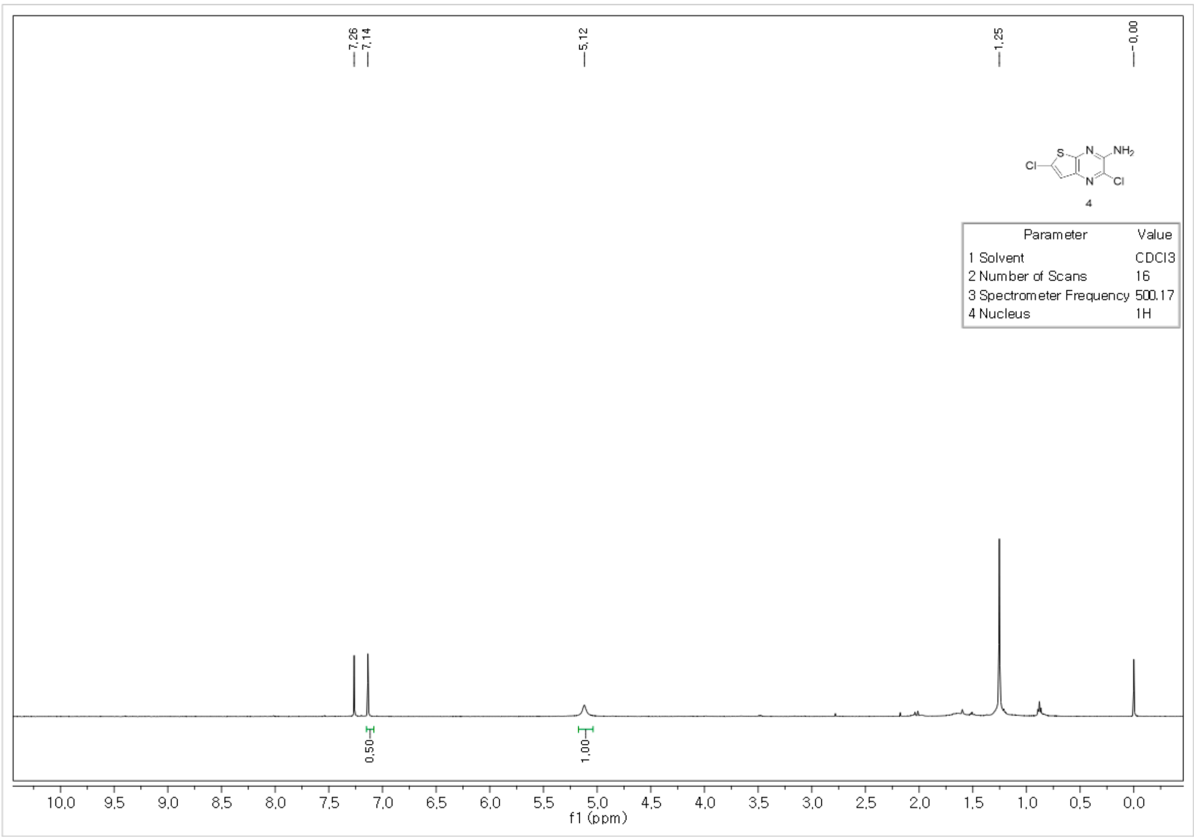

^{1}H-NMR-4

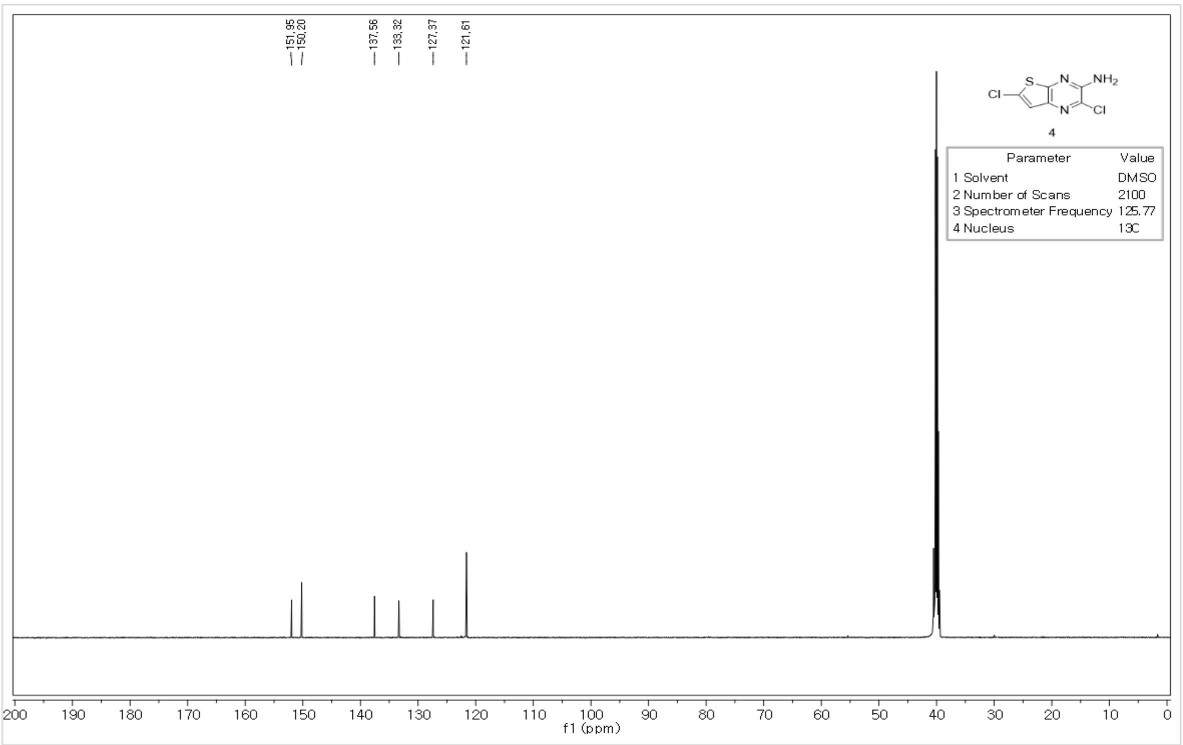

^{13}C-NMR-4

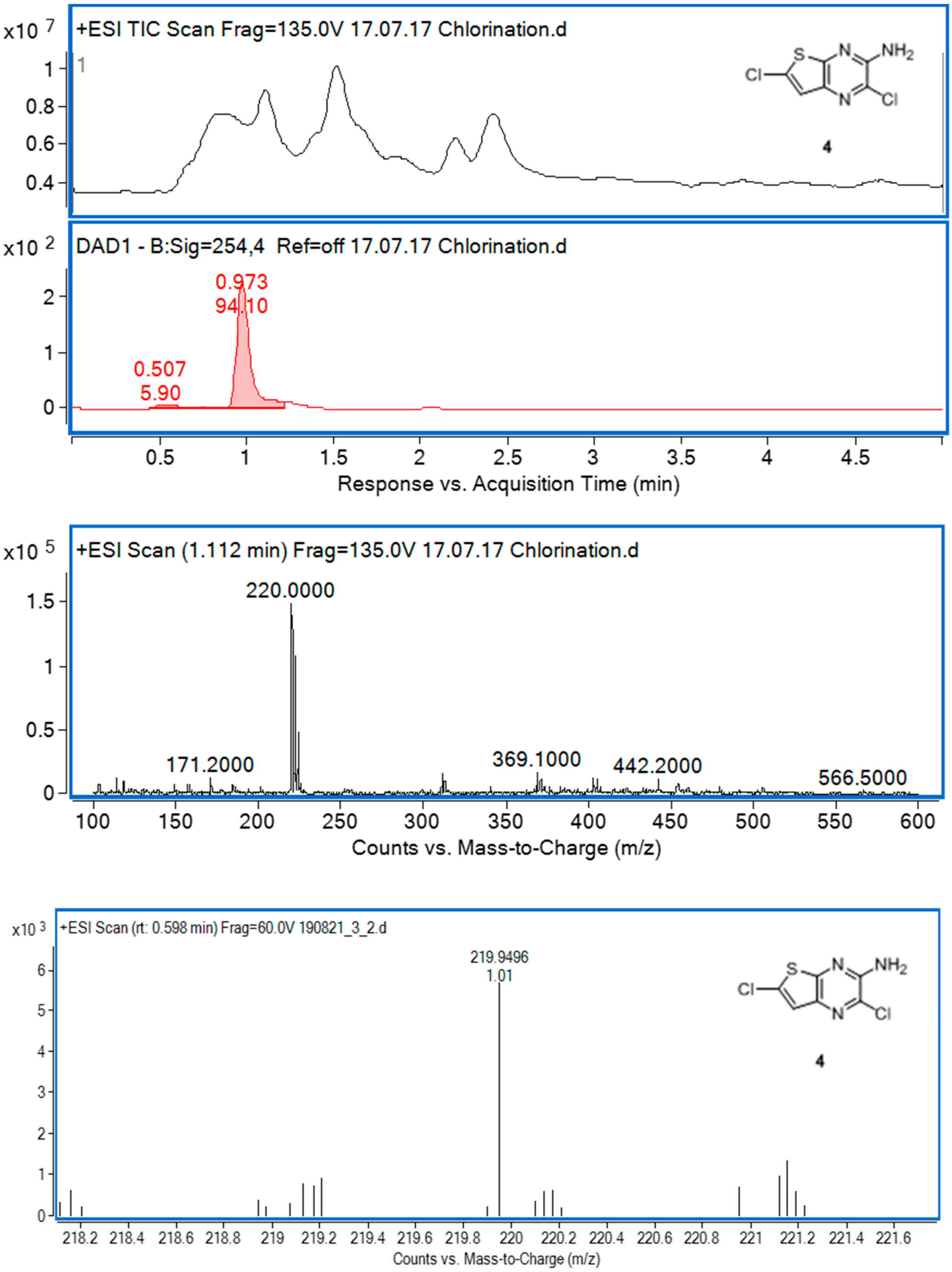
+ESI TIC Scan Frag=135.0V 17.07.17 Chlorination.d
x10 7
DAD1 - B:Sig=254,4 Ref=off 17.07.17 Chlorination.d
x10 2
0.973
94.10
0.507
5.90
Response vs. Acquisition Time (min)
+ESI Scan (1.112 min) Frag=135.0V 17.07.17 Chlorination.d
x10 5
220.0000
171.2000
369.1000
442.2000
566.5000
Counts vs. Mass-to-Charge (m/z)
+ESI Scan (rt: 0.598 min) Frag=60.0V 190821_3_2.d
x10 3
219.9496
1.01
Counts vs. Mass-to-Charge (m/z)
Cl
S
N
NH_2
4

LC/MS, HR/MS-4

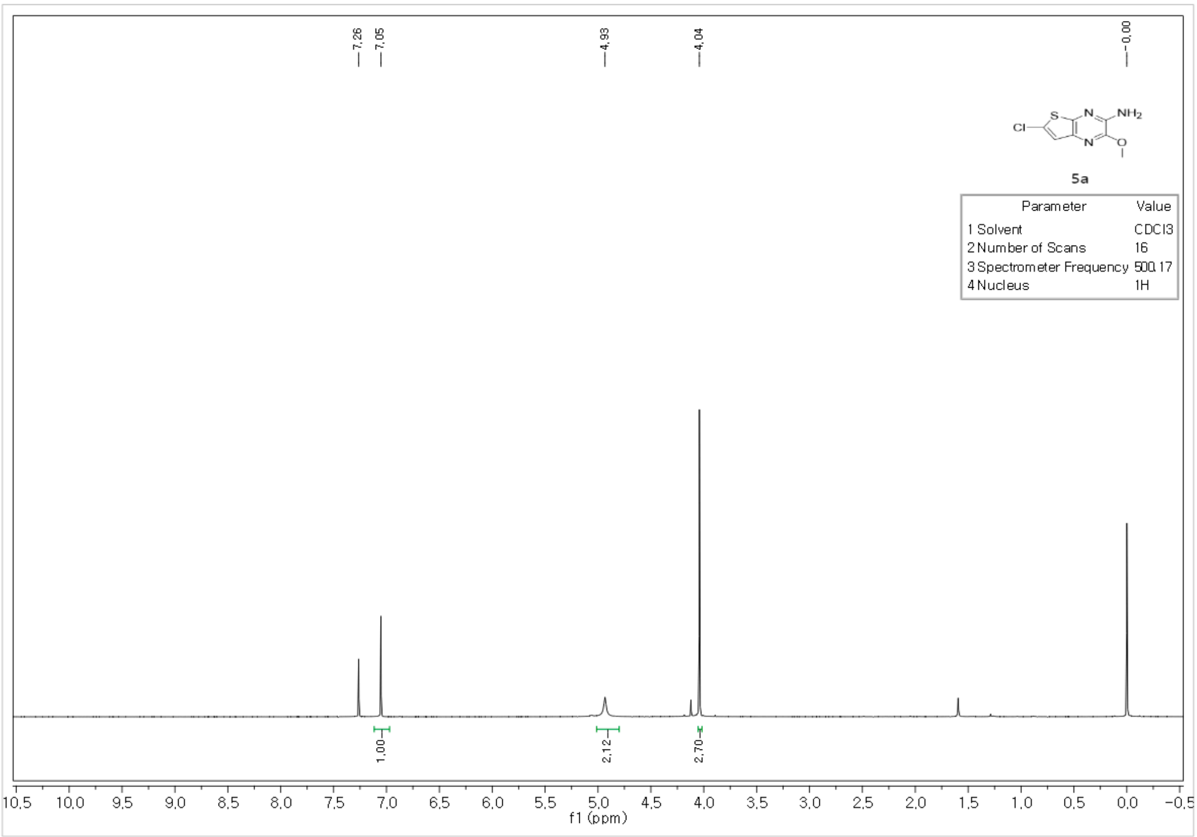

^{1}H-NMR-5a

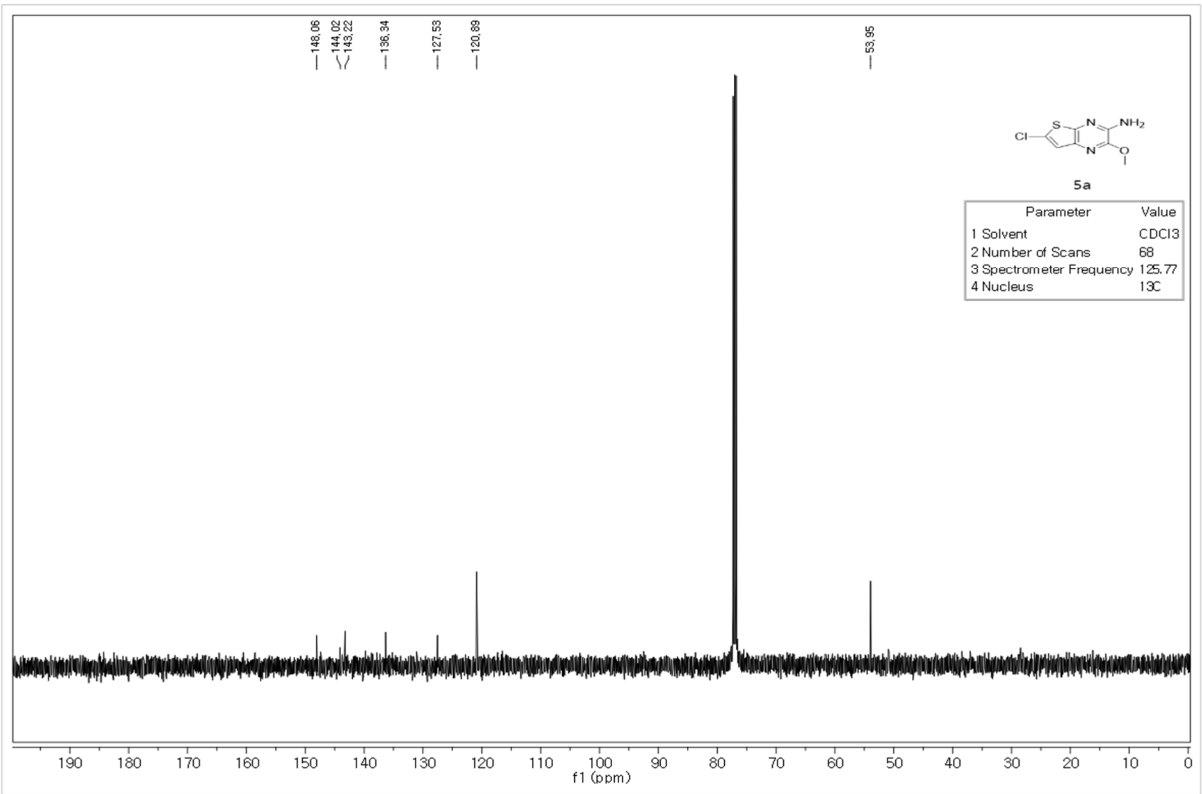

^{13}C-NMR-5a

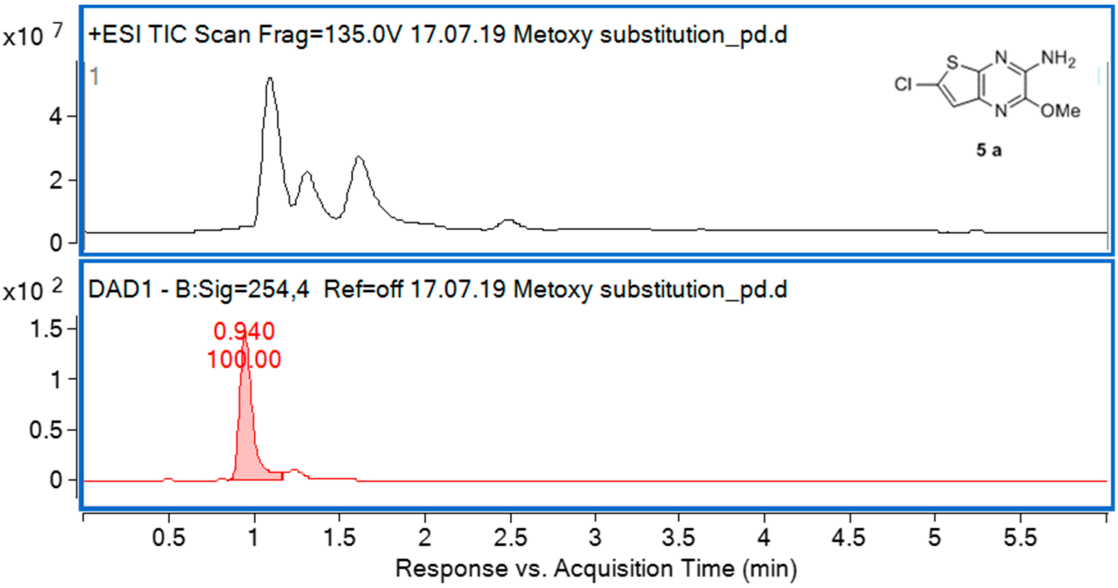
x10 7
+ESI TIC Scan Frag=135.0V 17.07.19 Metoxy substitution_pd.d
5 a
x10 2
DAD1 - B:Sig=254,4 Ref=off 17.07.19 Metoxy substitution_pd.d
0.940
100.00
Response vs. Acquisition Time (min)

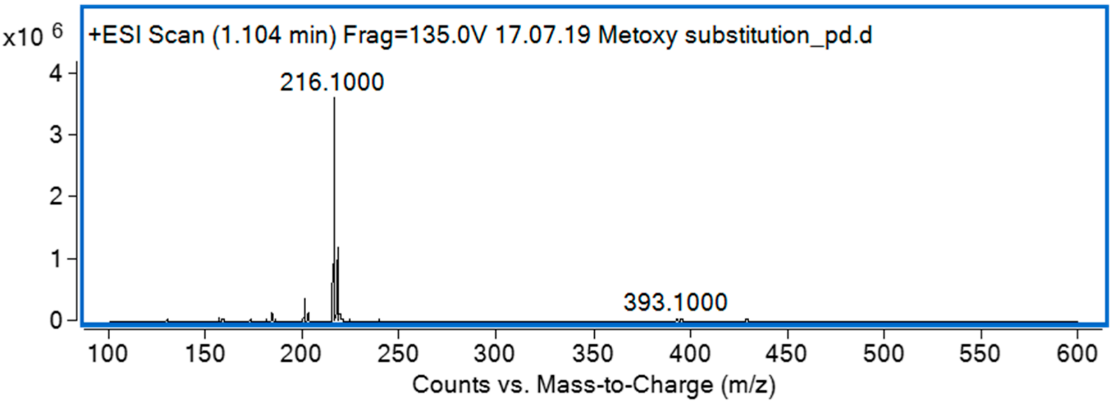
x10 6
+ESI Scan (1.104 min) Frag=135.0V 17.07.19 Metoxy substitution_pd.d
216.1000
393.1000
Counts vs. Mass-to-Charge (m/z)

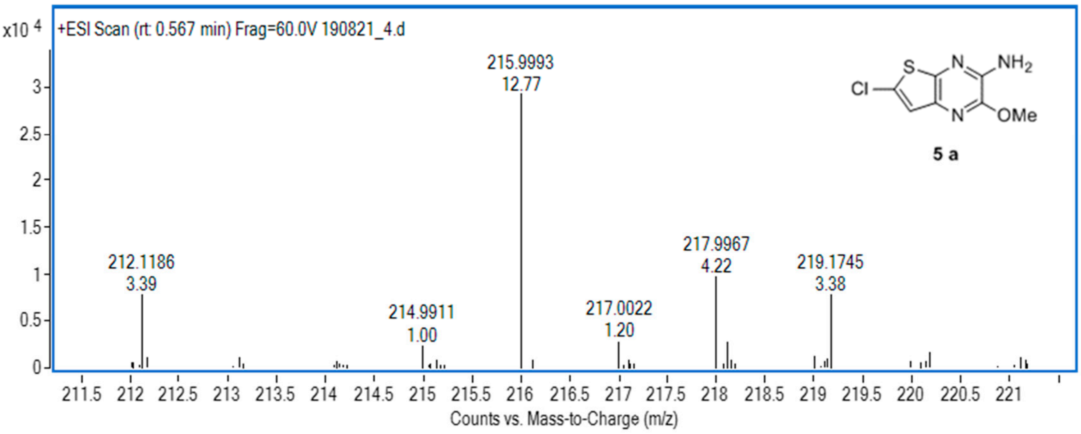
x10 4
+ESI Scan (rt: 0.567 min) Frag=60.0V 190821_4.d
215.9993
12.77
212.1186
3.39
214.9911
1.00
217.0022
1.20
217.9967
4.22
219.1745
3.38
5 a
Counts vs. Mass-to-Charge (m/z)

LC/MS, HR/MS-5a

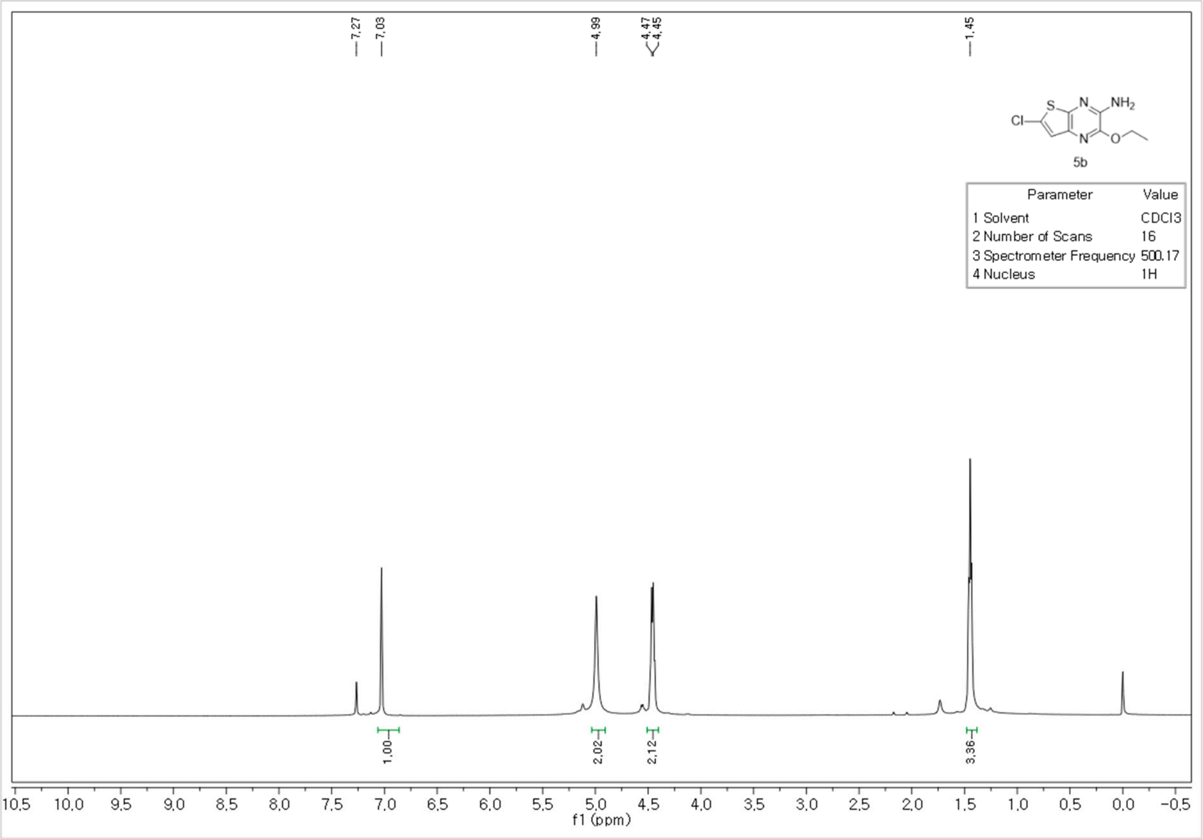

^{1}H-NMR-5b

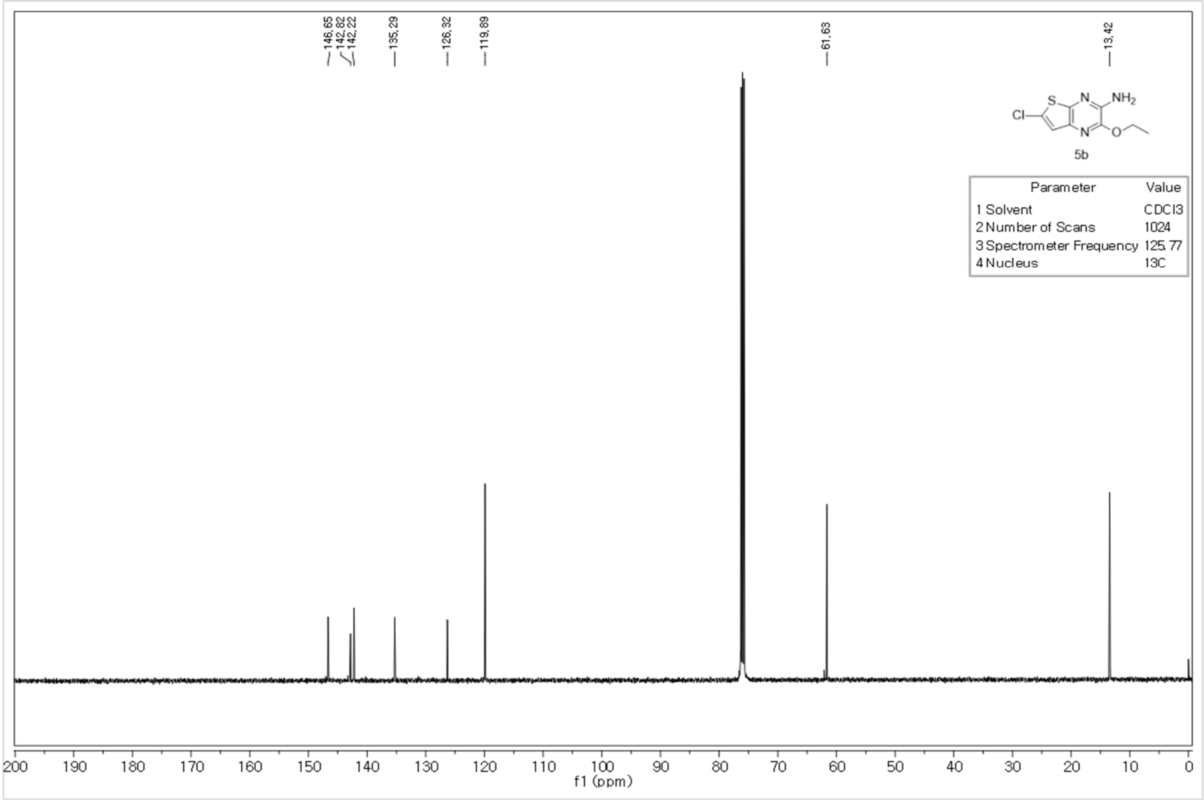

^{13}C-NMR-5b

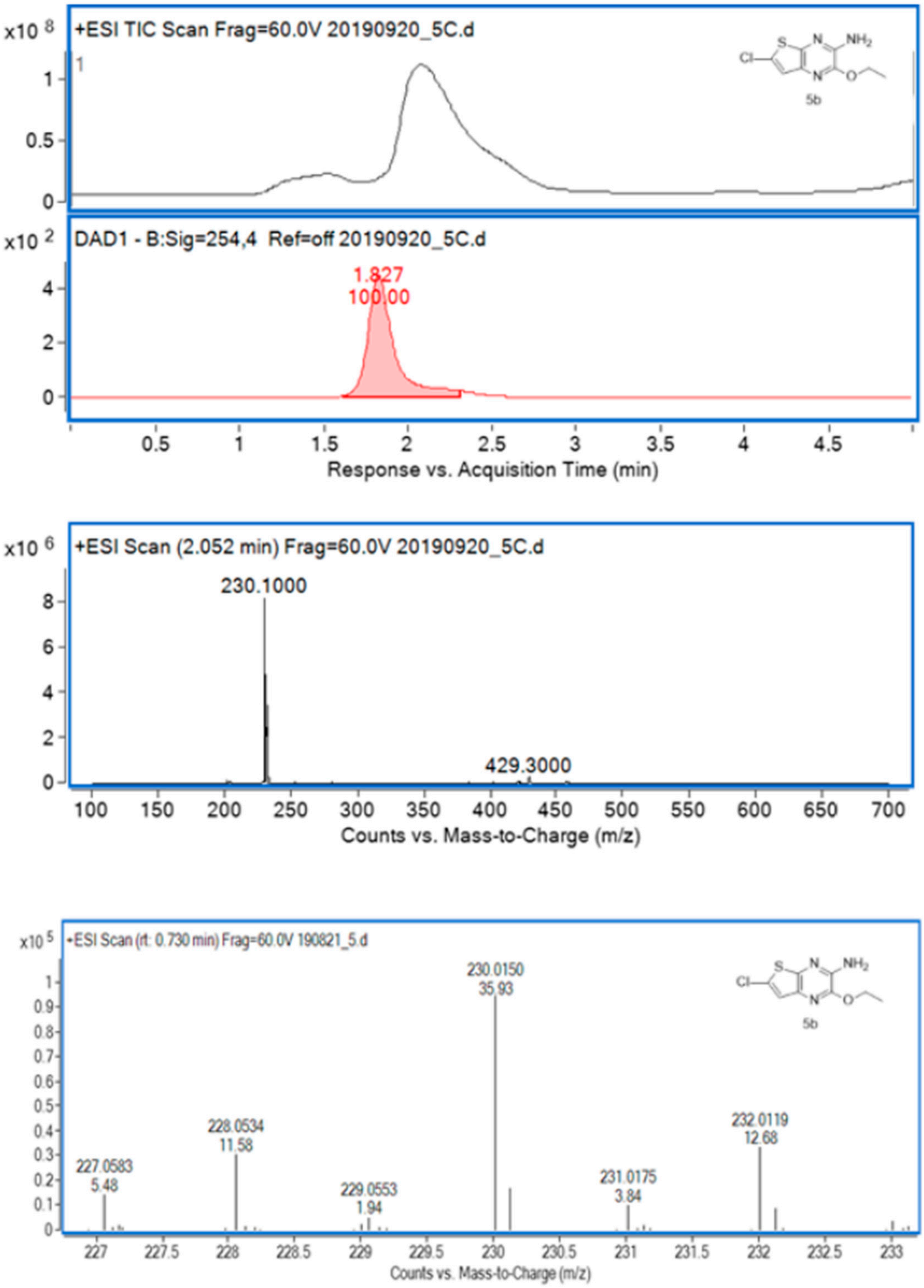
x10 8
+ESI TIC Scan Frag=60.0V 20190920_5C.d
5b
x10 2
DAD1 - B:Sig=254,4 Ref=off 20190920_5C.d
1.827
100.00
Response vs. Acquisition Time (min)
x10 6
+ESI Scan (2.052 min) Frag=60.0V 20190920_5C.d
230.1000
429.3000
Counts vs. Mass-to-Charge (m/z)
x10 5
+ESI Scan (rt: 0.730 min) Frag=60.0V 190821_5.d
230.0150
35.93
228.0534
11.58
227.0583
5.48
229.0553
1.94
231.0175
3.84
232.0119
12.68
5b
Counts vs. Mass-to-Charge (m/z)

LC/MS, HR/MS-5b

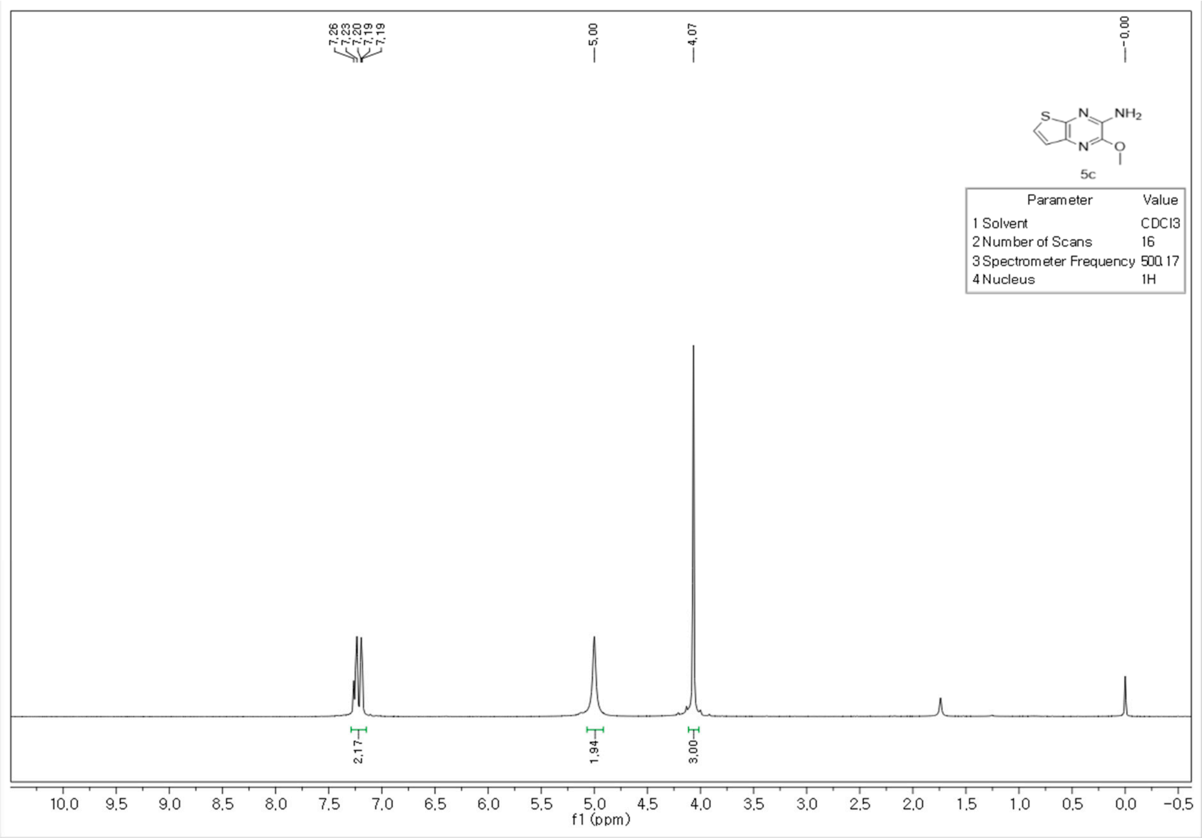

^{1}H-NMR-5c

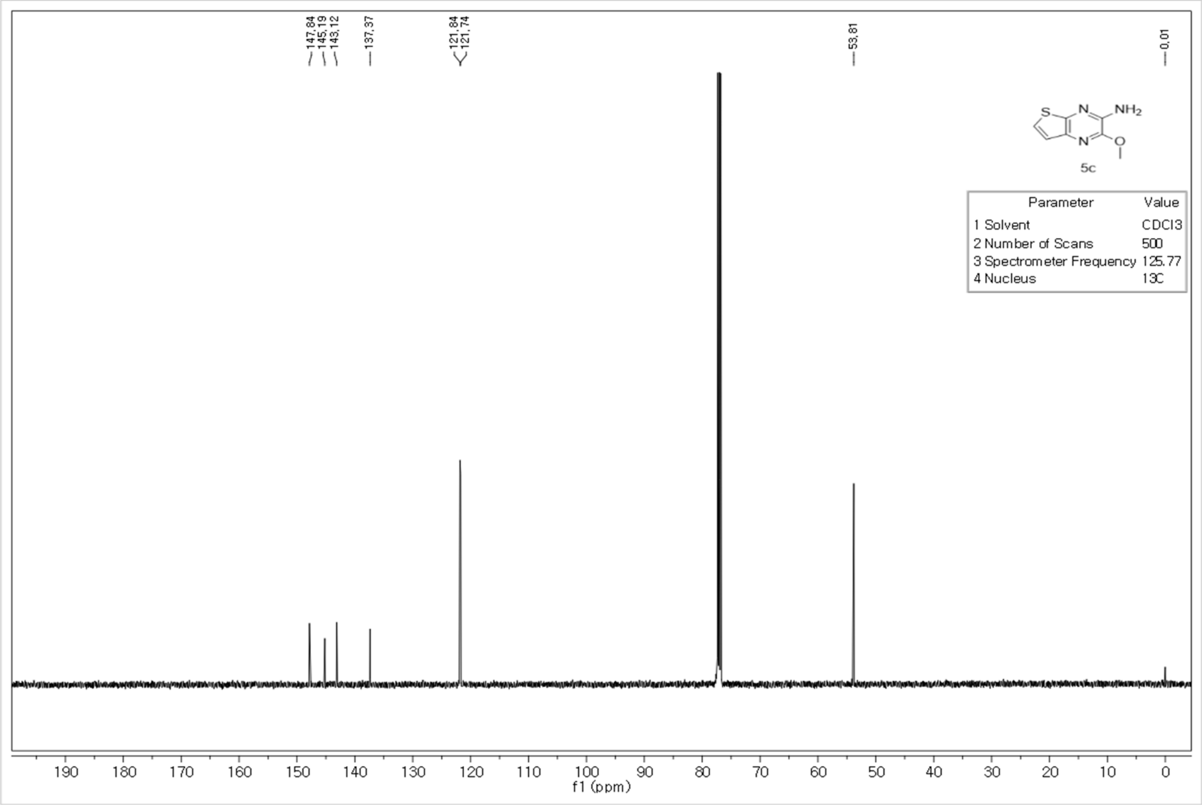

^{13}C-NMR-5c

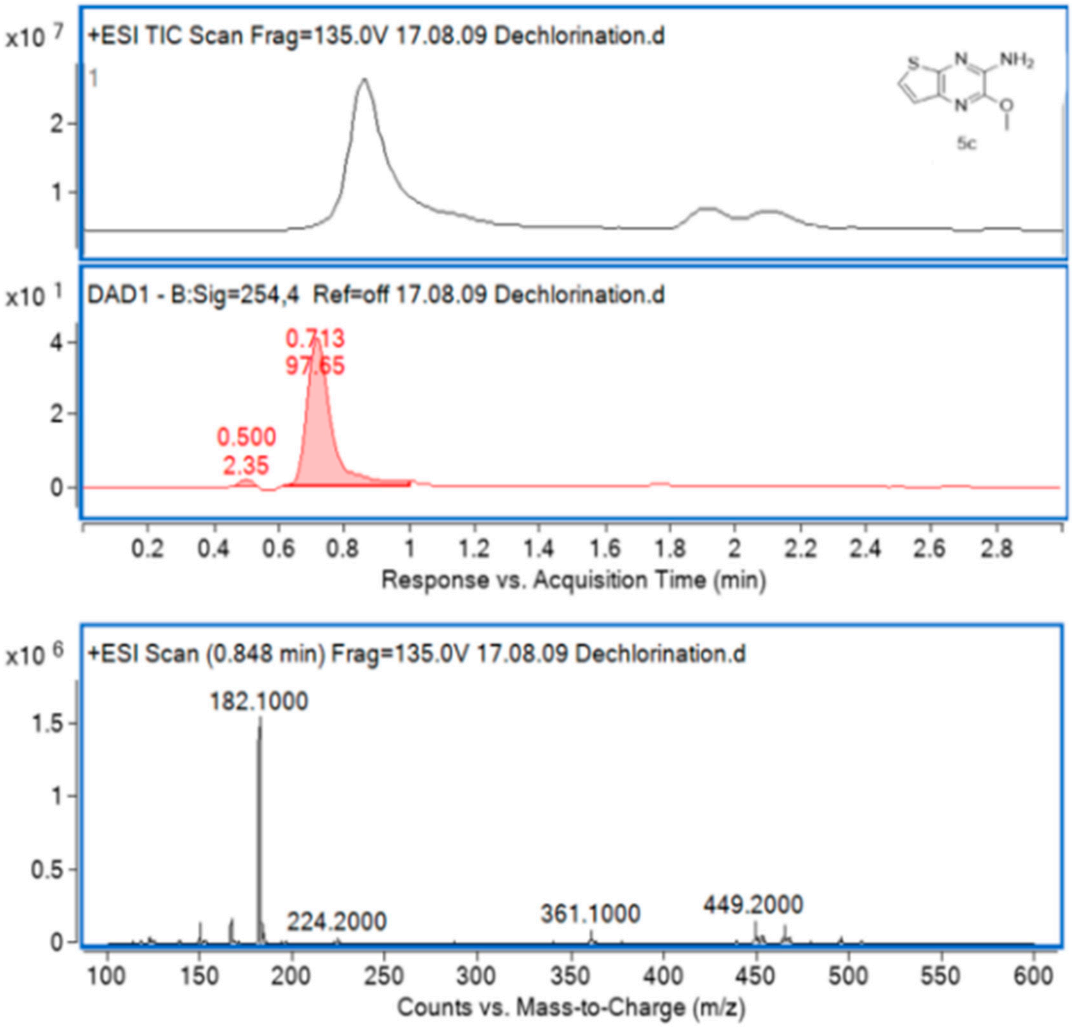
+ESI TIC Scan Frag=135.0V 17.08.09 Dechlorination.d
DAD1 - B:Sig=254,4 Ref=off 17.08.09 Dechlorination.d
0.713
97.65
0.500
2.35
Response vs. Acquisition Time (min)
+ESI Scan (0.848 min) Frag=135.0V 17.08.09 Dechlorination.d
182.1000
224.2000
361.1000
449.2000
Counts vs. Mass-to-Charge (m/z)
5c

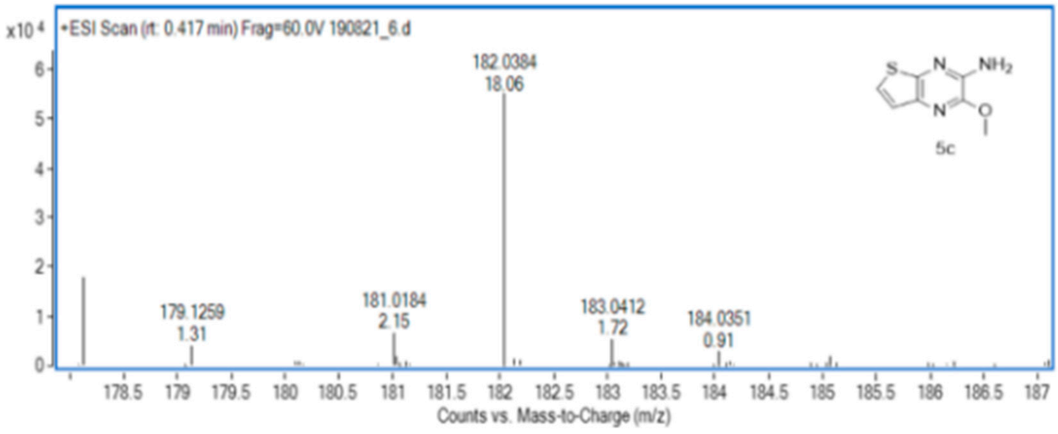
+ESI Scan (rt: 0.417 min) Frag=60.0V 190821_6.d
182.0384
18.06
179.1259
1.31
181.0184
2.15
183.0412
1.72
184.0351
0.91
Counts vs. Mass-to-Charge (m/z)
5c

LC/MS, HR/MS-5c

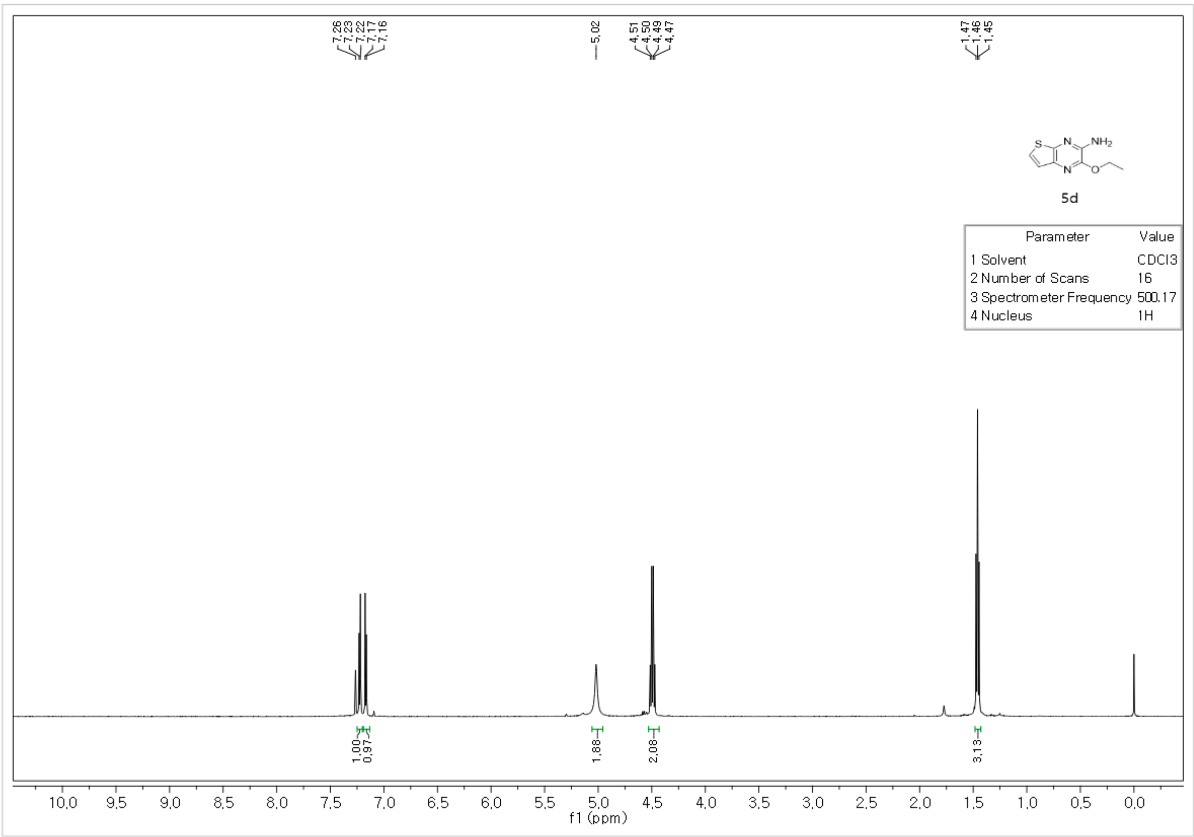

^{1}H-NMR-5d

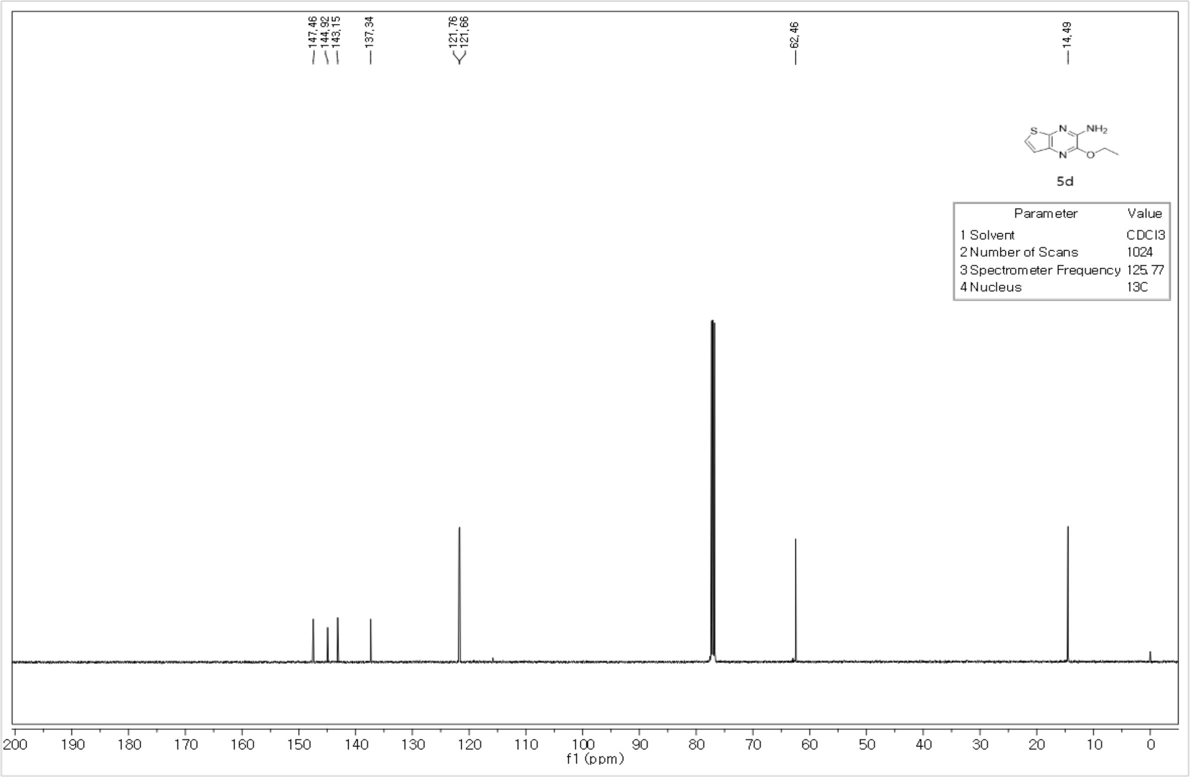

^{13}C-NMR-5d

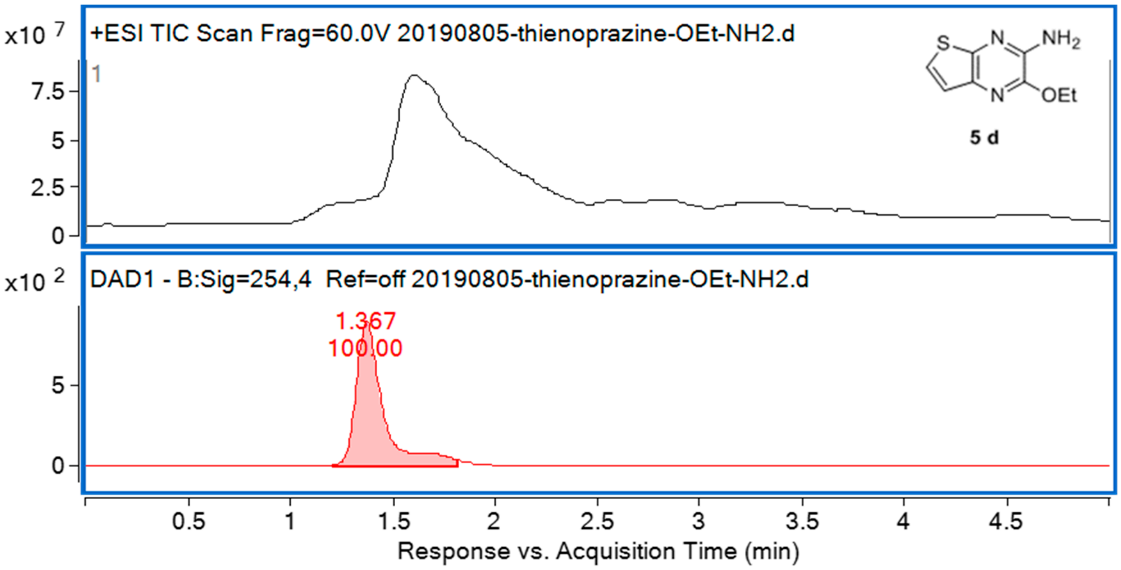

+ESI TIC Scan Frag=60.0V 20190805-thienoprazine-OEt-NH2.d
x10 7
5 d
DAD1 - B:Sig=254,4 Ref=off 20190805-thienoprazine-OEt-NH2.d
x10 2
1.367
100.00
Response vs. Acquisition Time (min)

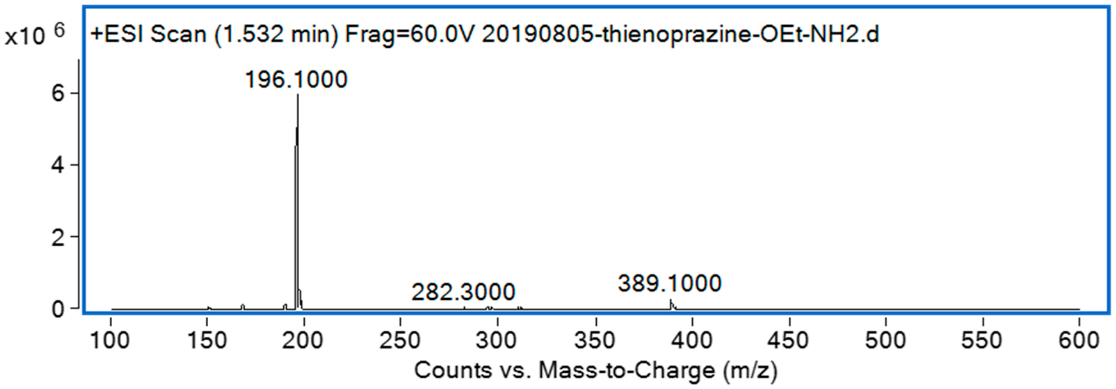

+ESI Scan (1.532 min) Frag=60.0V 20190805-thienoprazine-OEt-NH2.d
x10 6
196.1000
282.3000
389.1000
Counts vs. Mass-to-Charge (m/z)

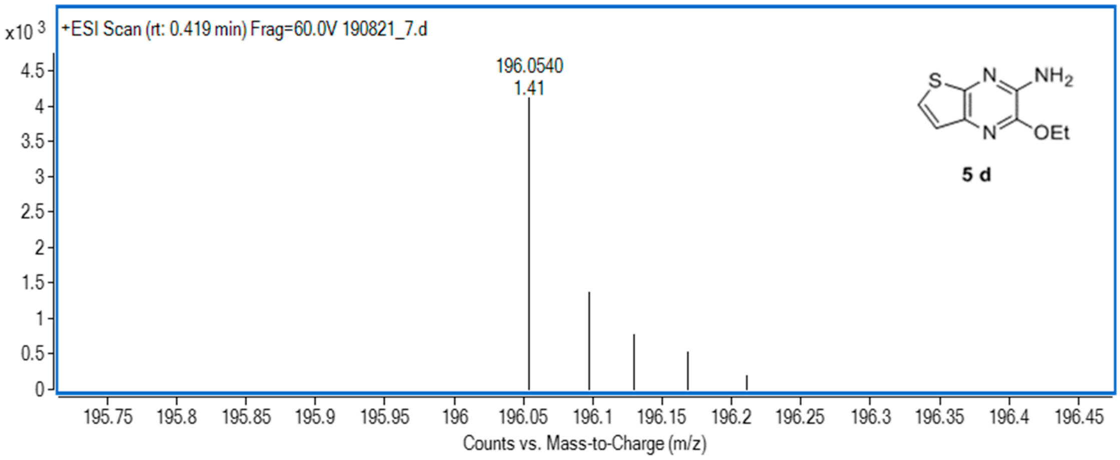

+ESI Scan (rt: 0.419 min) Frag=60.0V 190821_7.d
x10 3
196.0540
1.41
5 d
Counts vs. Mass-to-Charge (m/z)

LC/MS, HR/MS-5d

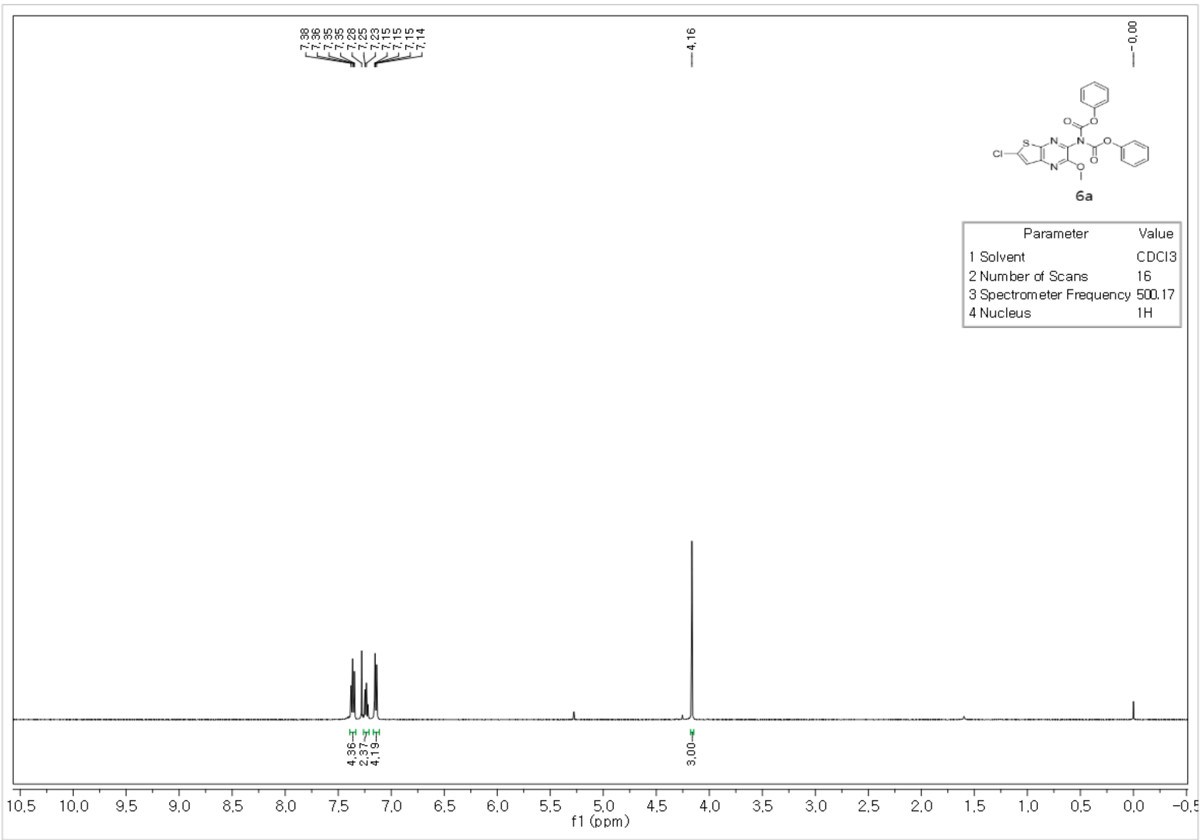

^{1}H-NMR-6a

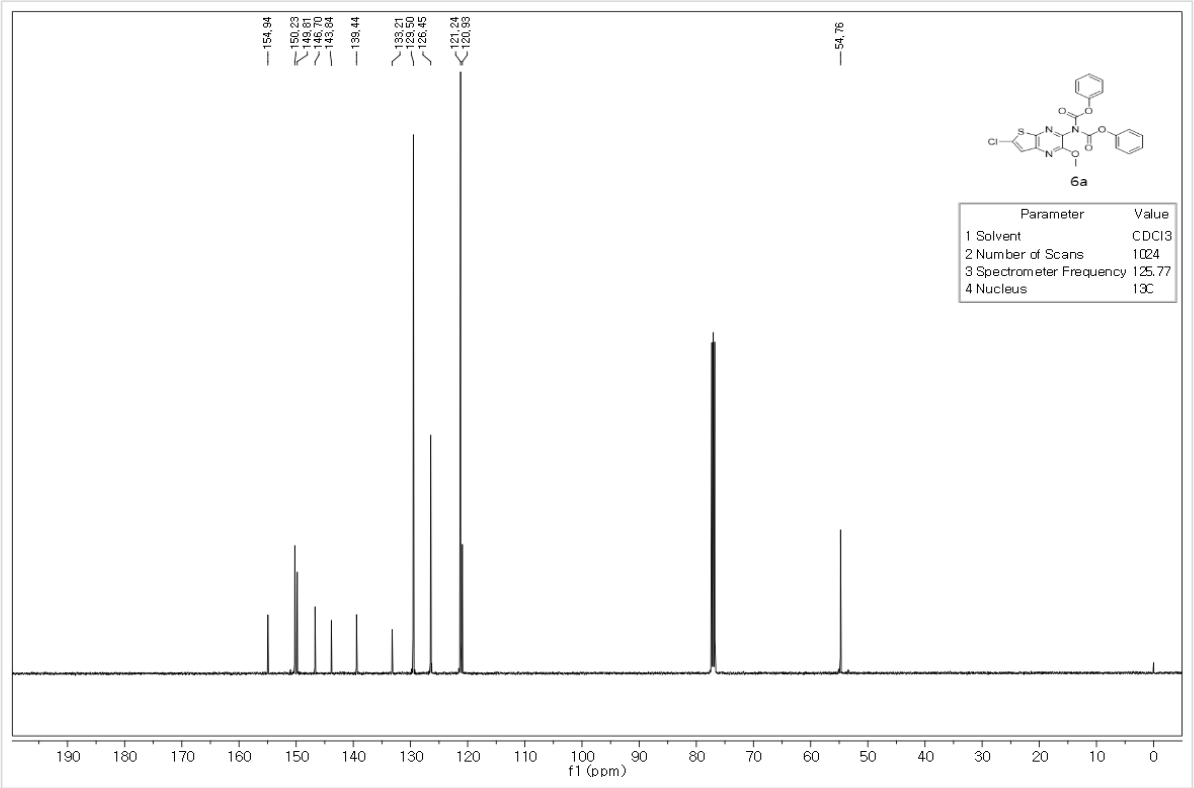

^{13}C-NMR-6a

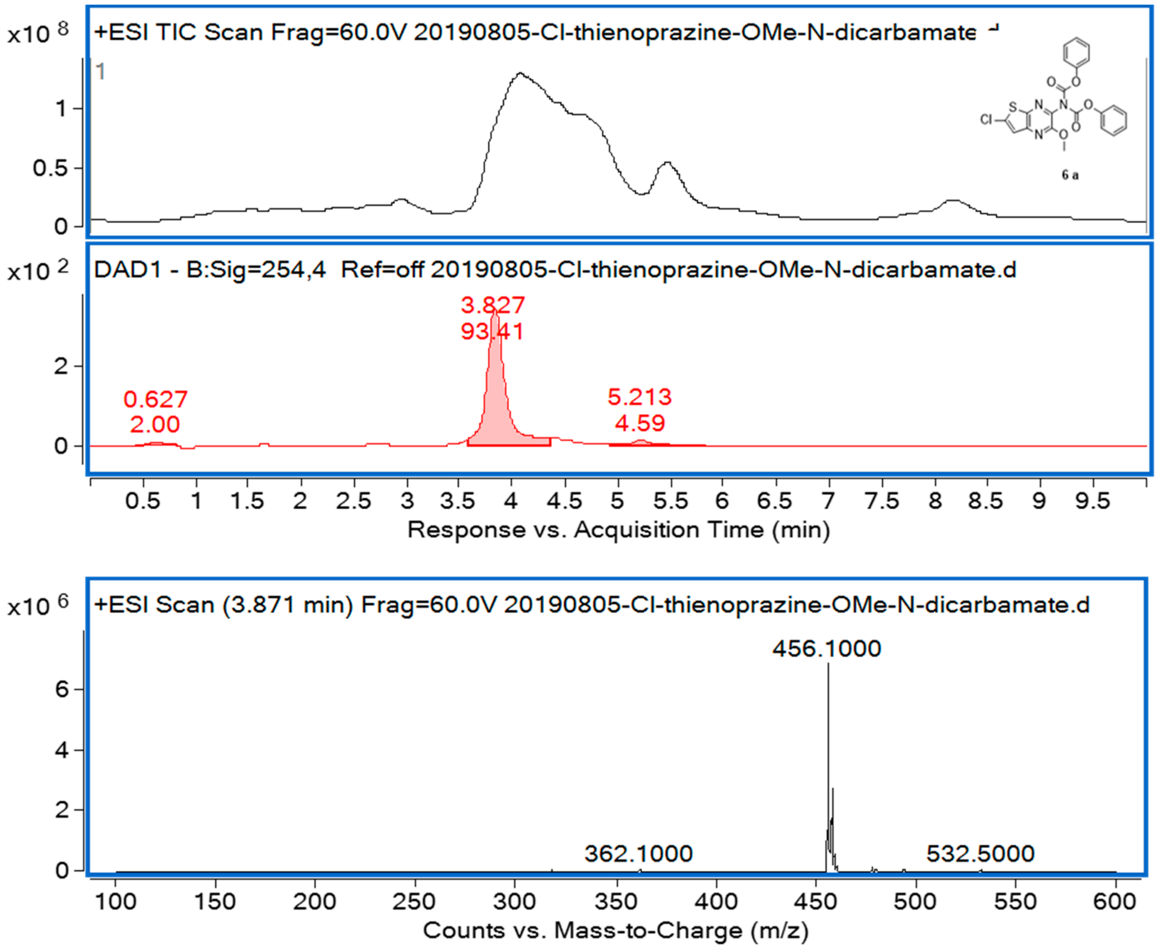

x10 8
+ESI TIC Scan Frag=60.0V 20190805-Cl-thienoprazine-OMe-N-dicarbamate
6 a
x10 2
DAD1 - B:Sig=254,4 Ref=off 20190805-Cl-thienoprazine-OMe-N-dicarbamate.d
3.827
93.41
0.627
2.00
5.213
4.59
Response vs. Acquisition Time (min)
x10 6
+ESI Scan (3.871 min) Frag=60.0V 20190805-Cl-thienoprazine-OMe-N-dicarbamate.d
456.1000
362.1000
532.5000
Counts vs. Mass-to-Charge (m/z)

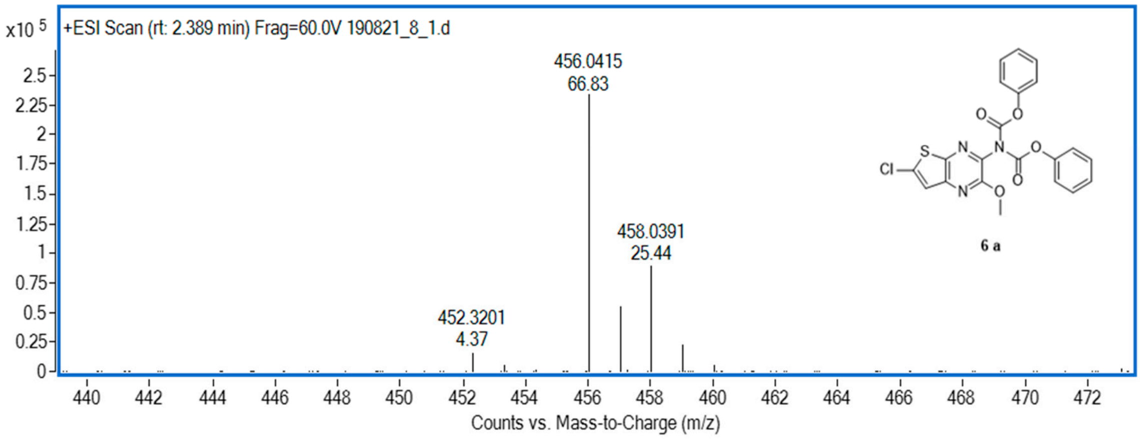

x10 5
+ESI Scan (rt: 2.389 min) Frag=60.0V 190821_8_1.d
456.0415
66.83
458.0391
25.44
452.3201
4.37
6 a
Counts vs. Mass-to-Charge (m/z)

LC/MS, HR/MS-6a

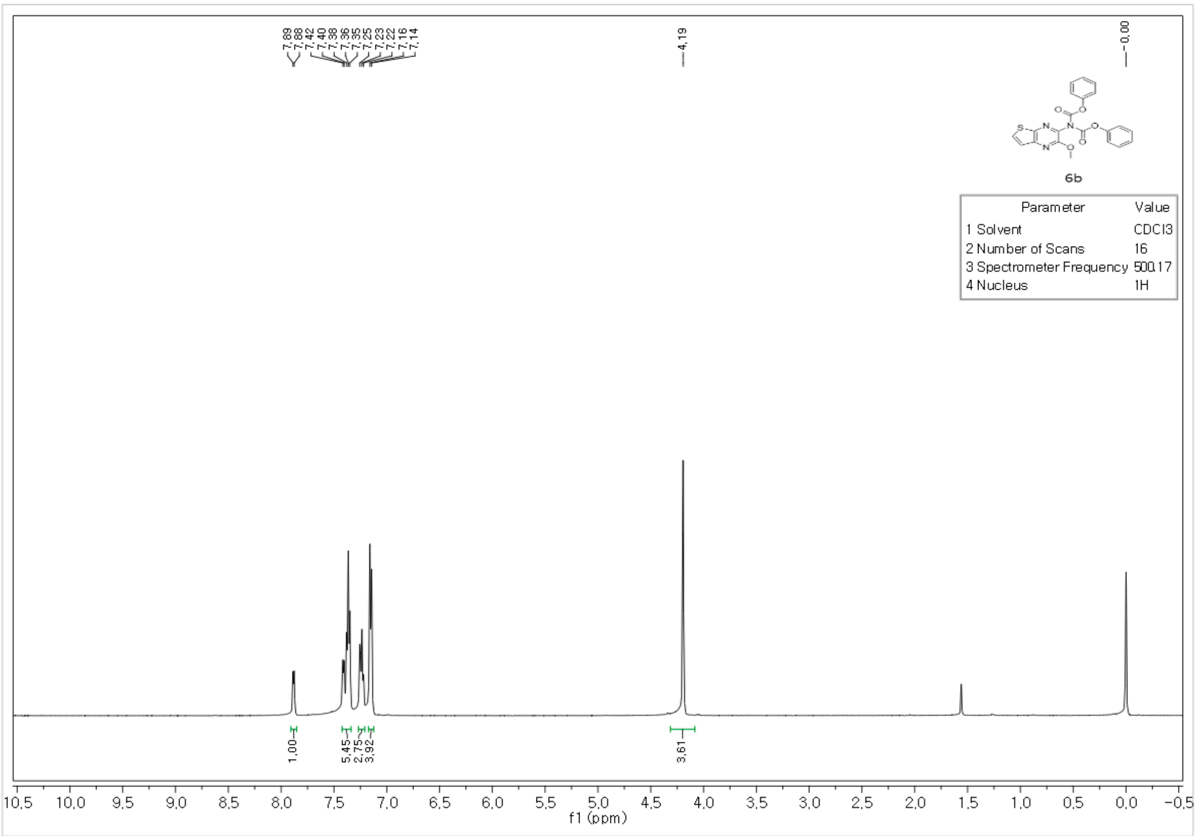

^{1}H-NMR-6b

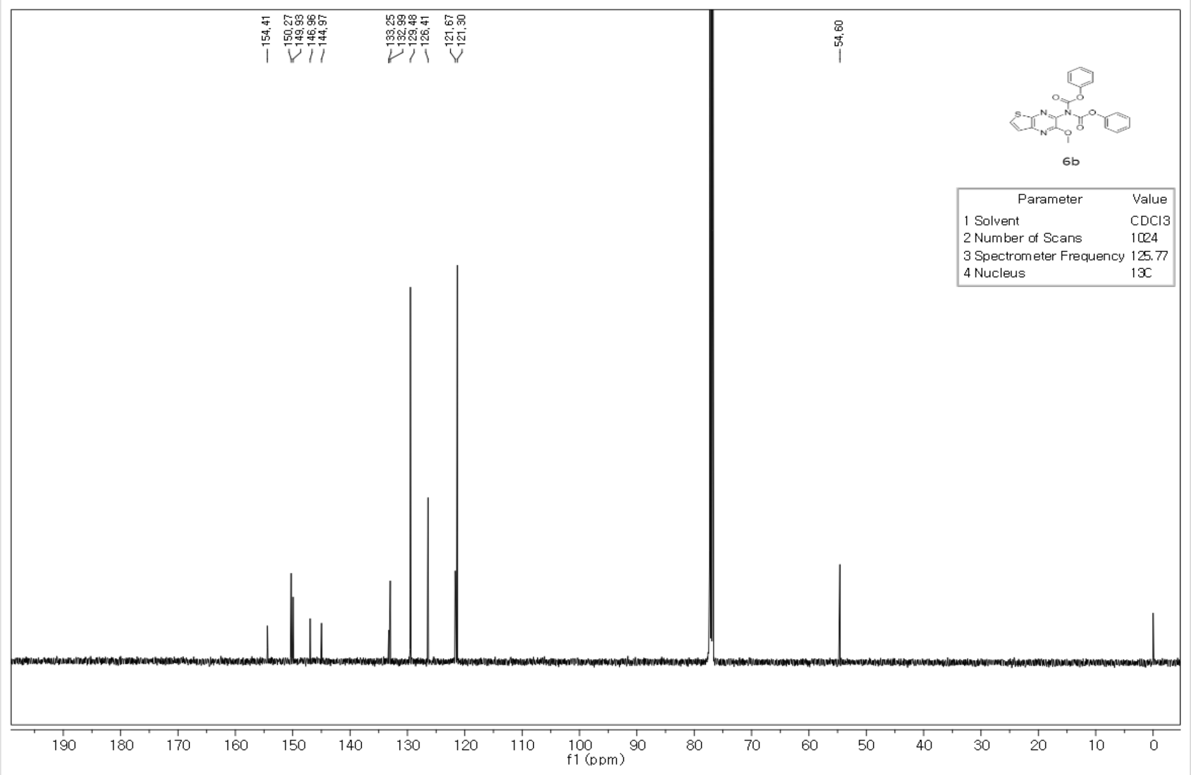

^{13}C-NMR-6b

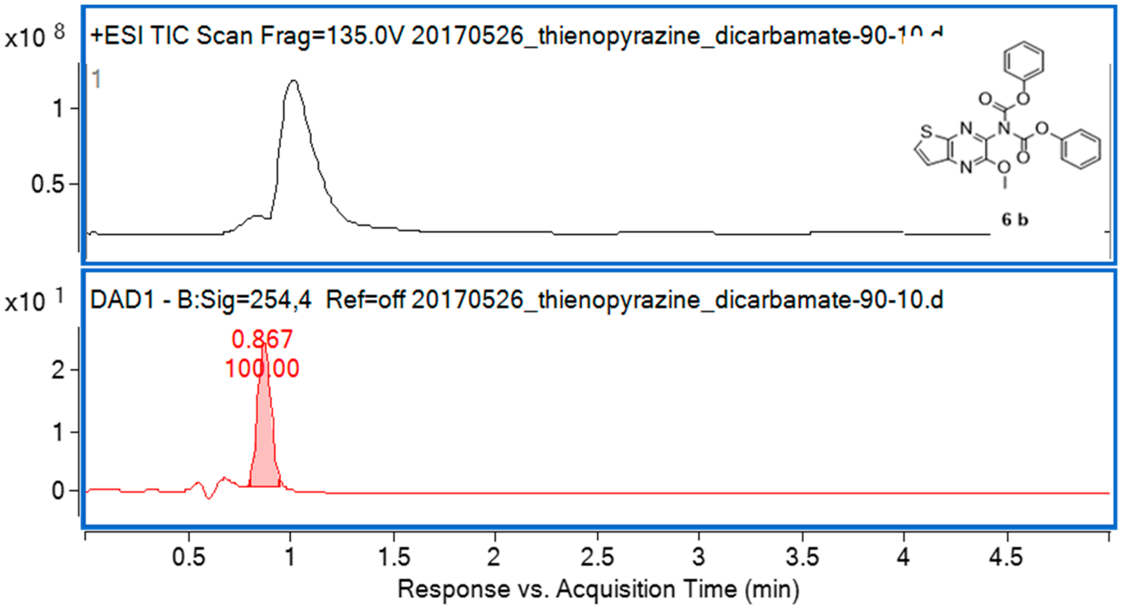
+ESI TIC Scan Frag=135.0V 20170526_thienopyrazine_dicarbamate-90-10.d
x10 8
6 b
DAD1 - B:Sig=254,4 Ref=off 20170526_thienopyrazine_dicarbamate-90-10.d
x10 1
0.867
100.00
Response vs. Acquisition Time (min)

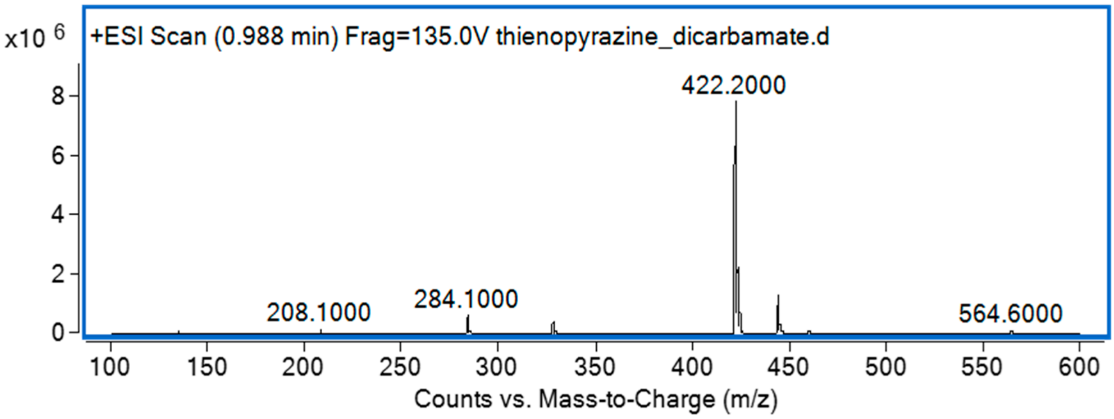
+ESI Scan (0.988 min) Frag=135.0V thienopyrazine_dicarbamate.d
x10 6
422.2000
208.1000
284.1000
564.6000
Counts vs. Mass-to-Charge (m/z)

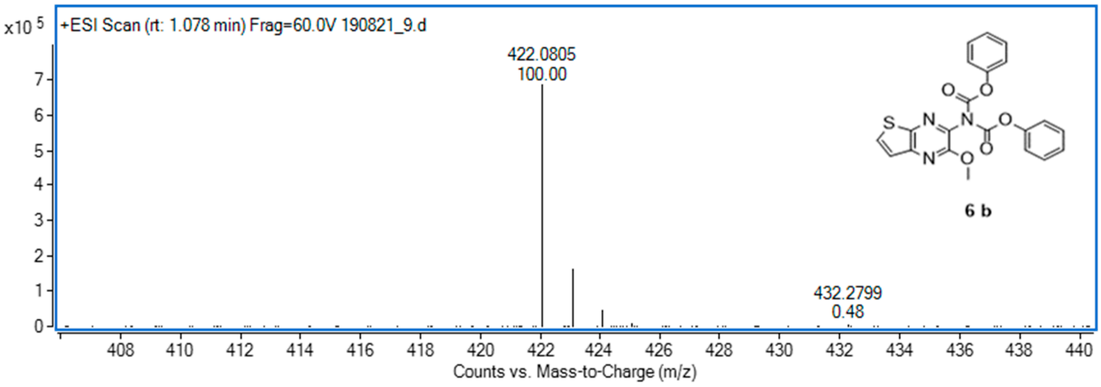
+ESI Scan (rt: 1.078 min) Frag=60.0V 190821_9.d
x10 5
422.0805
100.00
432.2799
0.48
6 b
Counts vs. Mass-to-Charge (m/z)

LC/MS, HR/MS-6b

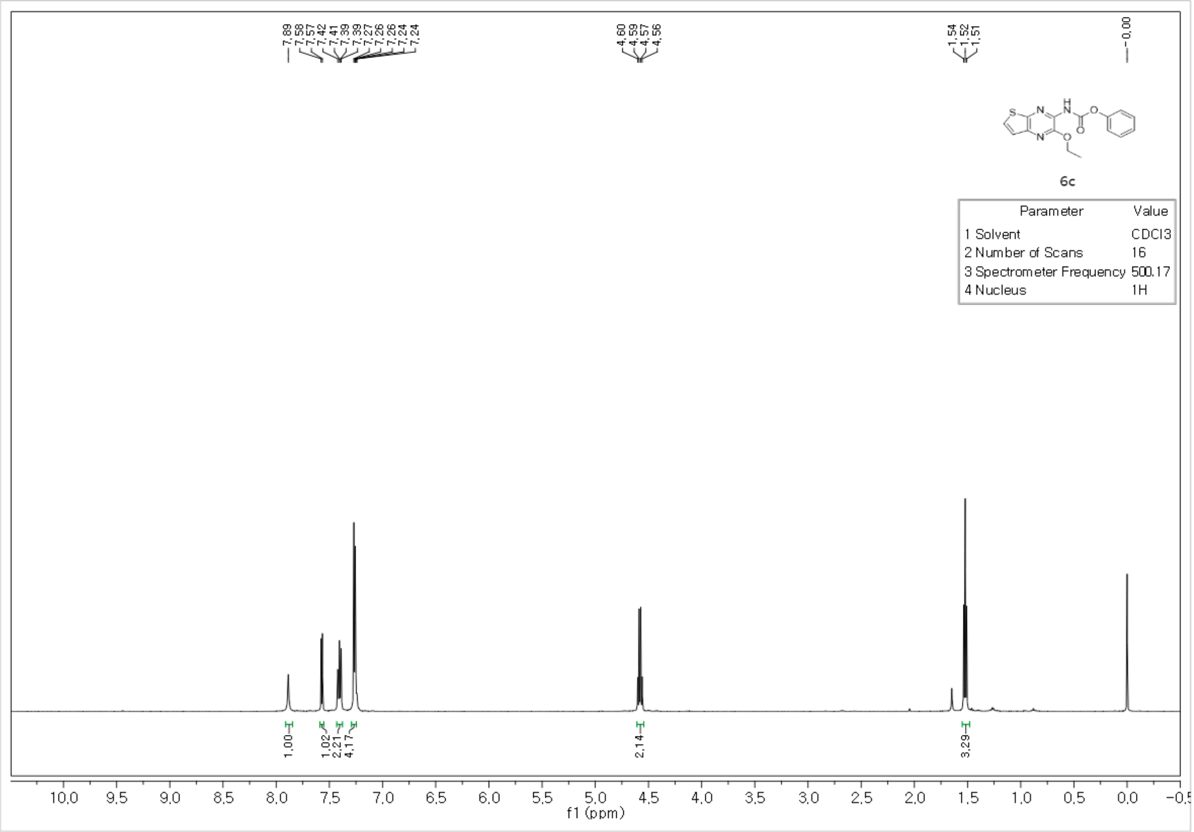

^{1}H-NMR-6c

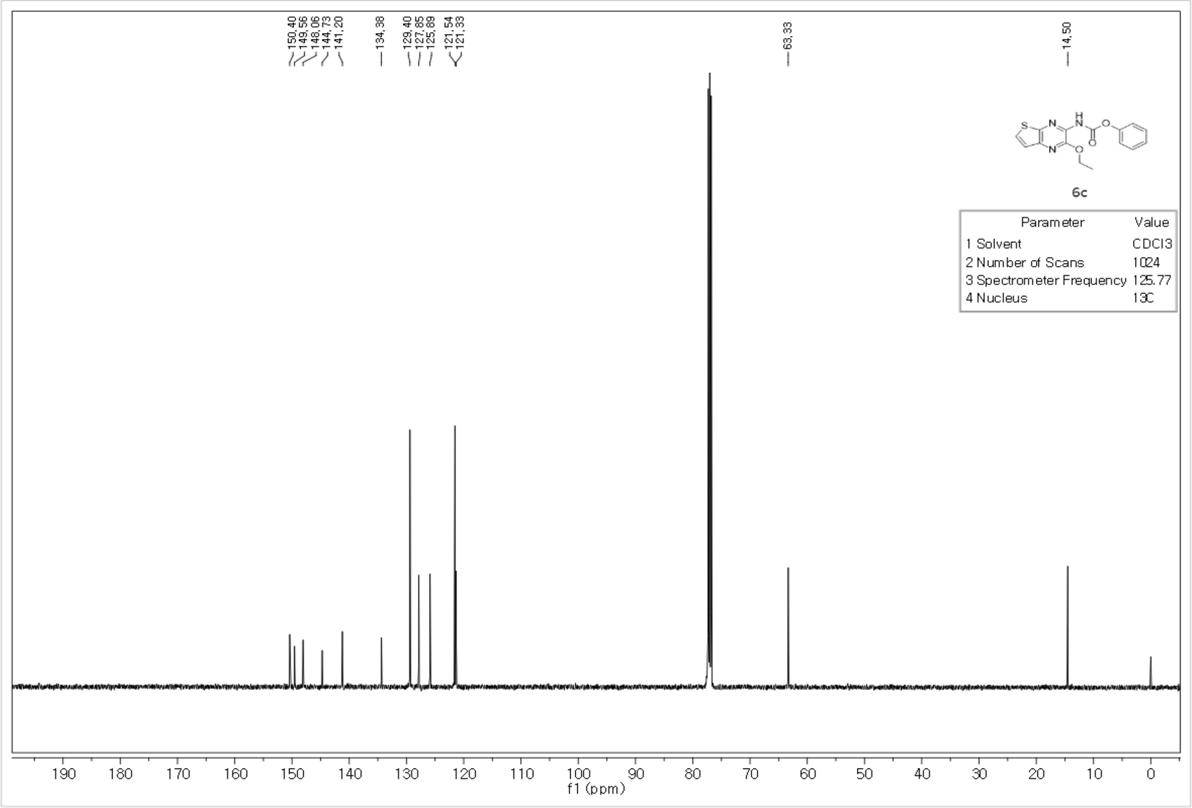

^{13}C-NMR-6c

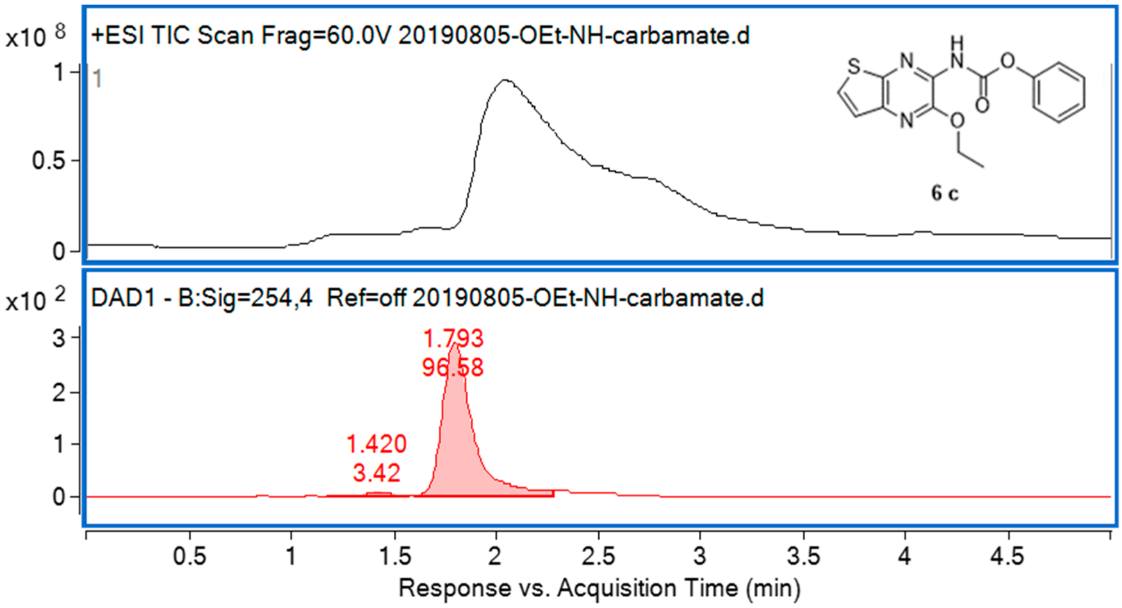
x10 8
+ESI TIC Scan Frag=60.0V 20190805-OEt-NH-carbamate.d
6 c
x10 2
DAD1 - B:Sig=254,4 Ref=off 20190805-OEt-NH-carbamate.d
1.793
96.58
1.420
3.42
Response vs. Acquisition Time (min)

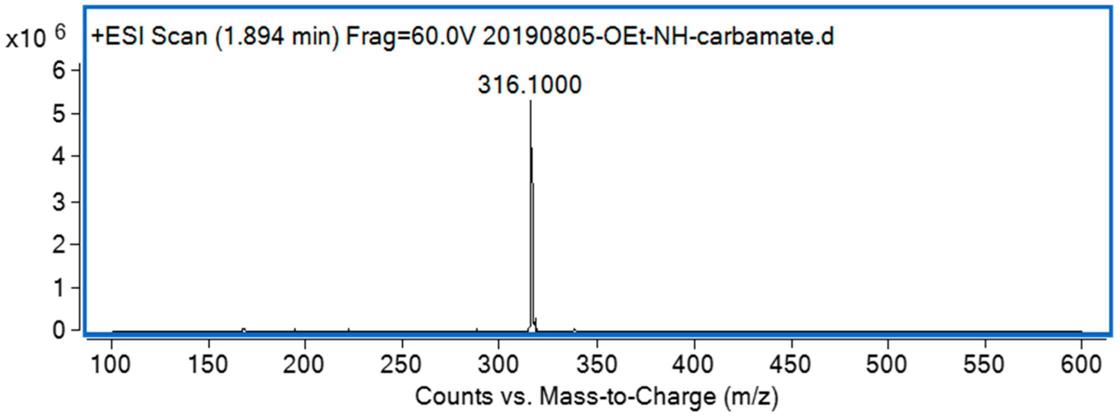
x10 6
+ESI Scan (1.894 min) Frag=60.0V 20190805-OEt-NH-carbamate.d
316.1000
Counts vs. Mass-to-Charge (m/z)

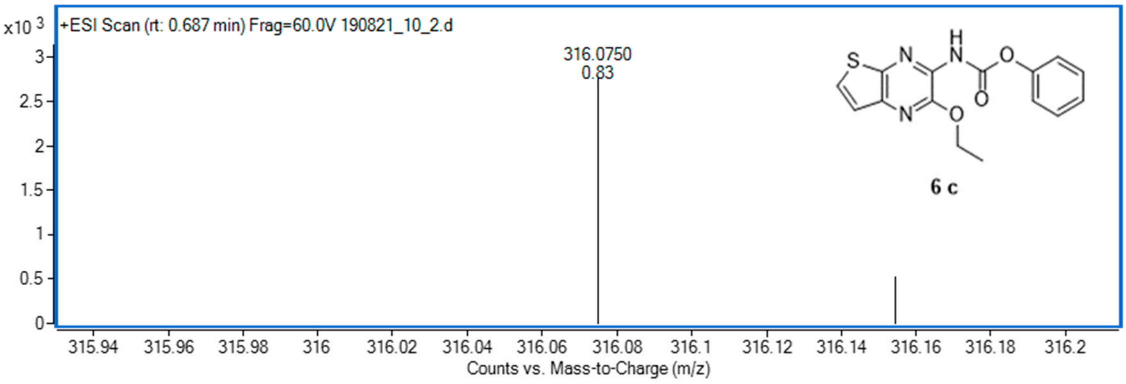
x10 3
+ESI Scan (rt: 0.687 min) Frag=60.0V 190821_10_2.d
316.0750
0.83
6 c
Counts vs. Mass-to-Charge (m/z)

LC/MS, HR/MS-6c

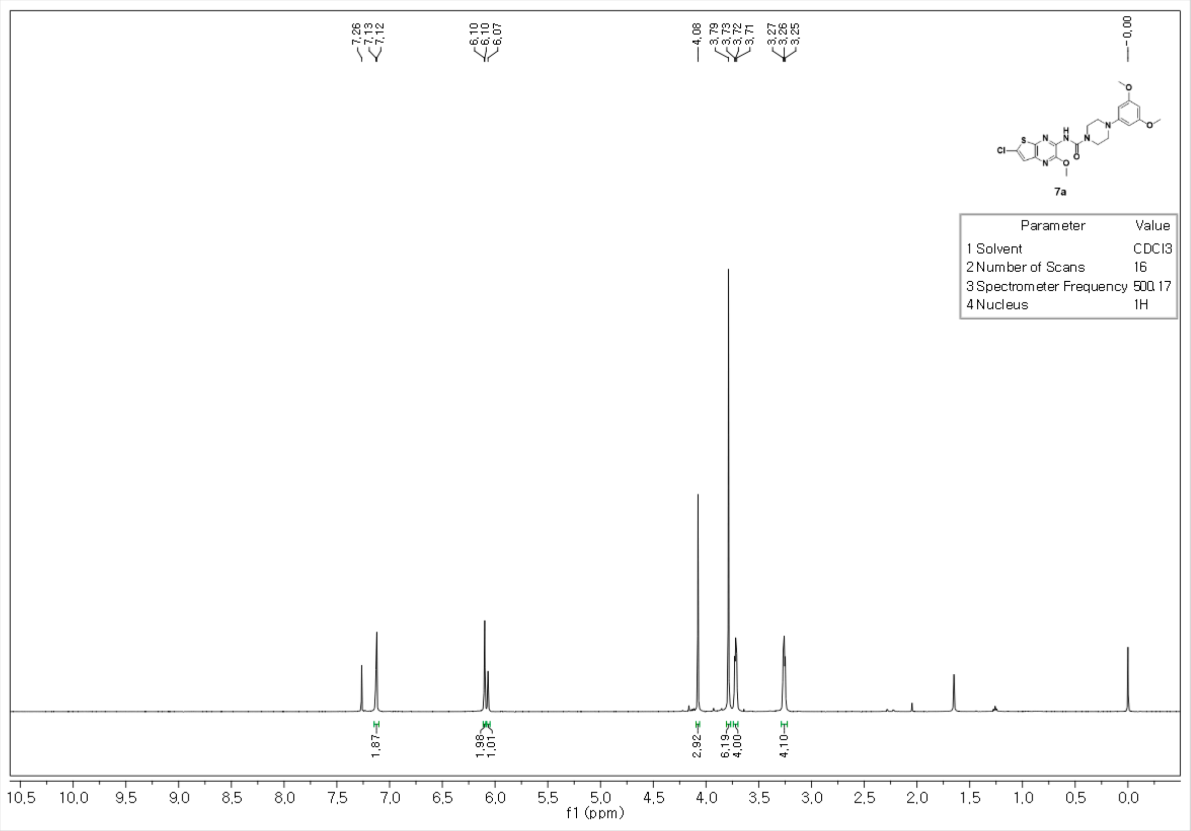

^{1}H-NMR-7a

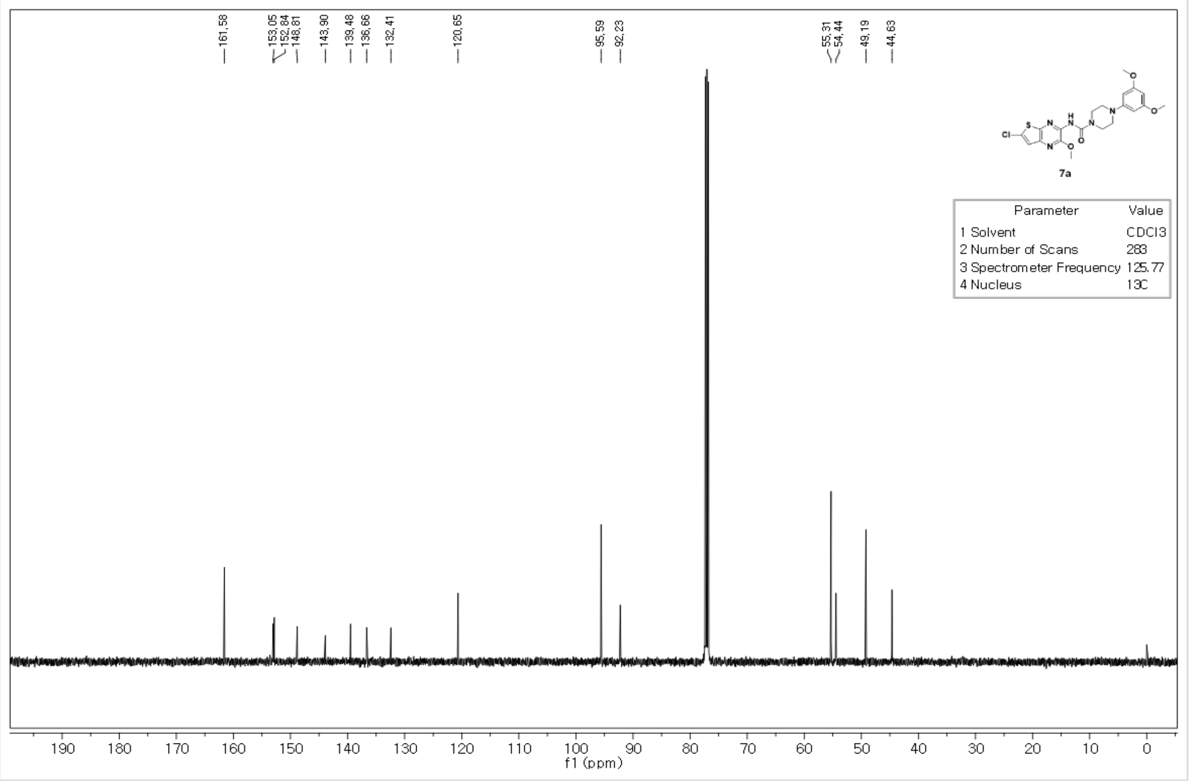

^{13}C-NMR-7a

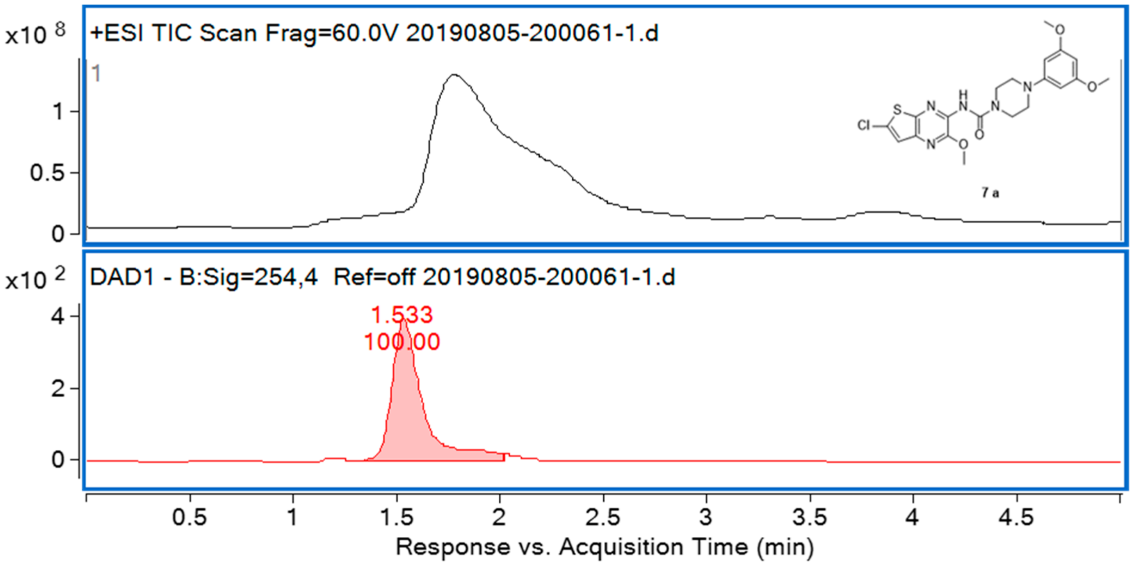
x10 8
+ESI TIC Scan Frag=60.0V 20190805-200061-1.d
7 a
x10 2
DAD1 - B:Sig=254,4 Ref=off 20190805-200061-1.d
1.533
100.00
Response vs. Acquisition Time (min)

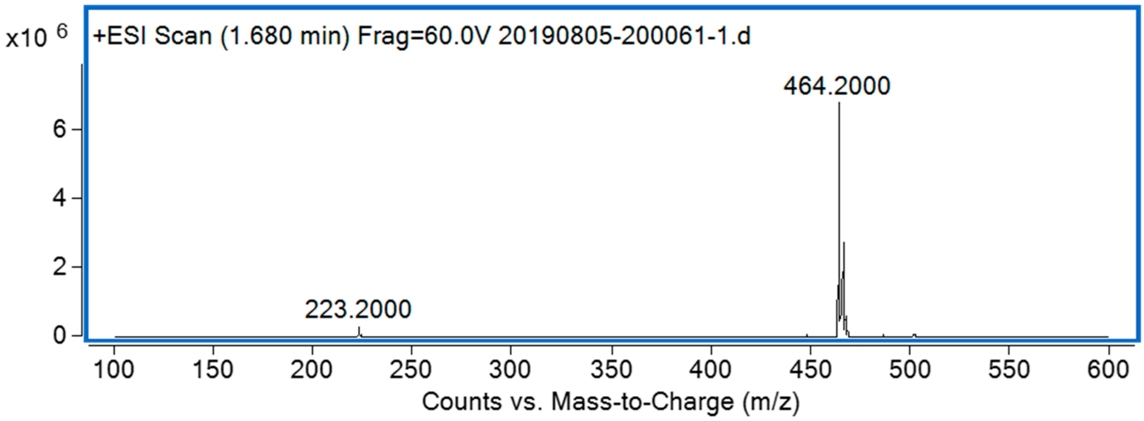
x10 6
+ESI Scan (1.680 min) Frag=60.0V 20190805-200061-1.d
464.2000
223.2000
Counts vs. Mass-to-Charge (m/z)

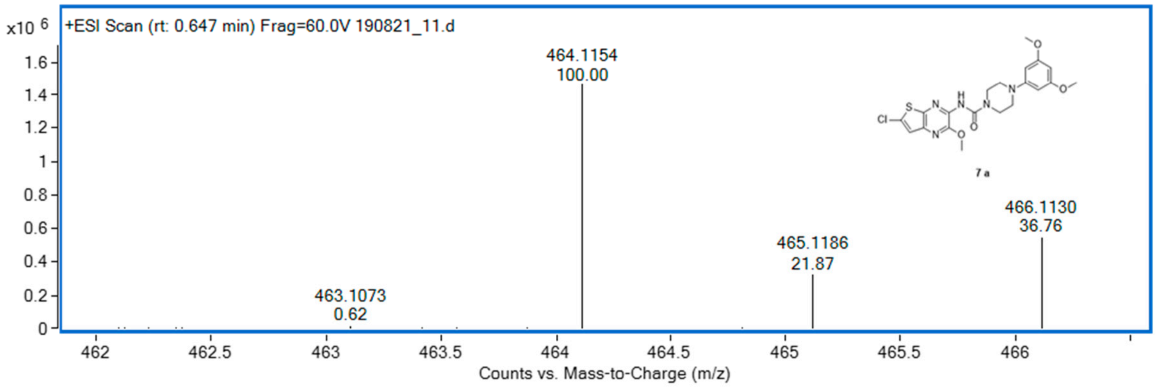
x10 6
+ESI Scan (rt: 0.647 min) Frag=60.0V 190821_11.d
464.1154
100.00
7 a
466.1130
36.76
465.1186
21.87
463.1073
0.62
Counts vs. Mass-to-Charge (m/z)

LC/MS, HR/MS-7a

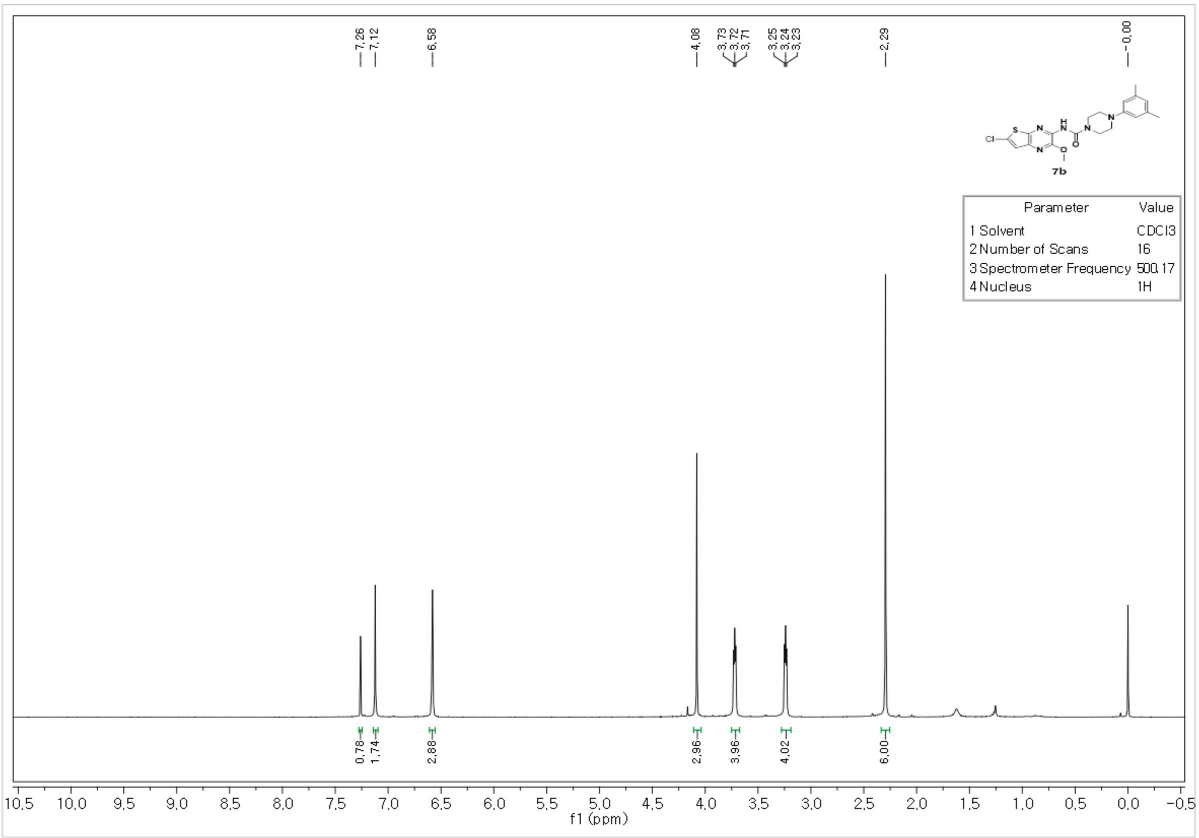

^{1}H-NMR-7b

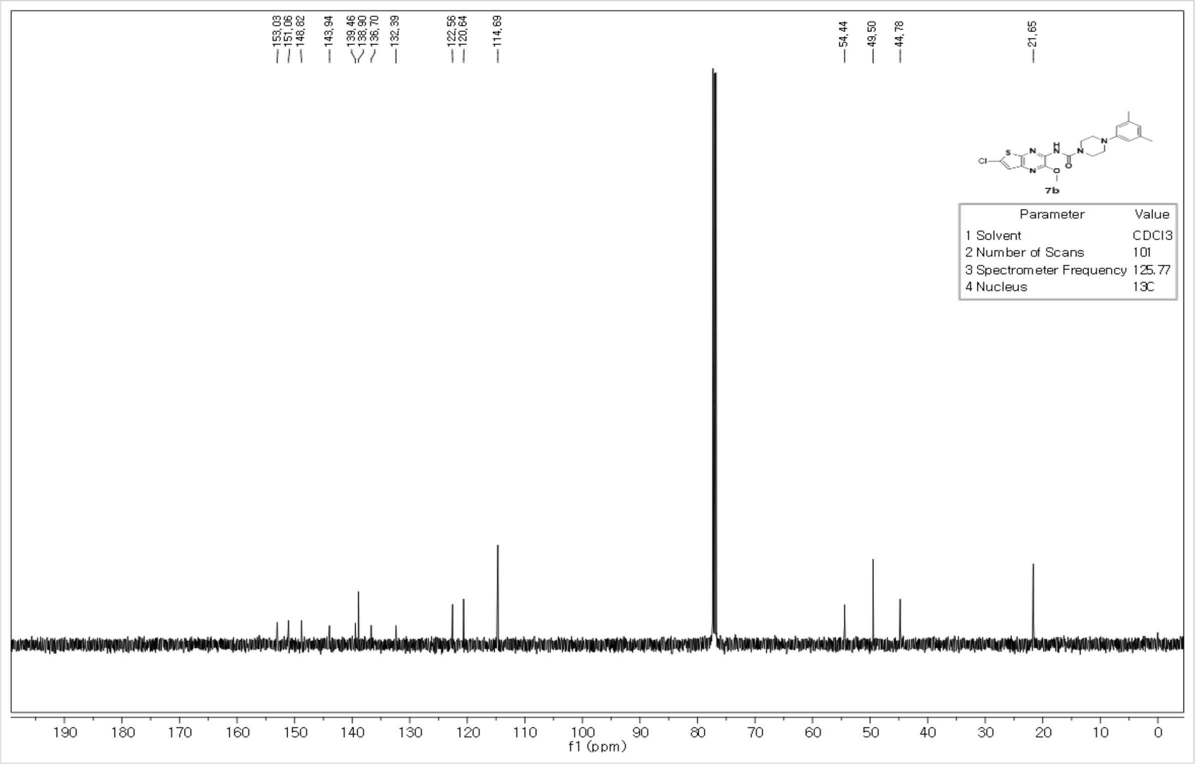

^{13}C-NMR-7b

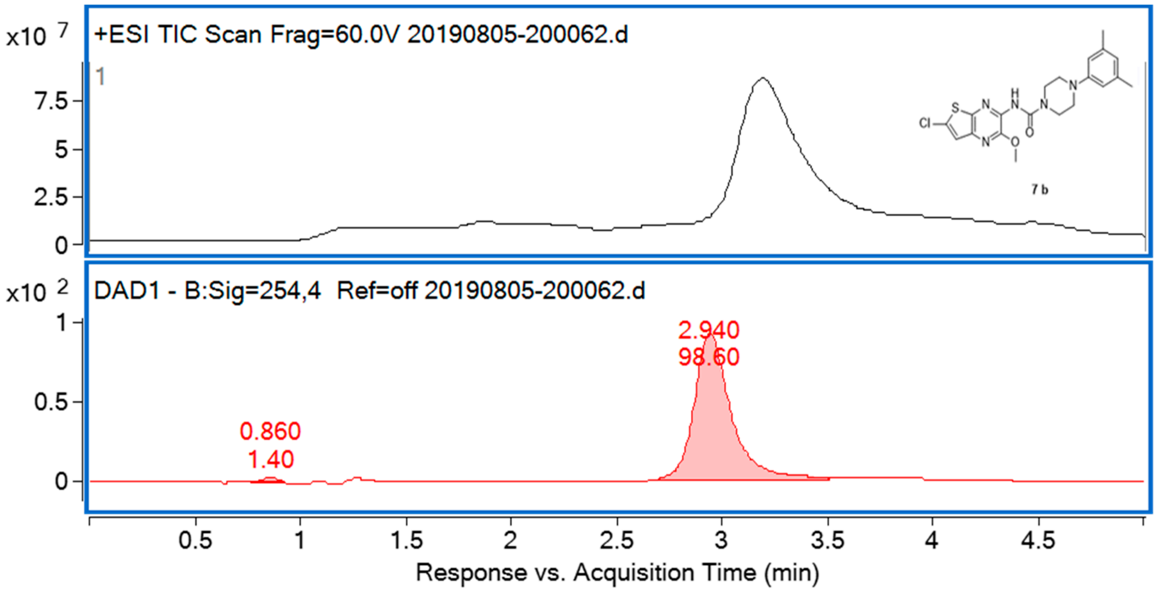
+ESI TIC Scan Frag=60.0V 20190805-200062.d
DAD1 - B:Sig=254,4 Ref=off 20190805-200062.d
2.940
98.60
0.860
1.40
Cl
S
N
N
H
N
O
O
N
N
7b
Response vs. Acquisition Time (min)

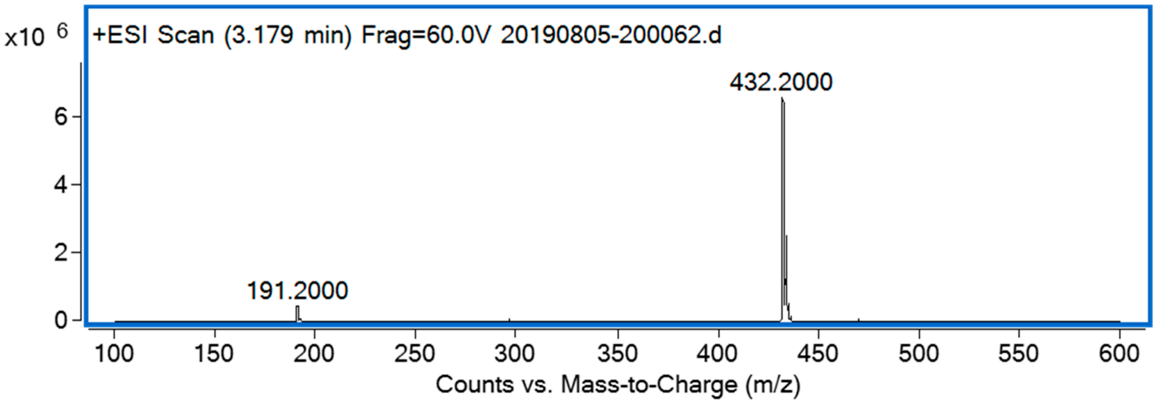
+ESI Scan (3.179 min) Frag=60.0V 20190805-200062.d
432.2000
191.2000
Counts vs. Mass-to-Charge (m/z)

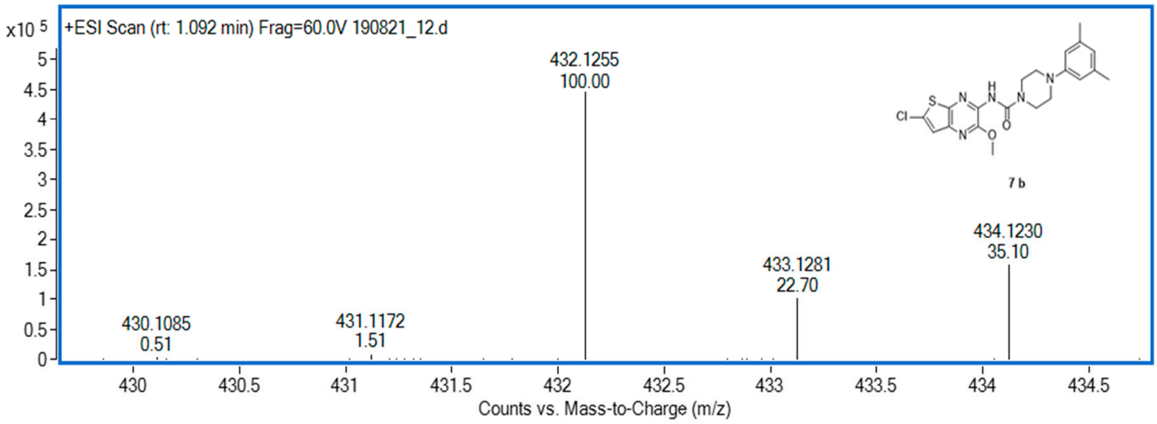
+ESI Scan (rt: 1.092 min) Frag=60.0V 190821_12.d
432.1255
100.00
433.1281
22.70
434.1230
35.10
430.1085
0.51
431.1172
1.51
7b
Counts vs. Mass-to-Charge (m/z)

LC/MS, HR/MS-7b

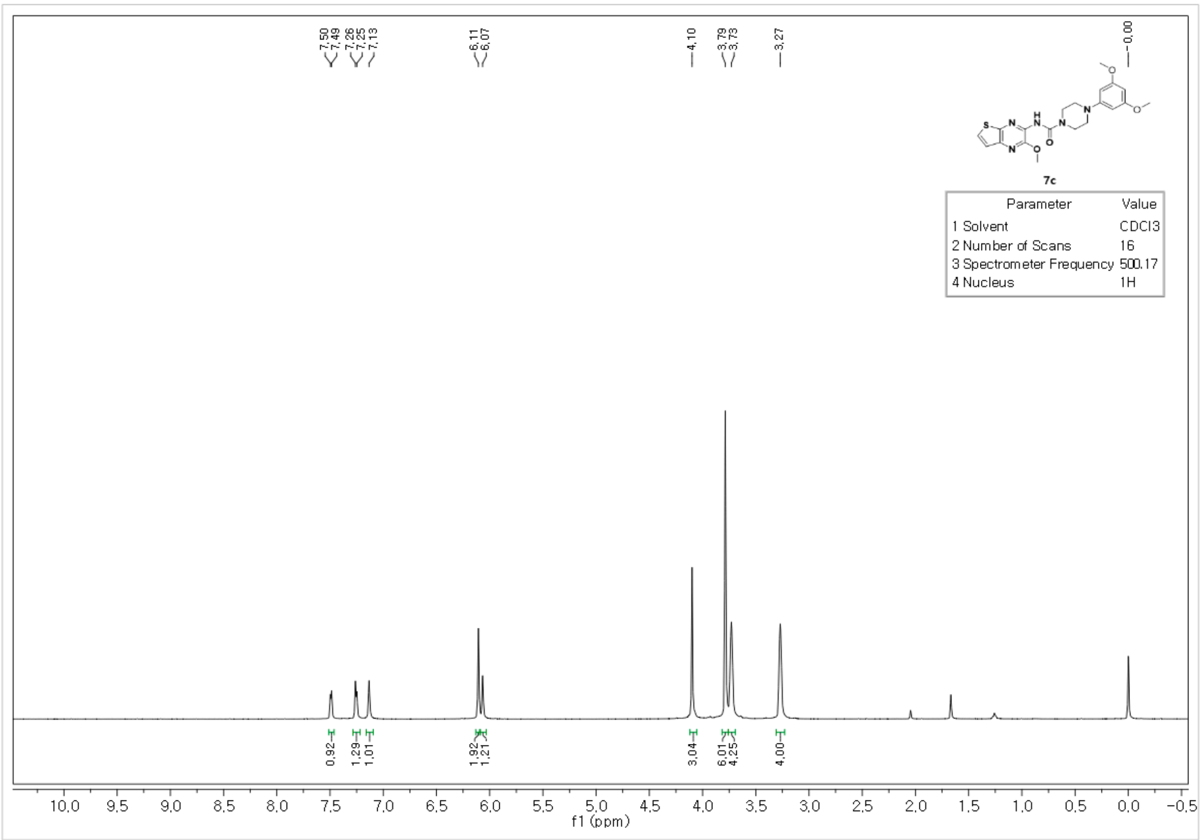

^{1}H-NMR-7c

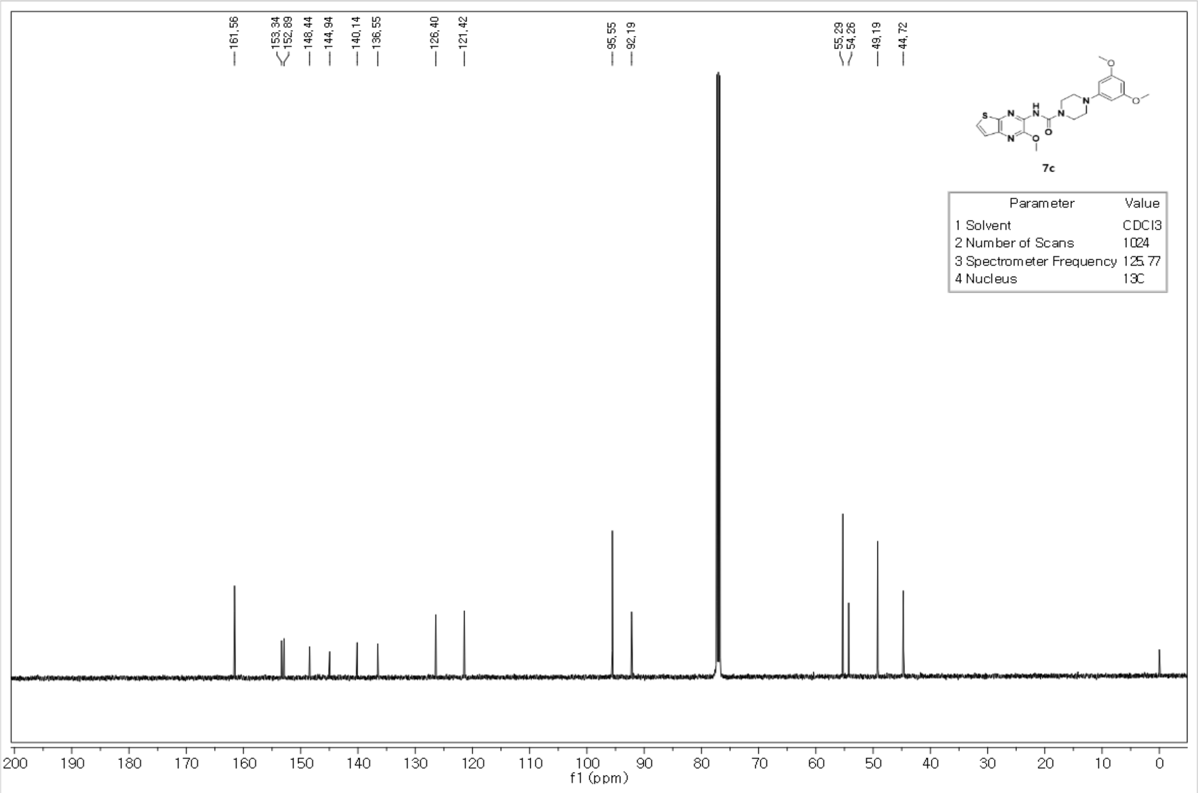

^{13}C-NMR-7c

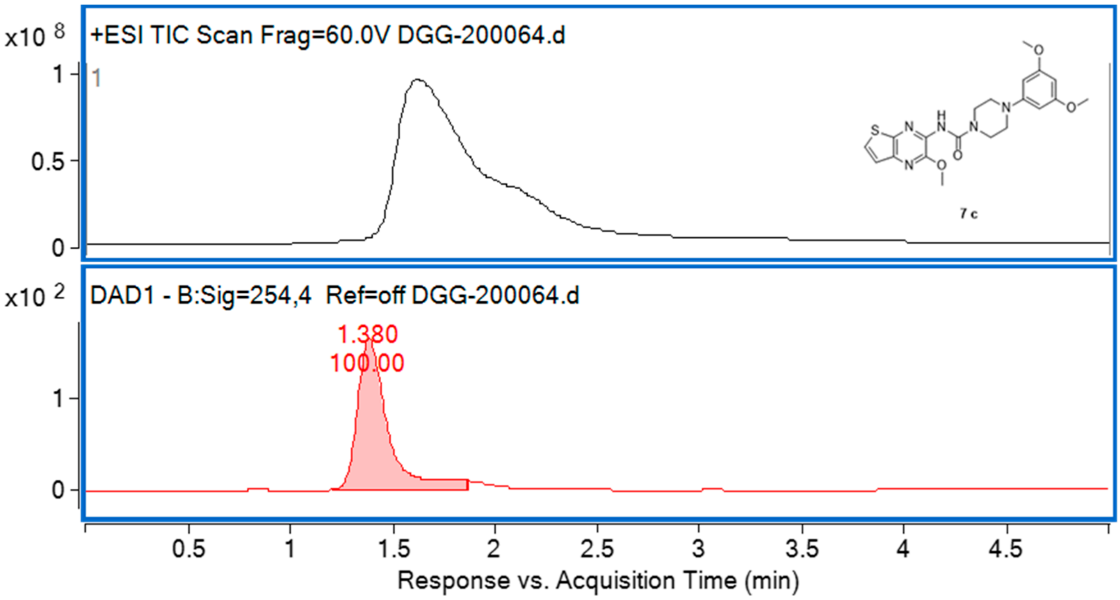
x10 8
+ESI TIC Scan Frag=60.0V DGG-200064.d
7c
x10 2
DAD1 - B:Sig=254,4 Ref=off DGG-200064.d
1.380
100.00
Response vs. Acquisition Time (min)

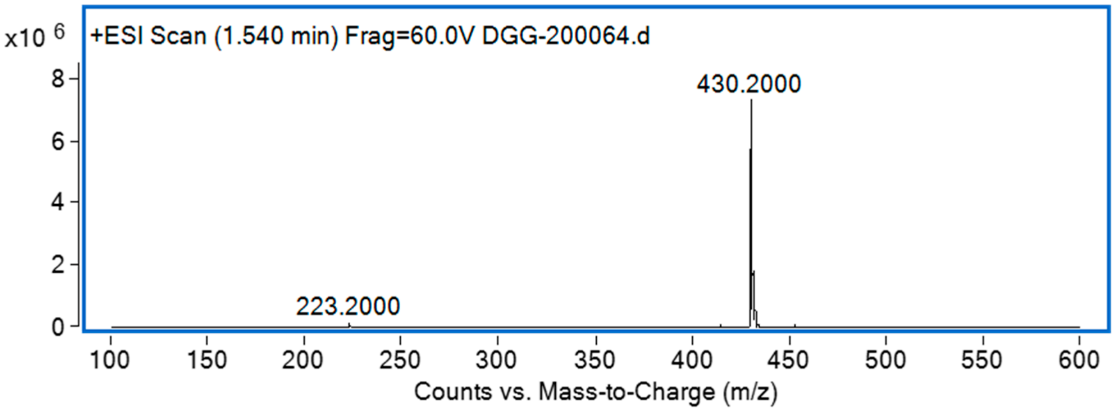
x10 6
+ESI Scan (1.540 min) Frag=60.0V DGG-200064.d
430.2000
223.2000
Counts vs. Mass-to-Charge (m/z)

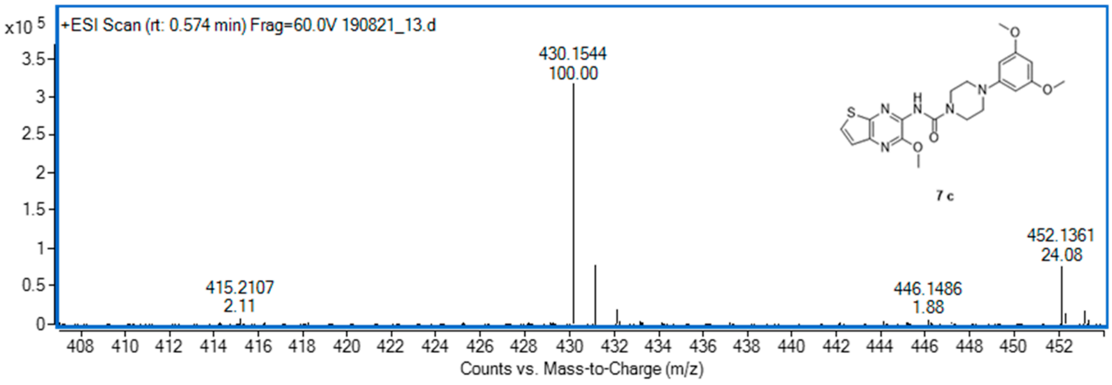
x10 5
+ESI Scan (rt: 0.574 min) Frag=60.0V 190821_13.d
430.1544
100.00
7c
452.1361
24.08
415.2107
2.11
446.1486
1.88
Counts vs. Mass-to-Charge (m/z)

LC/MS, HR/MS-7c

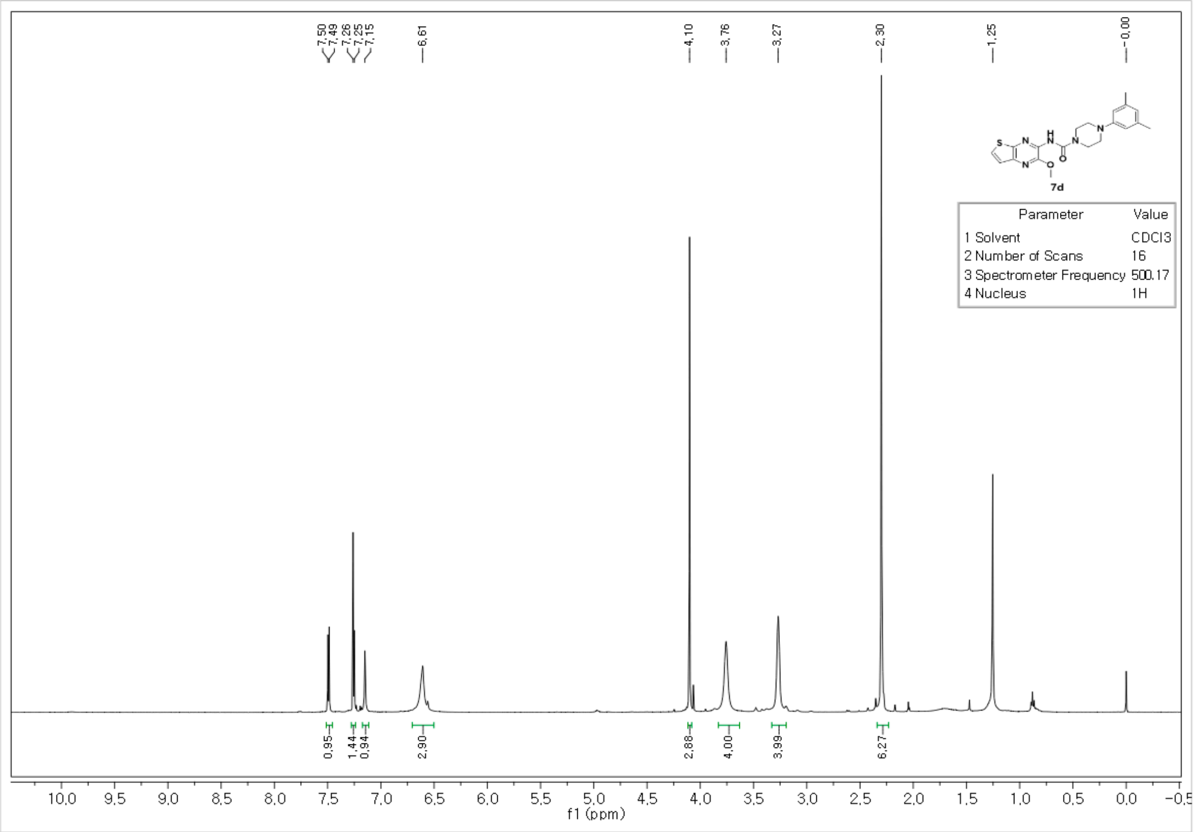

^{1}H-NMR-7d

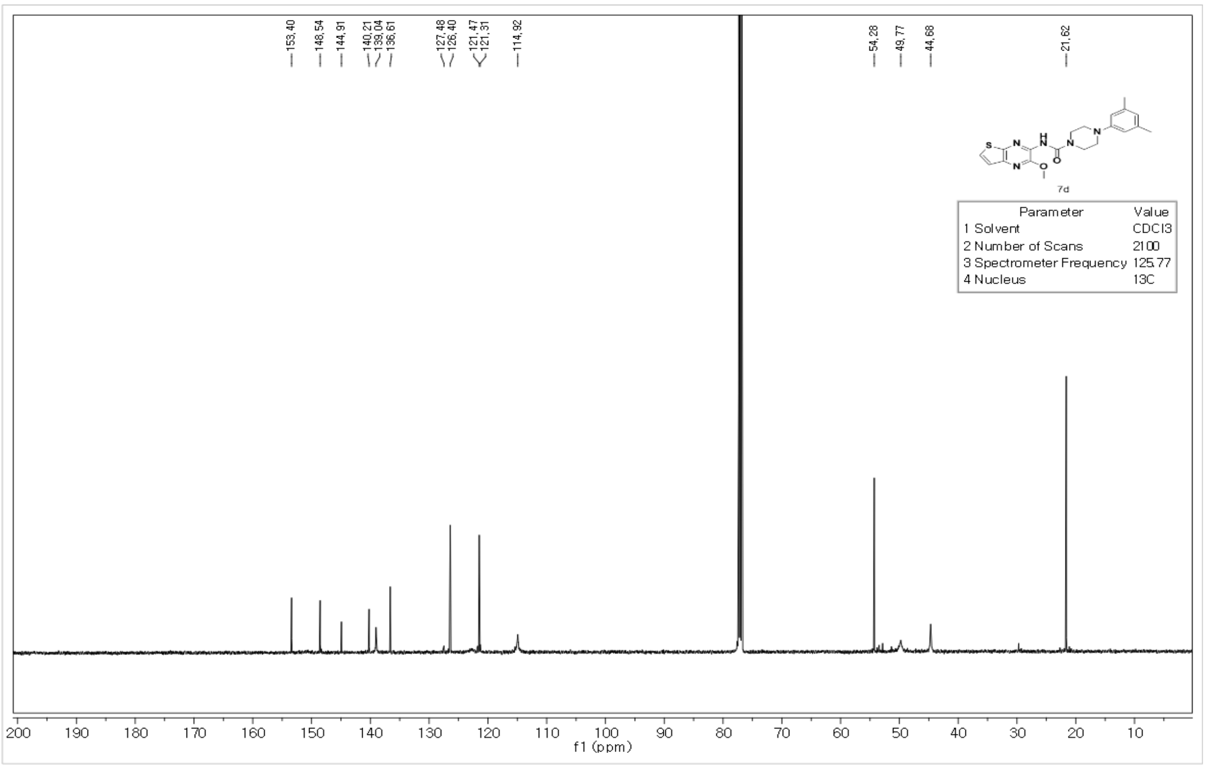

^{13}C-NMR-7d

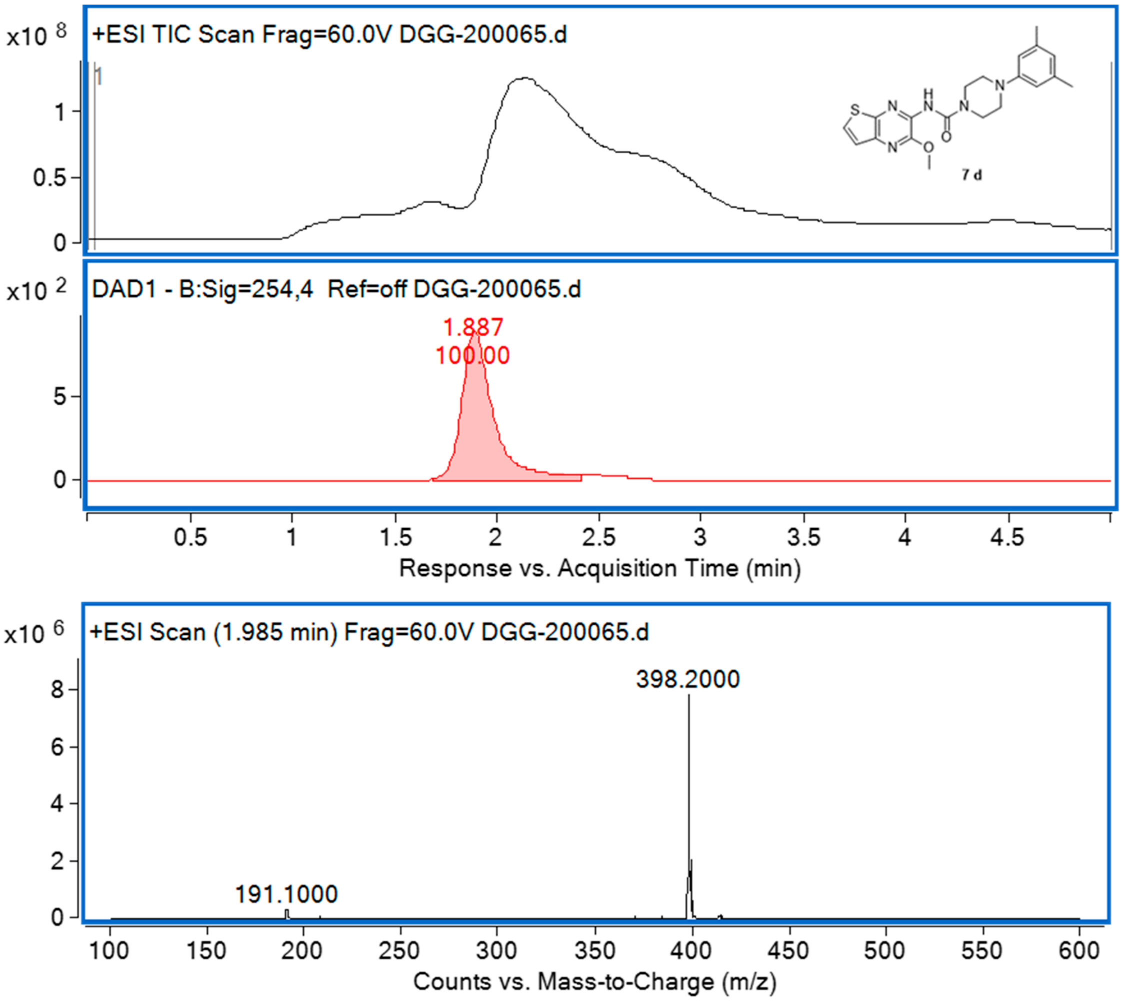
x10 8
+ESI TIC Scan Frag=60.0V DGG-200065.d
7 d
x10 2
DAD1 - B:Sig=254,4 Ref=off DGG-200065.d
1.887
100.00
Response vs. Acquisition Time (min)
x10 6
+ESI Scan (1.985 min) Frag=60.0V DGG-200065.d
398.2000
191.1000
Counts vs. Mass-to-Charge (m/z)

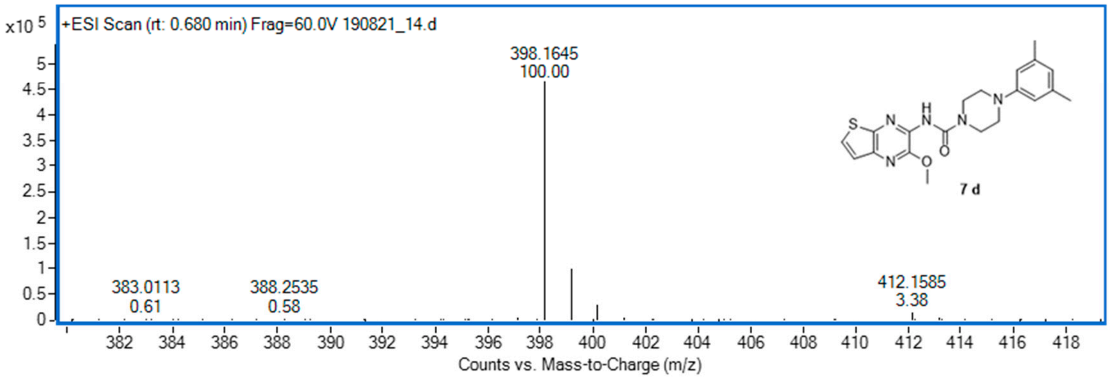
x10 5
+ESI Scan (rt: 0.680 min) Frag=60.0V 190821_14.d
398.1645
100.00
383.0113
0.61
388.2535
0.58
412.1585
3.38
7 d
Counts vs. Mass-to-Charge (m/z)

LC/MS, HR/MS-7d

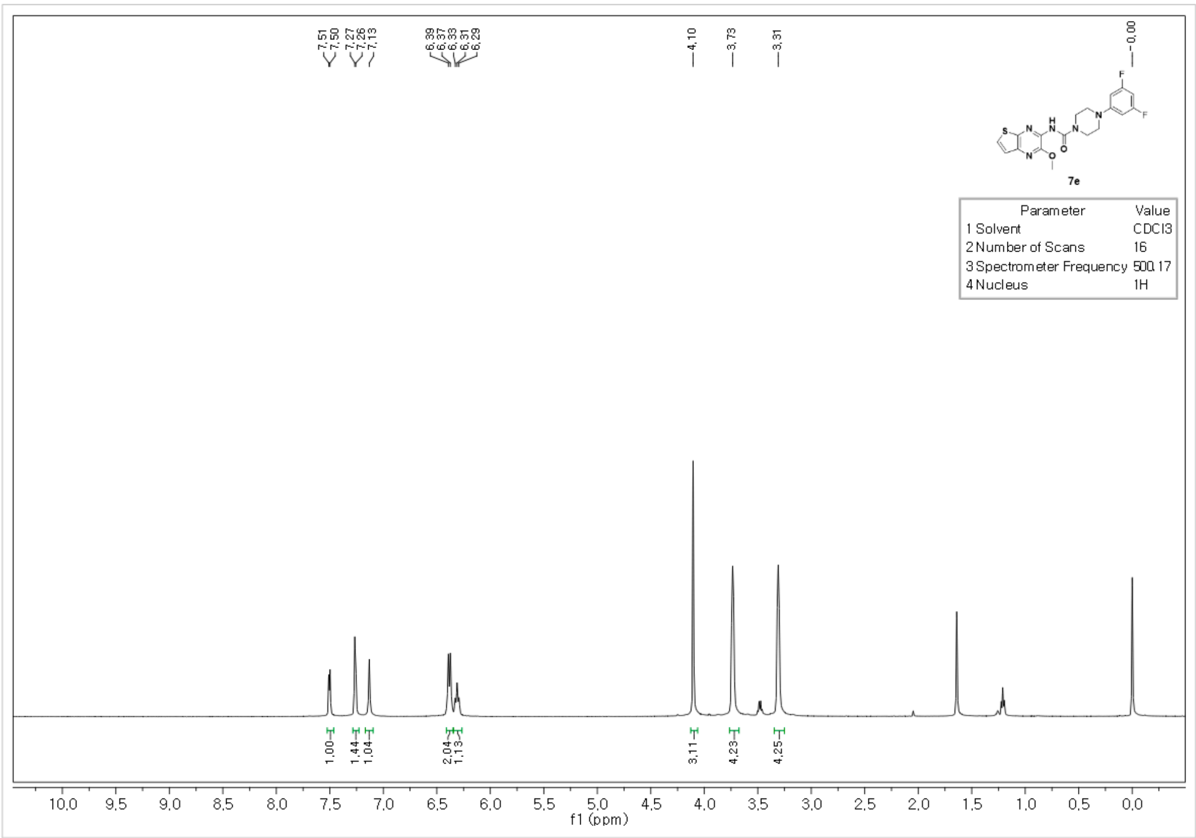

^{1}H-NMR-7e

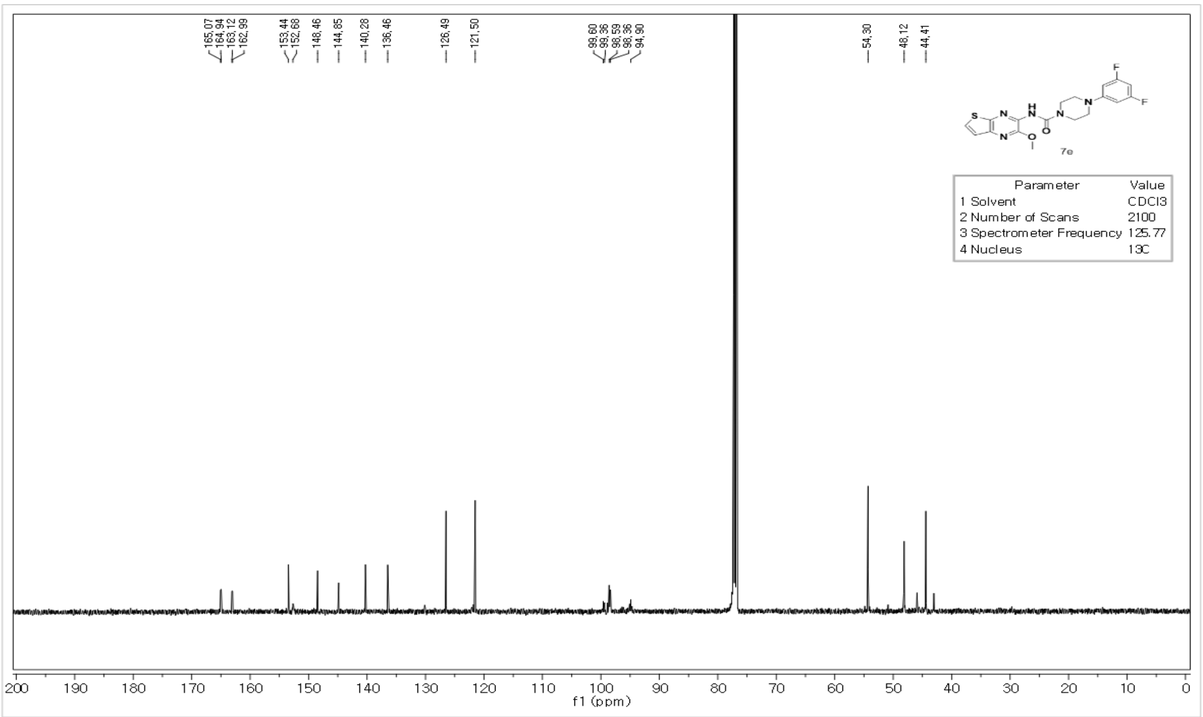

^{13}C-NMR-7e

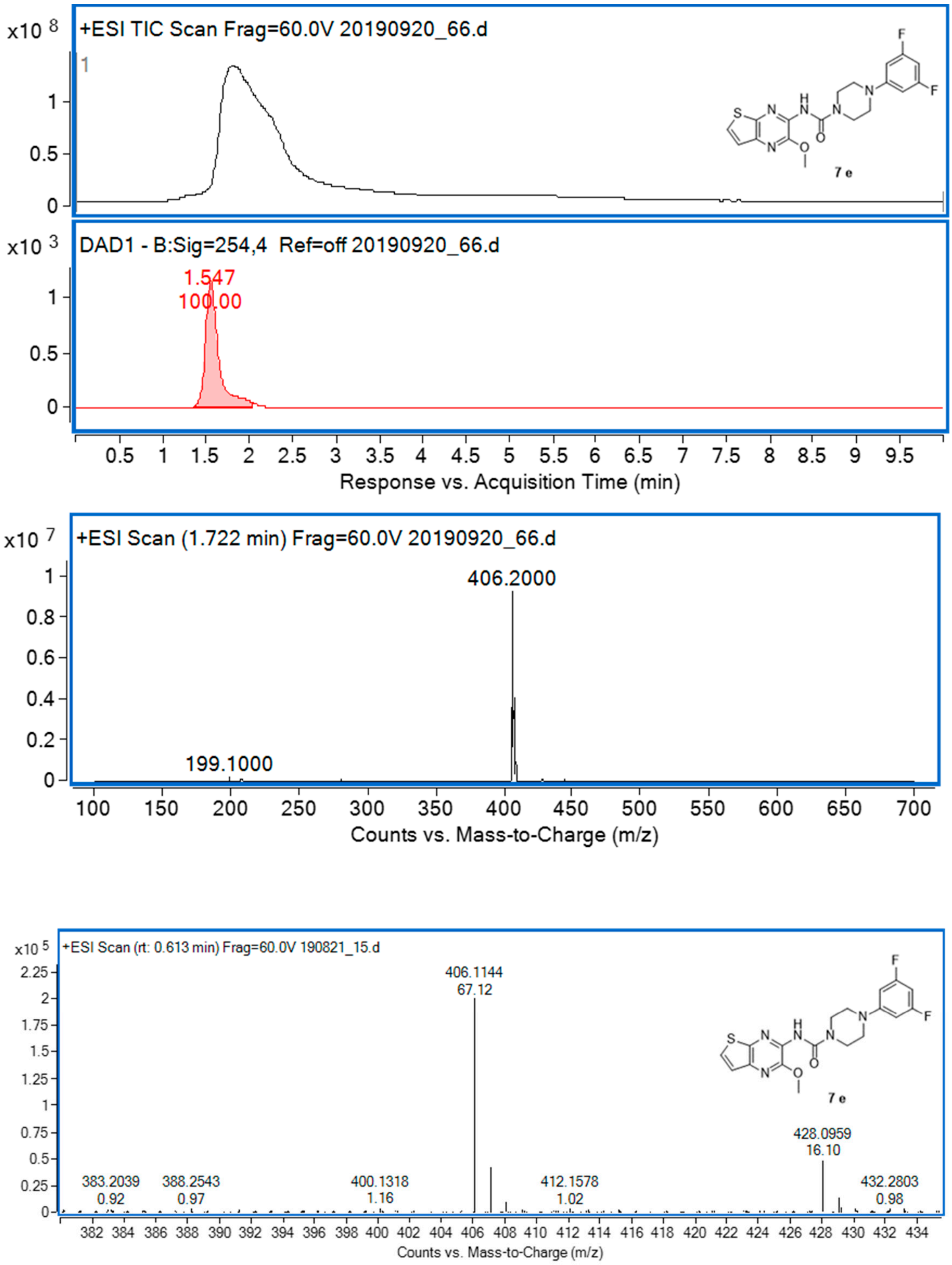

+ESI TIC Scan Frag=60.0V 20190920_66.d
x10 8
DAD1 - B:Sig=254,4 Ref=off 20190920_66.d
x10 3
1.547
100.00
Response vs. Acquisition Time (min)
+ESI Scan (1.722 min) Frag=60.0V 20190920_66.d
x10 7
406.2000
199.1000
Counts vs. Mass-to-Charge (m/z)
+ESI Scan (rt: 0.613 min) Frag=60.0V 190821_15.d
x10 5
406.1144
67.12
428.0959
16.10
383.2039
0.92
388.2543
0.97
400.1318
1.16
412.1578
1.02
432.2803
0.98
Counts vs. Mass-to-Charge (m/z)
7e

LC/MS, HR/MS-7e

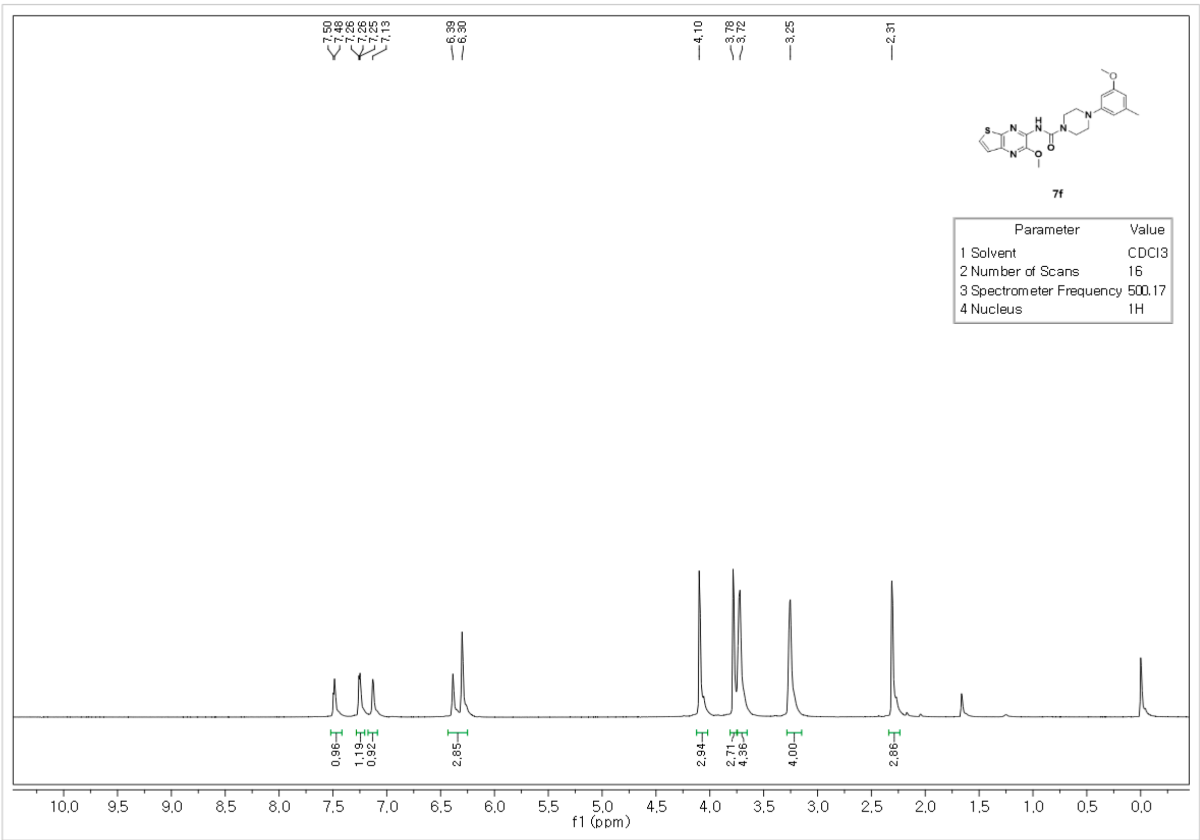

^{1}H-NMR-7f

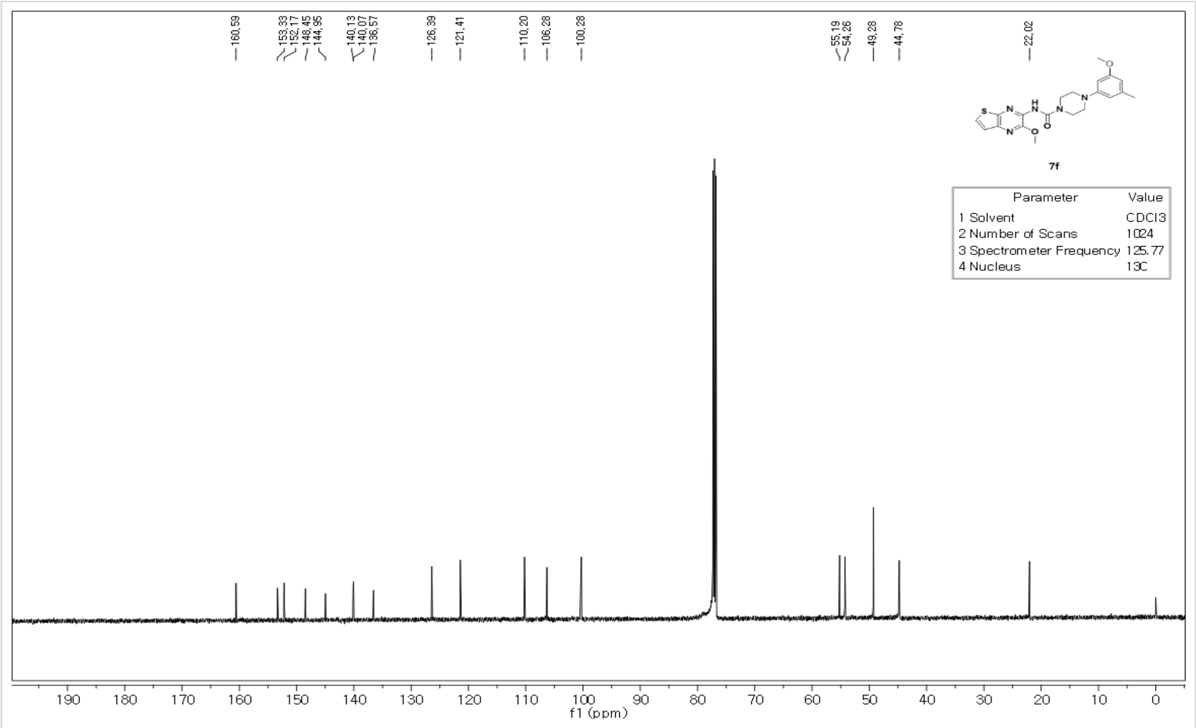

^{13}C-NMR-7f

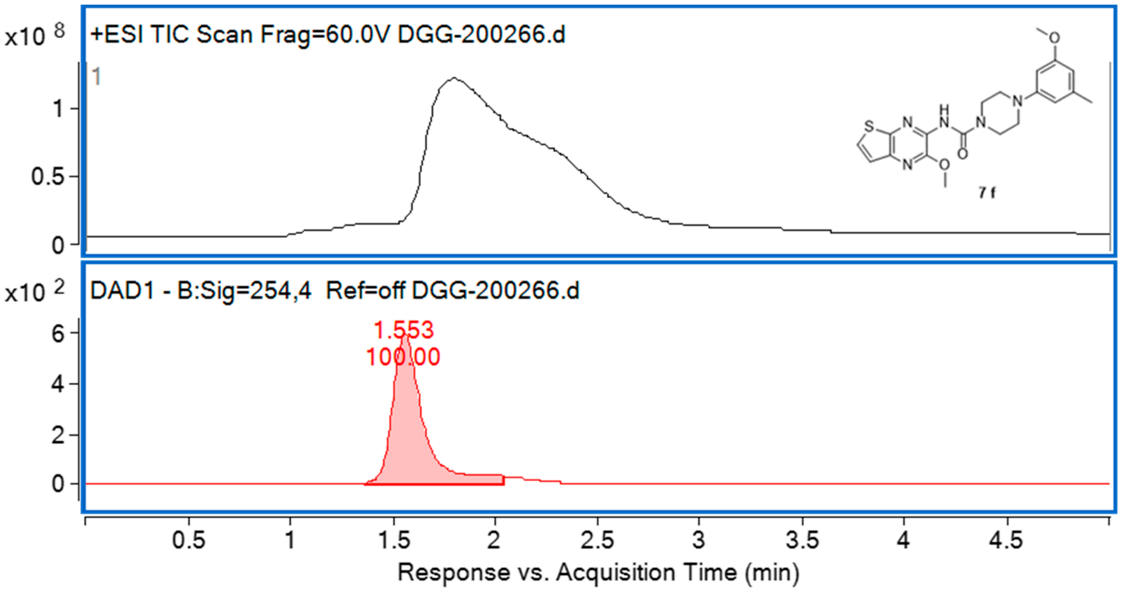
x10 8
+ESI TIC Scan Frag=60.0V DGG-200266.d
7f
x10 2
DAD1 - B:Sig=254,4 Ref=off DGG-200266.d
1.553
100.00
Response vs. Acquisition Time (min)

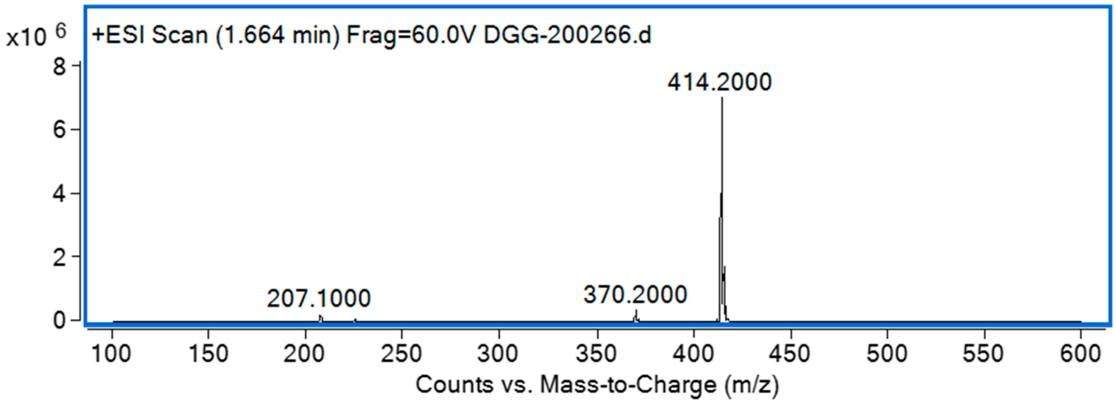
x10 6
+ESI Scan (1.664 min) Frag=60.0V DGG-200266.d
414.2000
207.1000
370.2000
Counts vs. Mass-to-Charge (m/z)

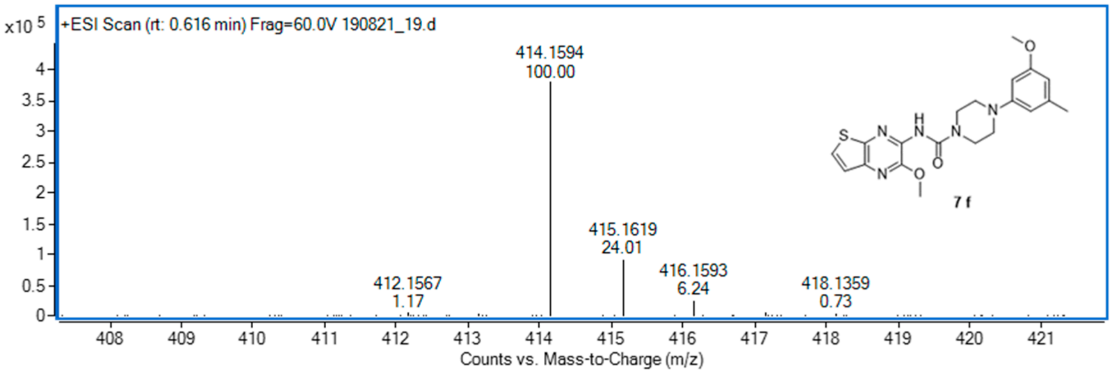
x10 5
+ESI Scan (rt: 0.616 min) Frag=60.0V 190821_19.d
414.1594
100.00
415.1619
24.01
416.1593
6.24
412.1567
1.17
418.1359
0.73
7f
Counts vs. Mass-to-Charge (m/z)

LC/MS, HR/MS-7f

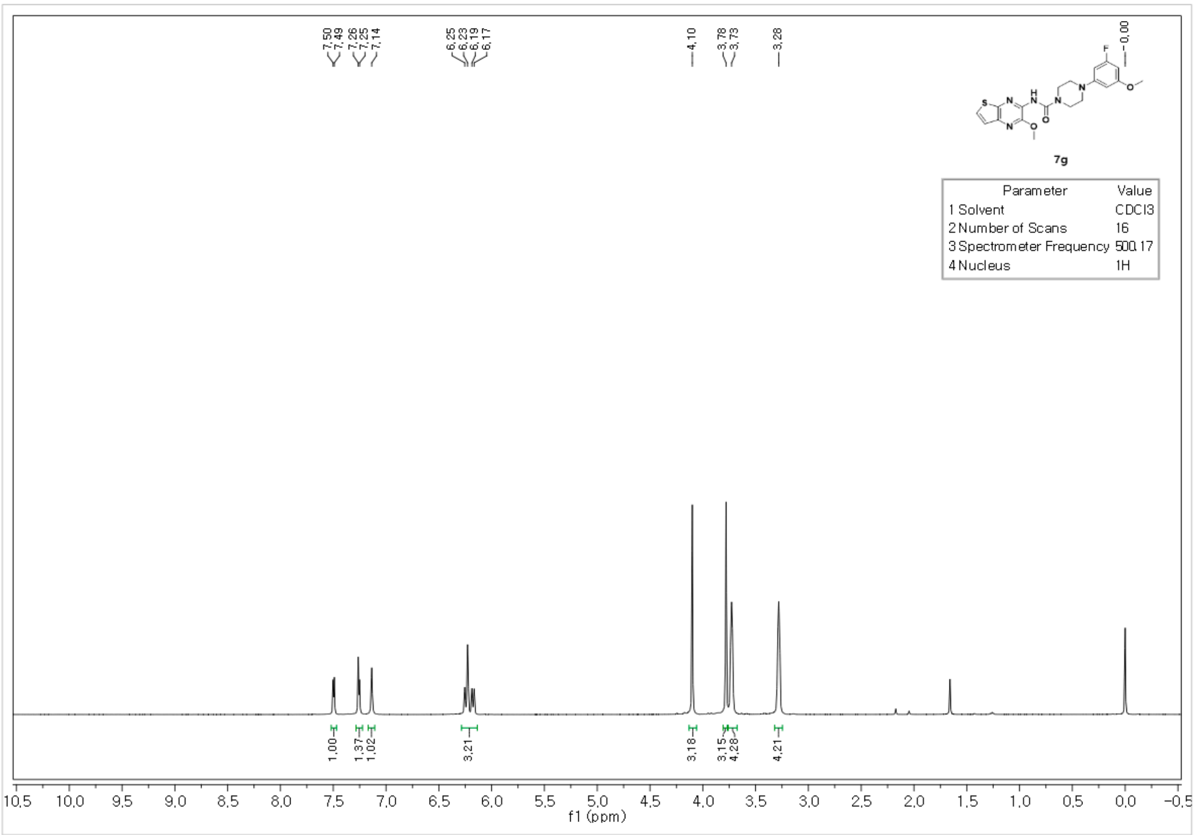

^{1}H-NMR-7g

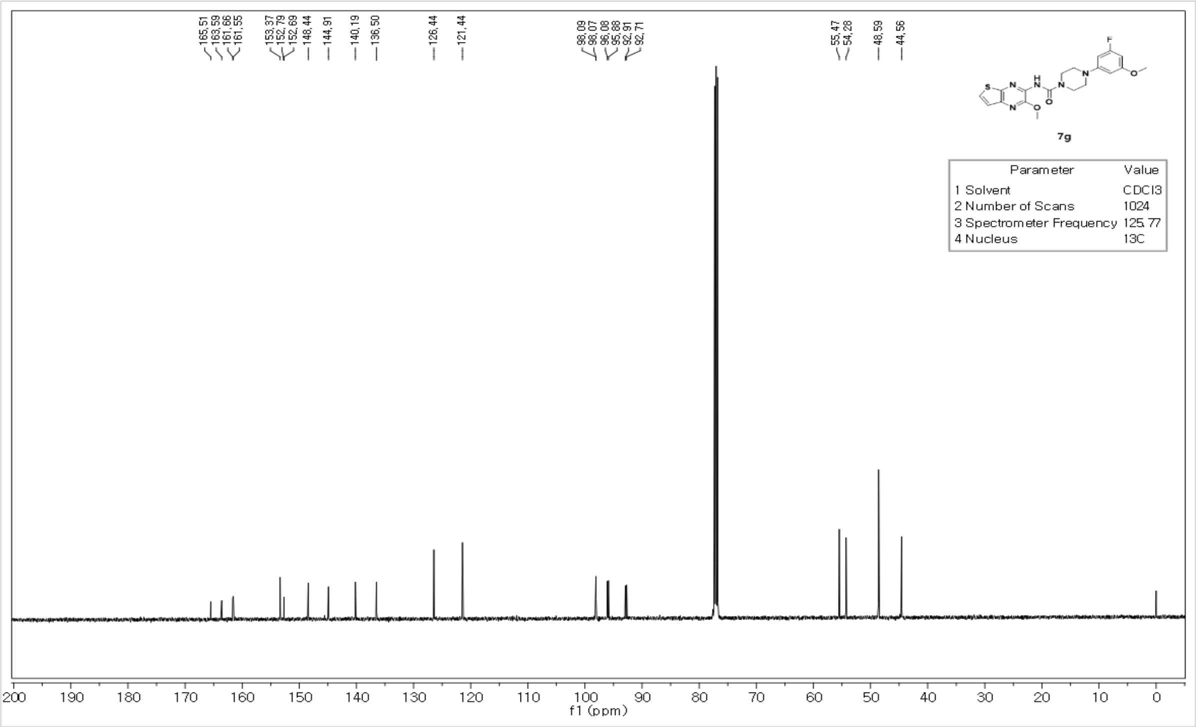

^{13}C-NMR-7g

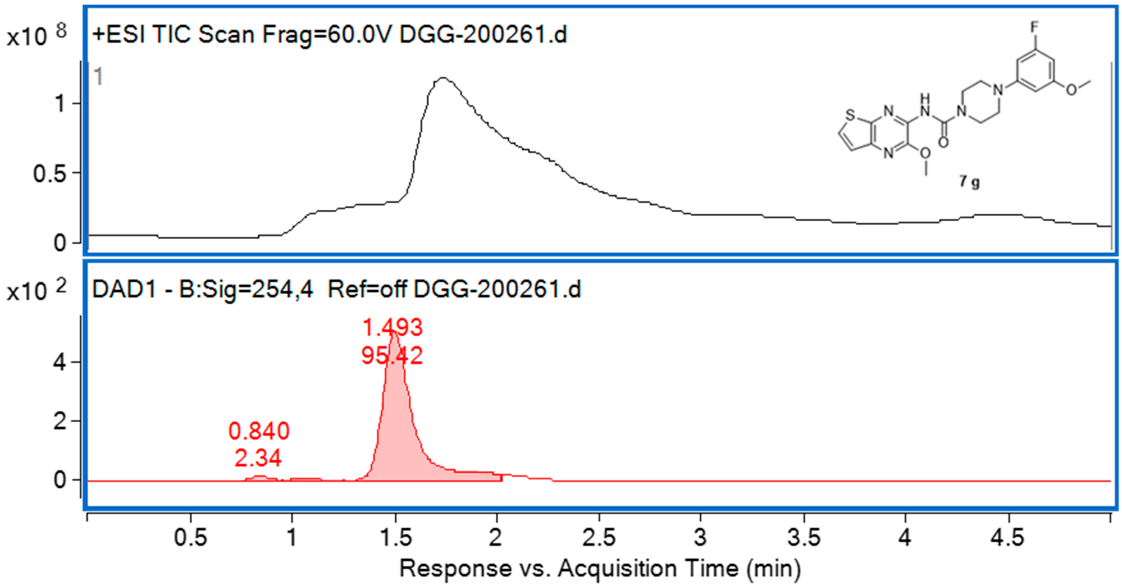

x10 8
+ESI TIC Scan Frag=60.0V DGG-200261.d
7g
x10 2
DAD1 - B:Sig=254,4 Ref=off DGG-200261.d
1.493
95.42
0.840
2.34
Response vs. Acquisition Time (min)

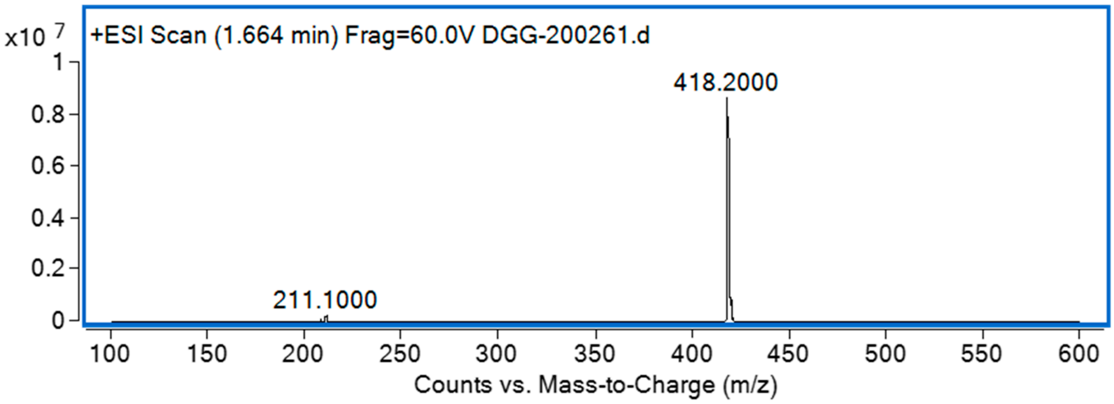

x10 7
+ESI Scan (1.664 min) Frag=60.0V DGG-200261.d
418.2000
211.1000
Counts vs. Mass-to-Charge (m/z)

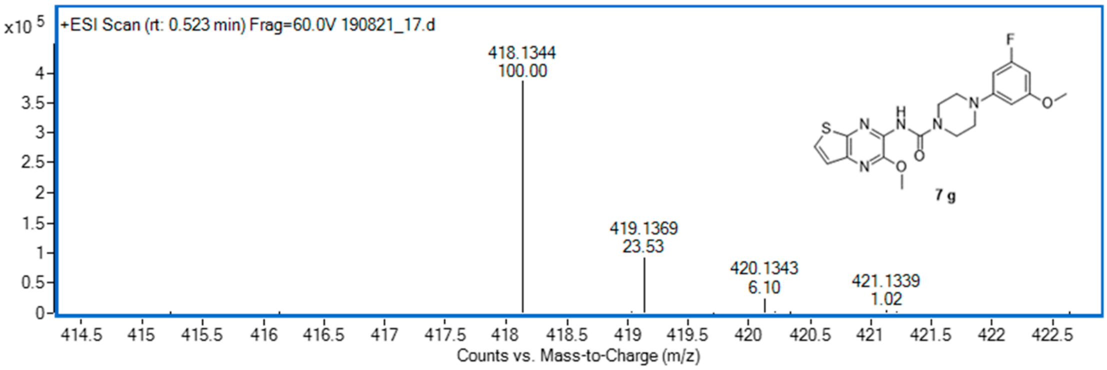

x10 5
+ESI Scan (rt: 0.523 min) Frag=60.0V 190821_17.d
418.1344
100.00
419.1369
23.53
420.1343
6.10
421.1339
1.02
7g
Counts vs. Mass-to-Charge (m/z)

LC/MS, HR/MS-7g

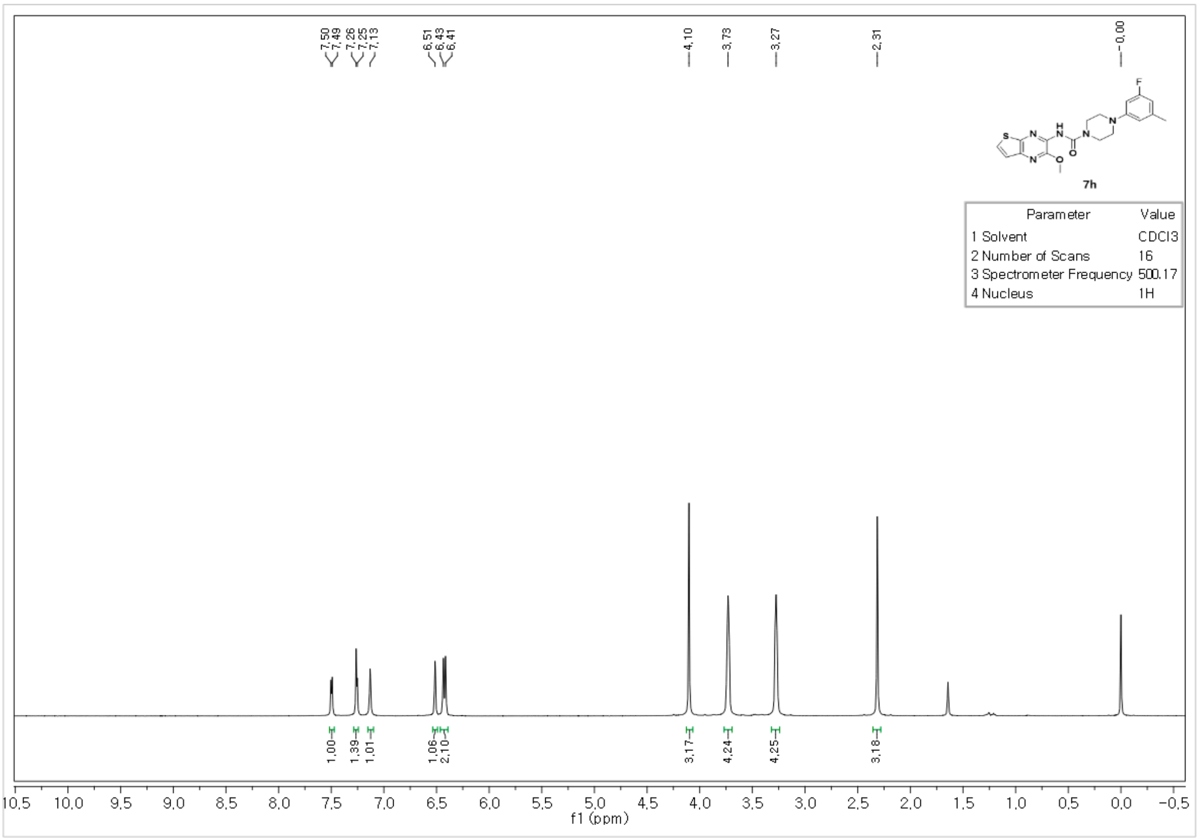

^{1}H-NMR-7h

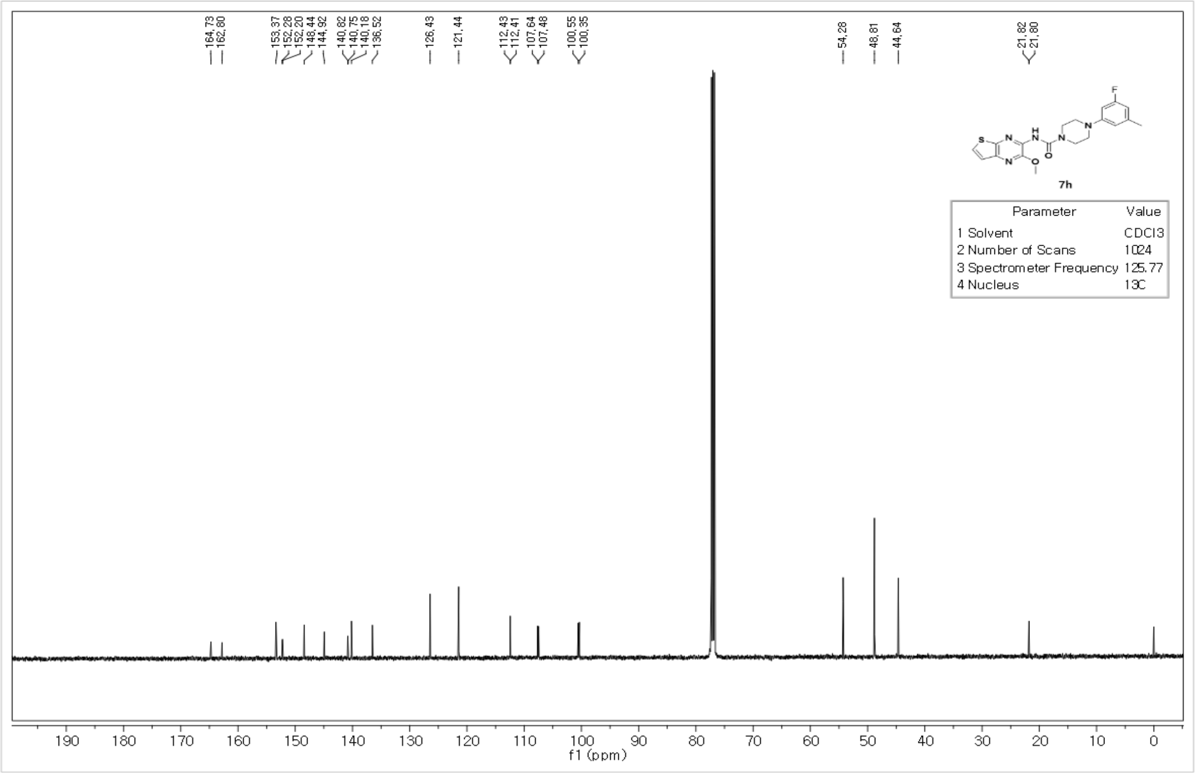

^{13}C-NMR-7h

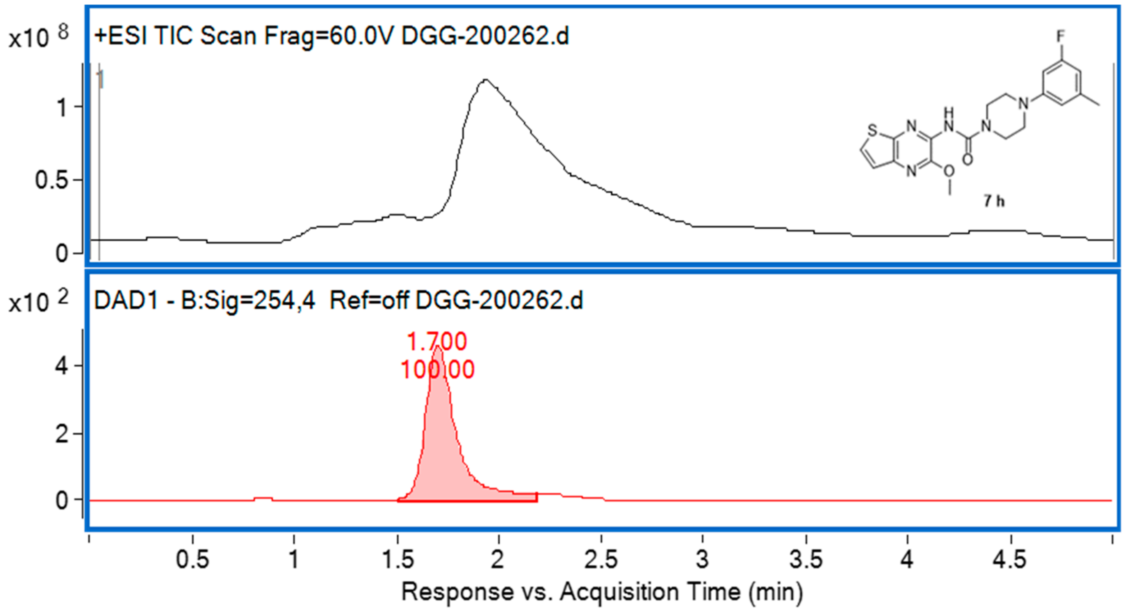
x10 8
+ESI TIC Scan Frag=60.0V DGG-200262.d
7 h
x10 2
DAD1 - B:Sig=254,4 Ref=off DGG-200262.d
1.700
100.00
Response vs. Acquisition Time (min)

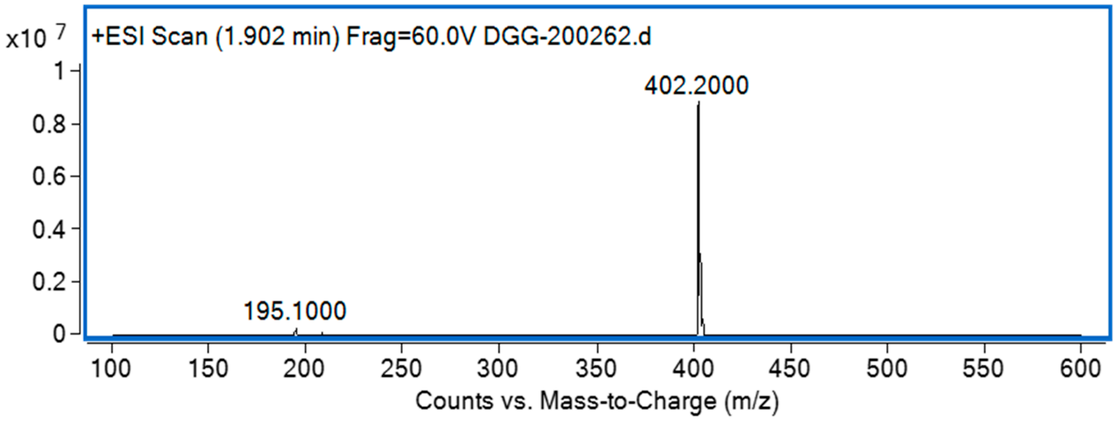
x10 7
+ESI Scan (1.902 min) Frag=60.0V DGG-200262.d
402.2000
195.1000
Counts vs. Mass-to-Charge (m/z)

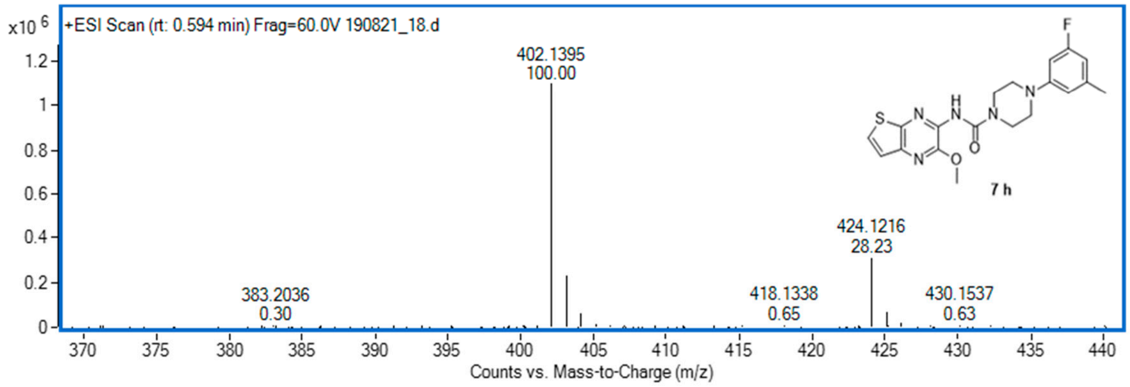
x10 6
+ESI Scan (rt: 0.594 min) Frag=60.0V 190821_18.d
402.1395
100.00
7 h
424.1216
28.23
383.2036
0.30
418.1338
0.65
430.1537
0.63
Counts vs. Mass-to-Charge (m/z)

LC/MS, HR/MS-7h

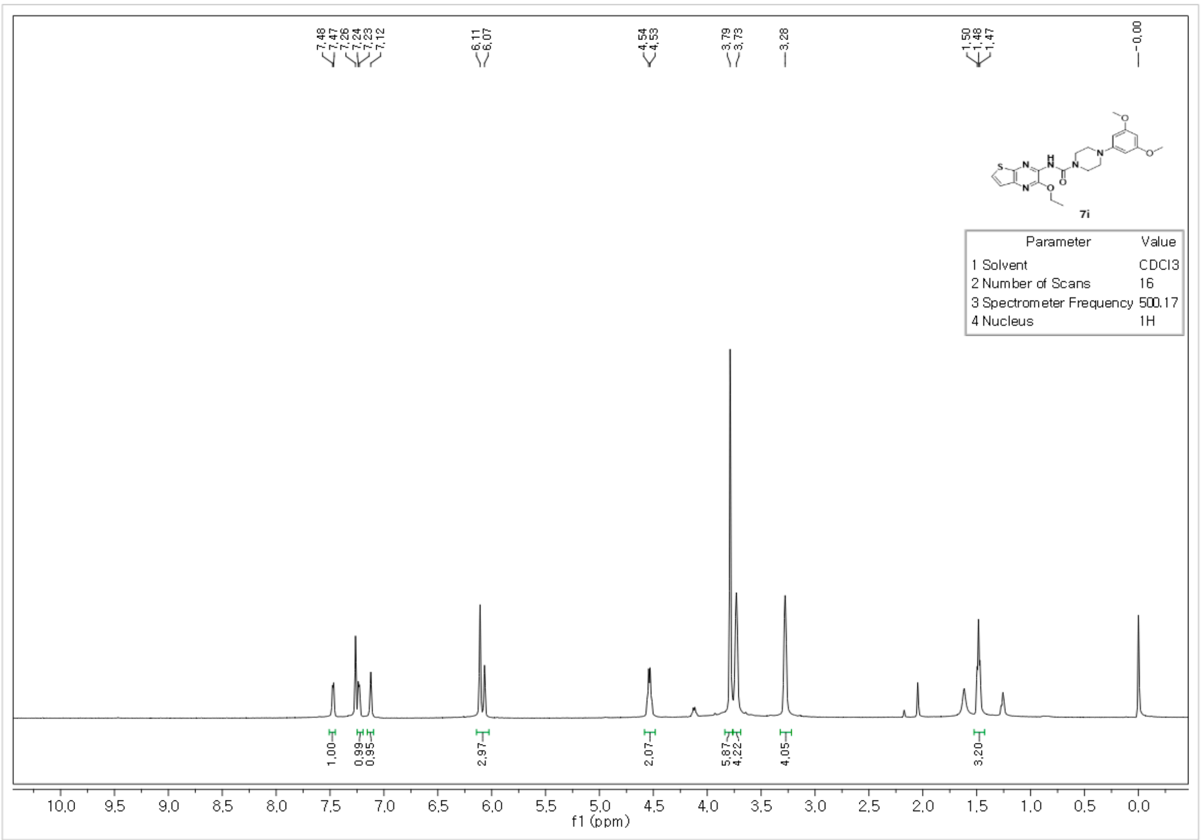

^{1}H-NMR-7i

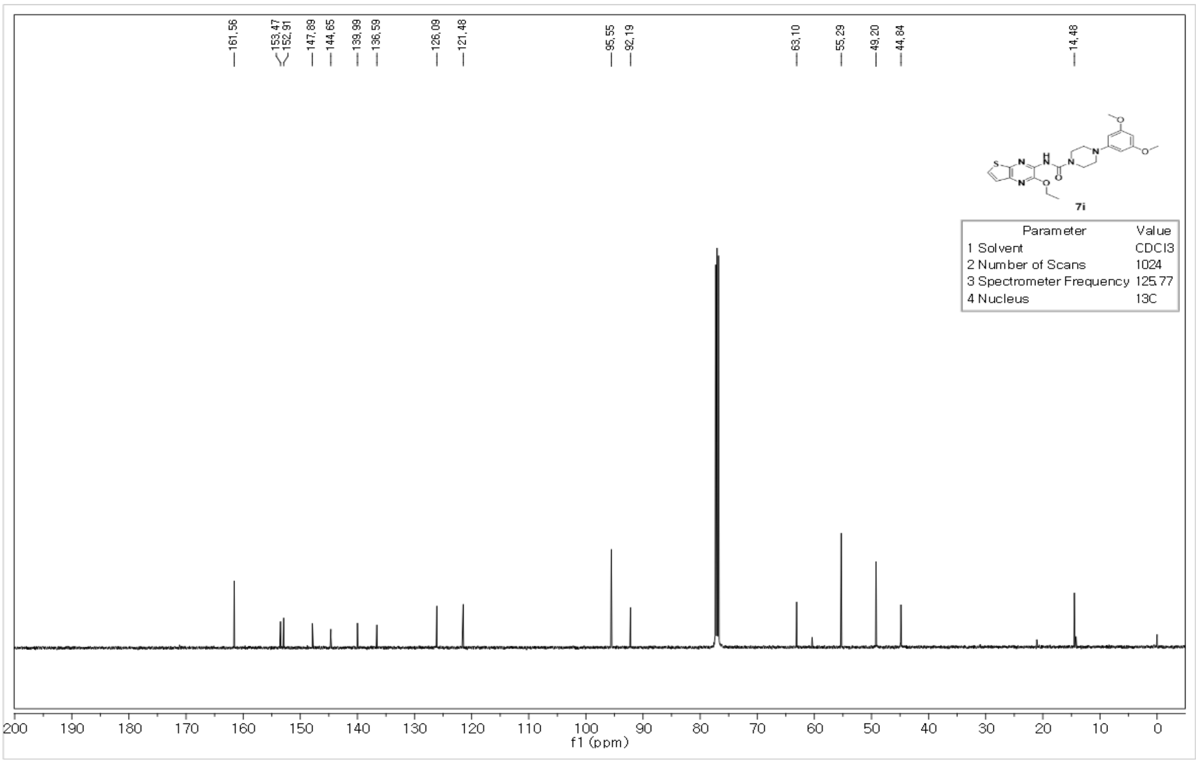

^{13}C-NMR-7i

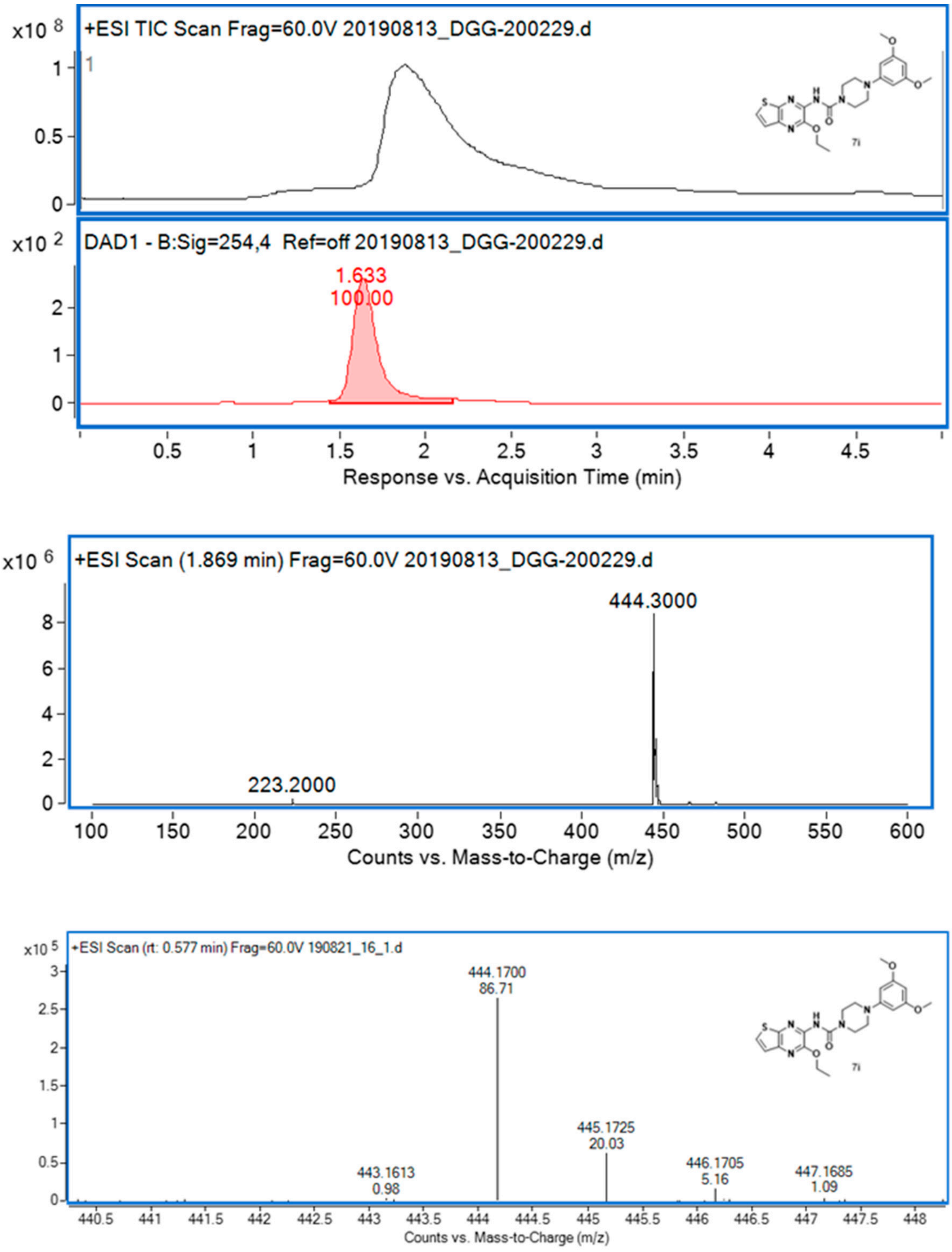
x10 8
+ESI TIC Scan Frag=60.0V 20190813_DGG-200229.d
7i
x10 2
DAD1 - B:Sig=254,4 Ref=off 20190813_DGG-200229.d
1.633
100.00
Response vs. Acquisition Time (min)
x10 6
+ESI Scan (1.869 min) Frag=60.0V 20190813_DGG-200229.d
444.3000
223.2000
Counts vs. Mass-to-Charge (m/z)
x10 5
+ESI Scan (rt: 0.577 min) Frag=60.0V 190821_16_1.d
444.1700
86.71
445.1725
20.03
443.1613
0.98
446.1705
5.16
447.1685
1.09
7i
Counts vs. Mass-to-Charge (m/z)

LC/MS, HR/MS-7i

2. Figures S1 and S2.

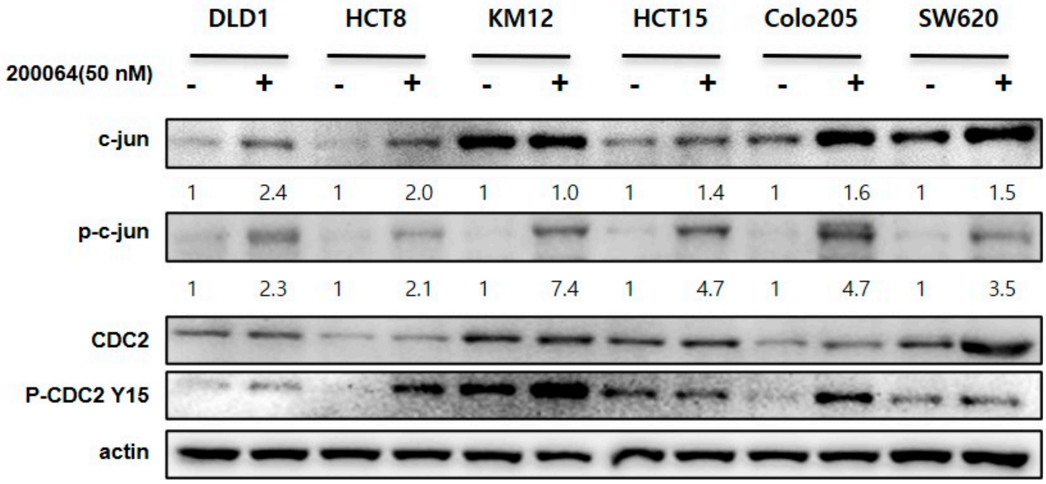

Figure S1. DGG200064 induced cell cycle arrest on colon cancer cells.

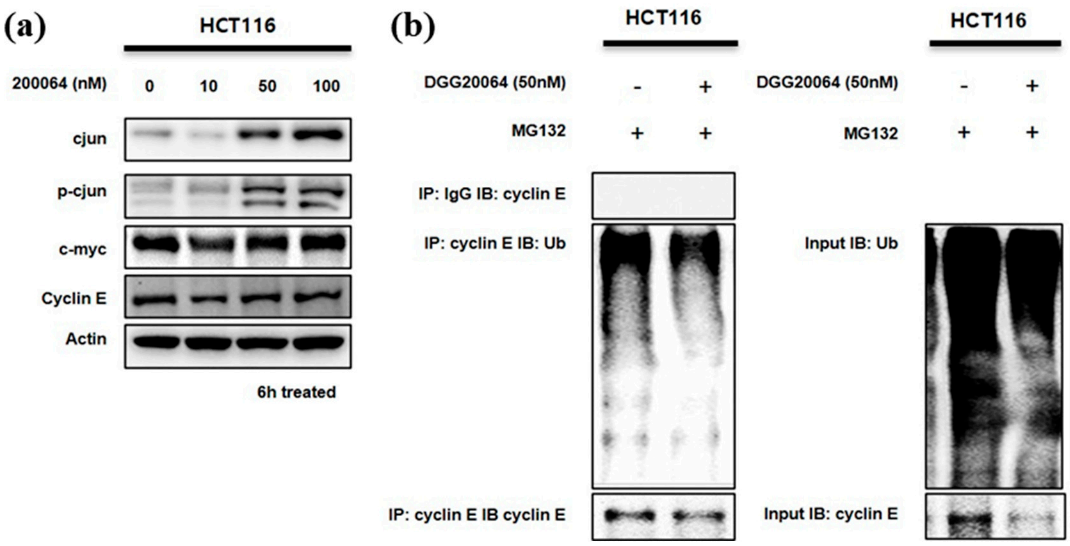

Figure S2. (a) **DGG200064** dose-dependently induces c-Jun on HCT116 cells; (b) **DGG200064** does not affect the ubiquitination of cyclin E.

3. Raw Biological Data

DLD1_DMSO_6h

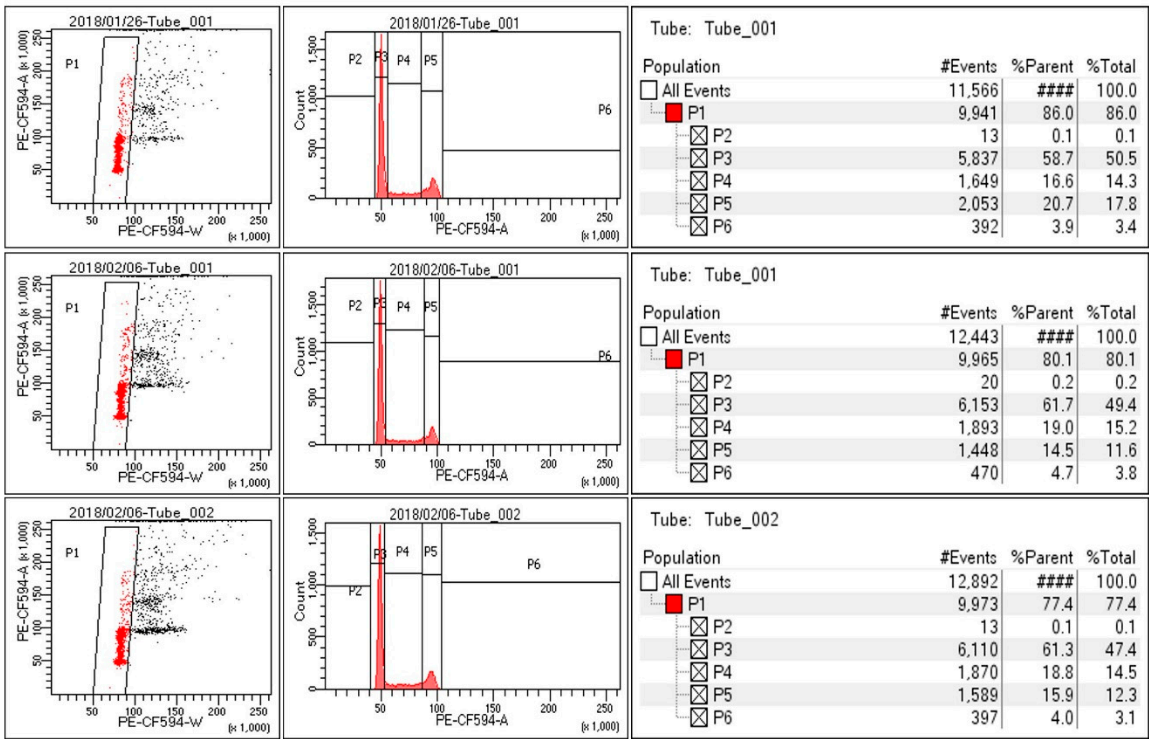

Tube: Tube_001

Population	#Events	%Parent	%Total
All Events	11,566	####	100.0
P1	9,941	86.0	86.0
P2	13	0.1	0.1
P3	5,837	58.7	50.5
P4	1,649	16.6	14.3
P5	2,053	20.7	17.8
P6	392	3.9	3.4

Tube: Tube_001

Population	#Events	%Parent	%Total
All Events	12,443	####	100.0
P1	9,965	80.1	80.1
P2	20	0.2	0.2
P3	6,153	61.7	49.4
P4	1,893	19.0	15.2
P5	1,448	14.5	11.6
P6	470	4.7	3.8

Tube: Tube_002

Population	#Events	%Parent	%Total
All Events	12,892	####	100.0
P1	9,973	77.4	77.4
P2	13	0.1	0.1
P3	6,110	61.3	47.4
P4	1,870	18.8	14.5
P5	1,589	15.9	12.3
P6	397	4.0	3.1

DLD1_DGG20064_50nM_6h

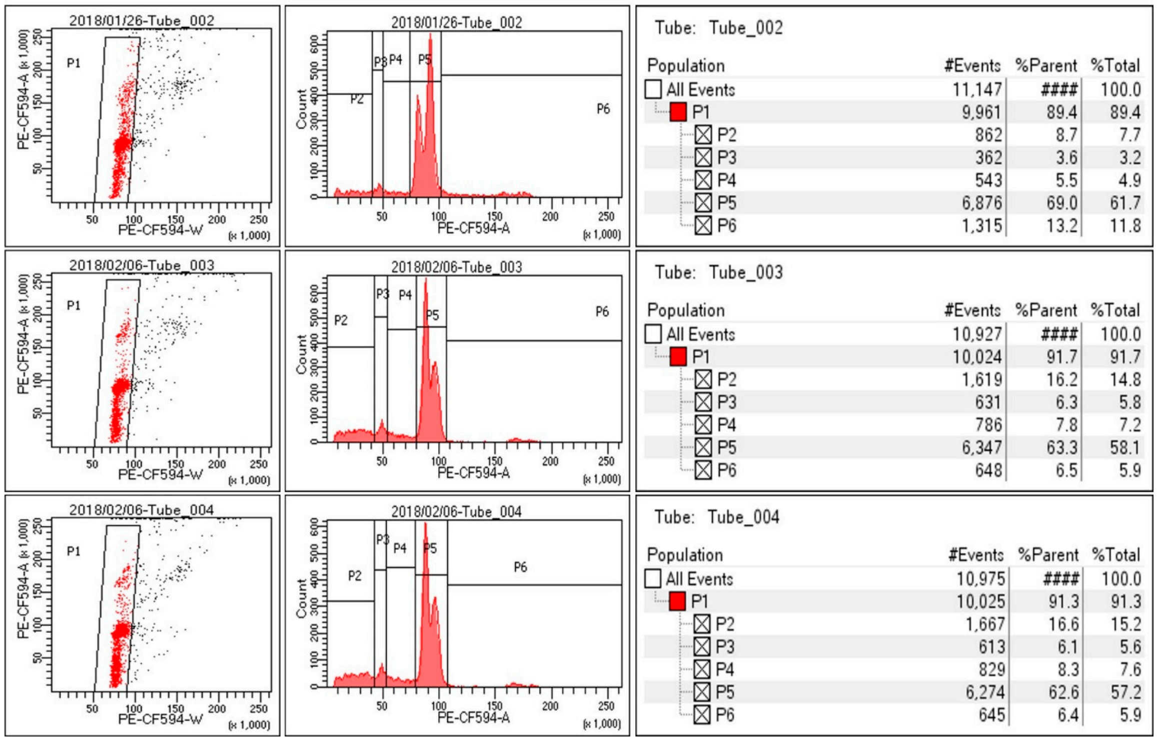

Tube: Tube_002

Population	#Events	%Parent	%Total
All Events	11,147	####	100.0
P1	9,961	89.4	89.4
P2	862	8.7	7.7
P3	362	3.6	3.2
P4	543	5.5	4.9
P5	6,876	69.0	61.7
P6	1,315	13.2	11.8

Tube: Tube_003

Population	#Events	%Parent	%Total
All Events	10,927	####	100.0
P1	10,024	91.7	91.7
P2	1,619	16.2	14.8
P3	631	6.3	5.8
P4	786	7.8	7.2
P5	6,347	63.3	58.1
P6	648	6.5	5.9

Tube: Tube_004

Population	#Events	%Parent	%Total
All Events	10,975	####	100.0
P1	10,025	91.3	91.3
P2	1,667	16.6	15.2
P3	613	6.1	5.6
P4	829	8.3	7.6
P5	6,274	62.6	57.2
P6	645	6.4	5.9

HCT8_DMSO_6h

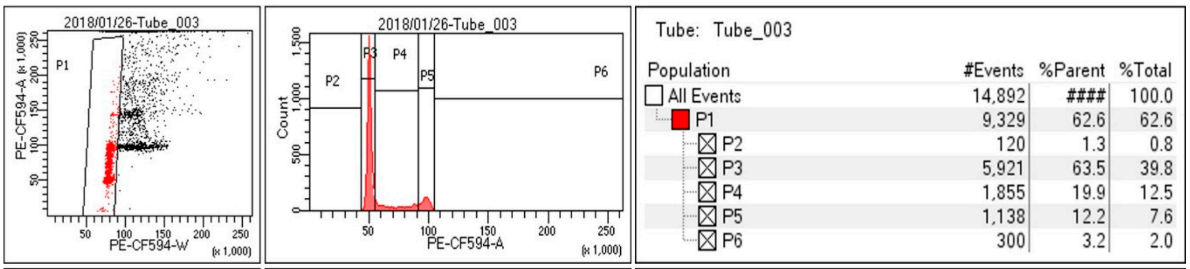

Tube: Tube_003

Population	#Events	%Parent	%Total
All Events	14,892	####	100.0
P1	9,329	62.6	62.6
P2	120	1.3	0.8
P3	5,921	63.5	39.8
P4	1,855	19.9	12.5
P5	1,138	12.2	7.6
P6	300	3.2	2.0

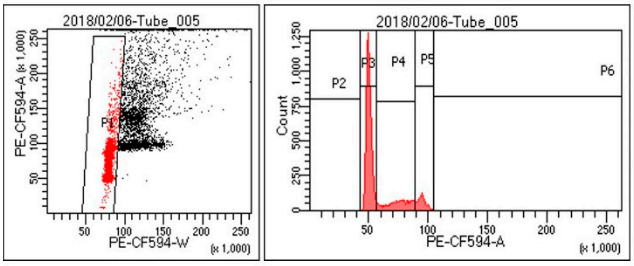

Tube: Tube_005

Population	#Events	%Parent	%Total
All Events	12,545	####	100.0
P1	9,787	78.0	78.0
P2	30	0.3	0.2
P3	6,423	65.6	51.2
P4	2,099	21.4	16.7
P5	942	9.6	7.5
P6	274	2.8	2.2

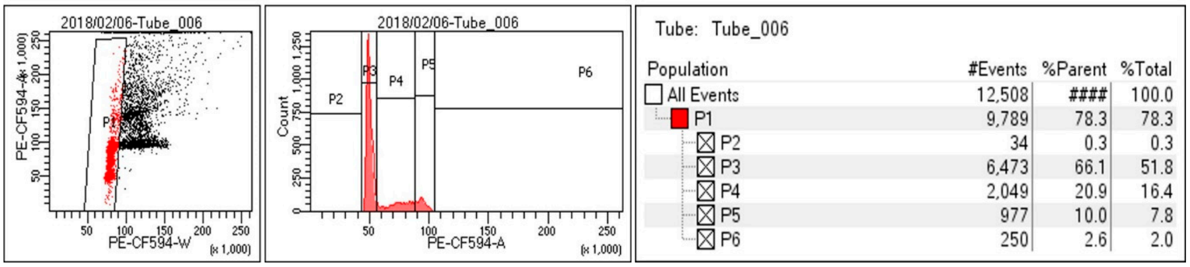

Tube: Tube_006

Population	#Events	%Parent	%Total
All Events	12,508	####	100.0
P1	9,789	78.3	78.3
P2	34	0.3	0.3
P3	6,473	66.1	51.8
P4	2,049	20.9	16.4
P5	977	10.0	7.8
P6	250	2.6	2.0

HCT8_DGG20064_50nM_6h

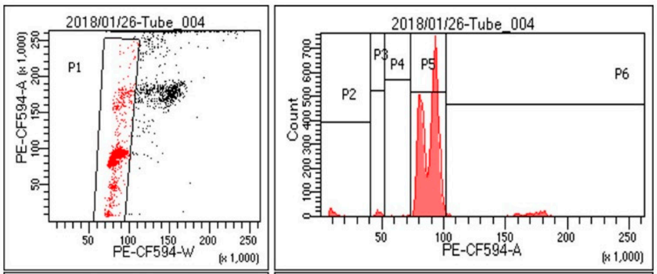

Tube: Tube_004

Population	#Events	%Parent	%Total
All Events	13,531	####	100.0
P1	10,111	74.7	74.7
P2	339	3.4	2.5
P3	183	1.8	1.4
P4	122	1.2	0.9
P5	8,664	85.7	64.0
P6	803	7.9	5.9

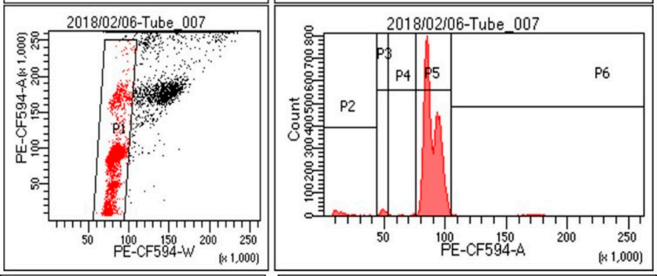

Tube: Tube_007

Population	#Events	%Parent	%Total
All Events	12,204	####	100.0
P1	10,088	82.7	82.7
P2	468	4.6	3.8
P3	208	2.1	1.7
P4	183	1.8	1.5
P5	8,767	86.9	71.8
P6	459	4.5	3.8

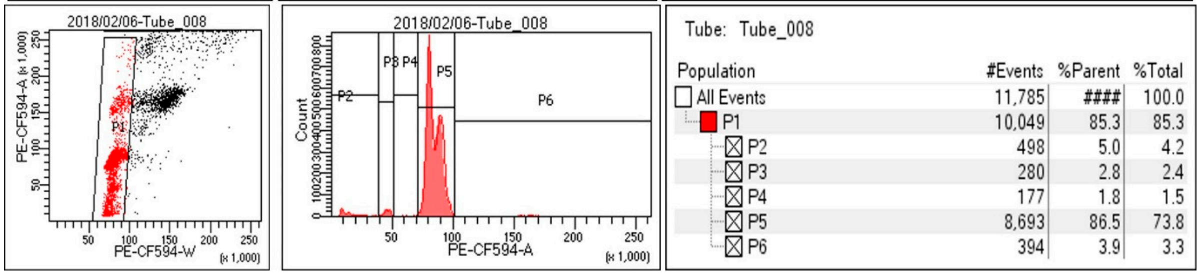

Tube: Tube_008

Population	#Events	%Parent	%Total
All Events	11,785	####	100.0
P1	10,049	85.3	85.3
P2	498	5.0	4.2
P3	280	2.8	2.4
P4	177	1.8	1.5
P5	8,693	86.5	73.8
P6	394	3.9	3.3

KM12_DMSO_6h

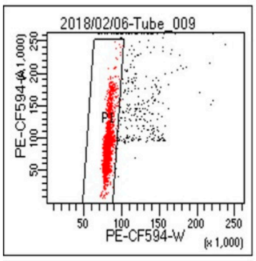

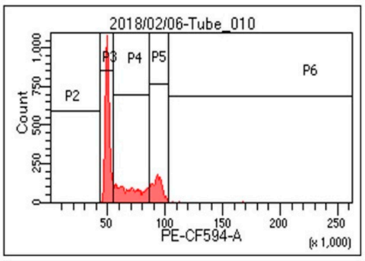

Tube: Tube_009

Population	#Events	%Parent	%Total
All Events	10,299	####	100.0
P1	9,986	97.0	97.0
P2	119	1.2	1.2
P3	4,563	45.7	44.3
P4	3,015	30.2	29.3
P5	1,774	17.8	17.2
P6	518	5.2	5.0

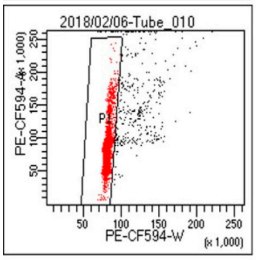

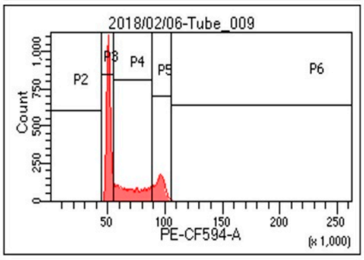

Tube: Tube_010

Population	#Events	%Parent	%Total
All Events	10,283	####	100.0
P1	9,952	96.8	96.8
P2	105	1.1	1.0
P3	4,618	46.4	44.9
P4	2,816	28.3	27.4
P5	1,830	18.4	17.8
P6	550	5.5	5.3

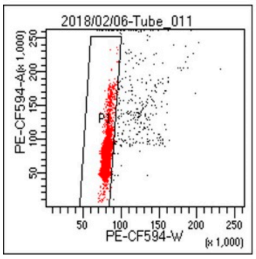

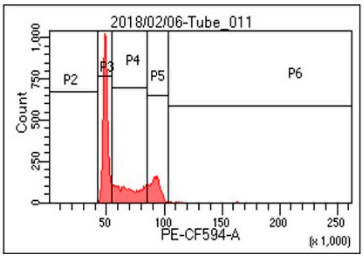

Tube: Tube_011

Population	#Events	%Parent	%Total
All Events	10,280	####	100.0
P1	9,959	96.9	96.9
P2	104	1.0	1.0
P3	4,781	48.0	46.5
P4	2,860	28.7	27.8
P5	1,708	17.2	16.6
P6	518	5.2	5.0

KM12_DGG20064_50nM_6h

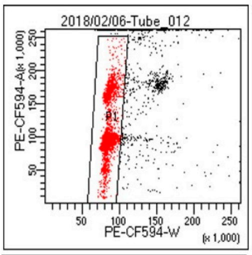

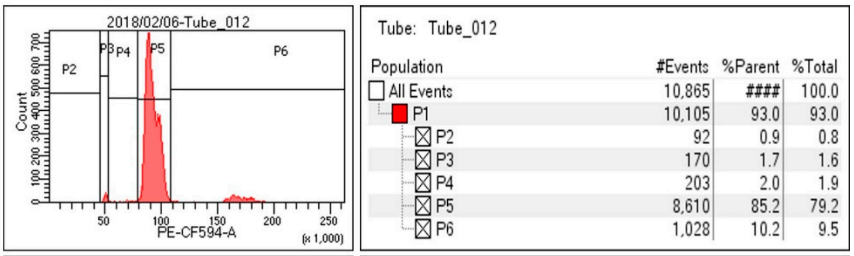

Tube: Tube_012

Population	#Events	%Parent	%Total
All Events	10,865	####	100.0
P1	10,105	93.0	93.0
P2	92	0.9	0.8
P3	170	1.7	1.6
P4	203	2.0	1.9
P5	8,610	85.2	79.2
P6	1,028	10.2	9.5

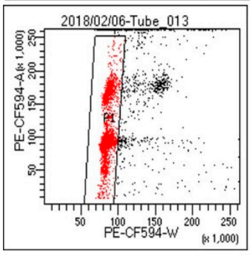

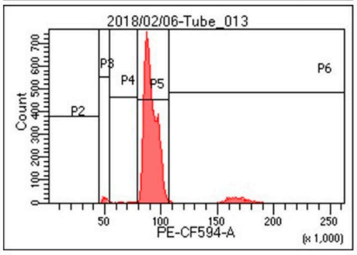

Tube: Tube_013

Population	#Events	%Parent	%Total
All Events	10,921	####	100.0
P1	10,067	92.2	92.2
P2	93	0.9	0.9
P3	165	1.6	1.5
P4	208	2.1	1.9
P5	8,584	85.3	78.6
P6	1,017	10.1	9.3

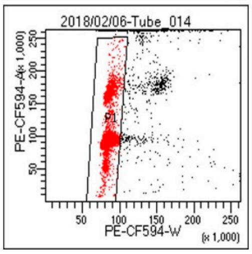

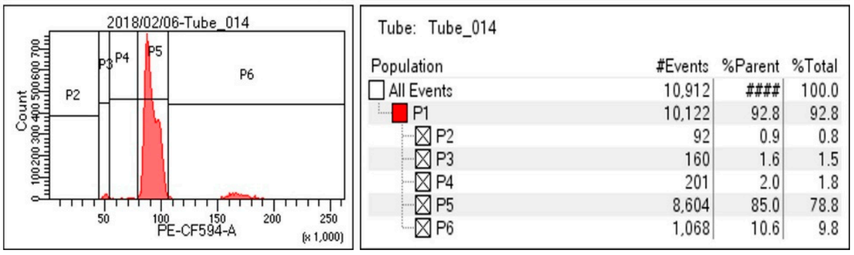

Tube: Tube_014

Population	#Events	%Parent	%Total
All Events	10,912	####	100.0
P1	10,122	92.8	92.8
P2	92	0.9	0.8
P3	160	1.6	1.5
P4	201	2.0	1.8
P5	8,604	85.0	78.8
P6	1,068	10.6	9.8

HCT15_DMSO_6h

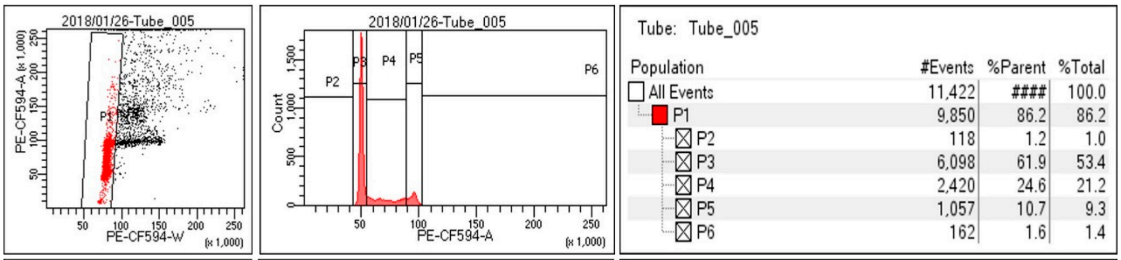

Tube: Tube_005

Population	#Events	%Parent	%Total
All Events	11,422	####	100.0
P1	9,850	86.2	86.2
P2	118	1.2	1.0
P3	6,098	61.9	53.4
P4	2,420	24.6	21.2
P5	1,057	10.7	9.3
P6	162	1.6	1.4

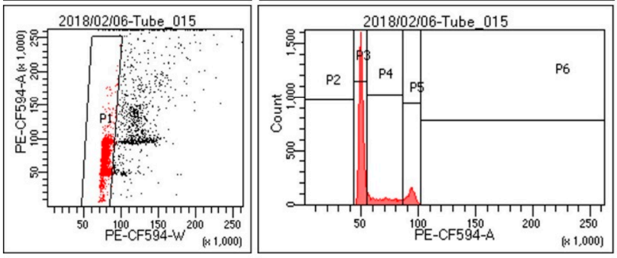

Tube: Tube_015

Population	#Events	%Parent	%Total
All Events	10,928	####	100.0
P1	9,934	90.9	90.9
P2	115	1.2	1.1
P3	6,520	65.6	59.7
P4	1,943	19.6	17.8
P5	1,239	12.5	11.3
P6	92	0.9	0.8

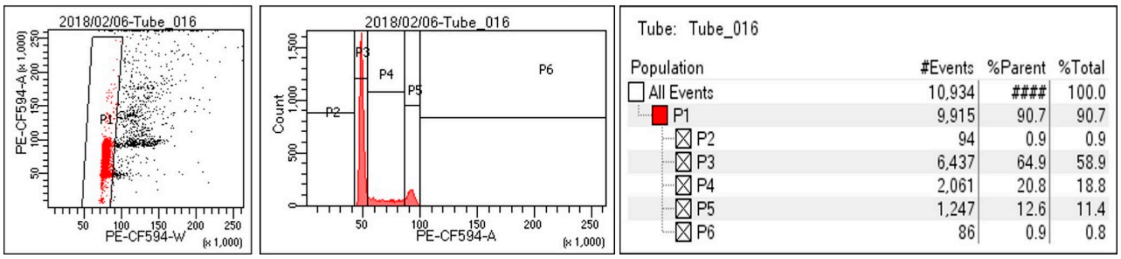

Tube: Tube_016

Population	#Events	%Parent	%Total
All Events	10,934	####	100.0
P1	9,915	90.7	90.7
P2	94	0.9	0.9
P3	6,437	64.9	58.9
P4	2,061	20.8	18.8
P5	1,247	12.6	11.4
P6	86	0.9	0.8

HCT15_DGG20064_50nM_6h

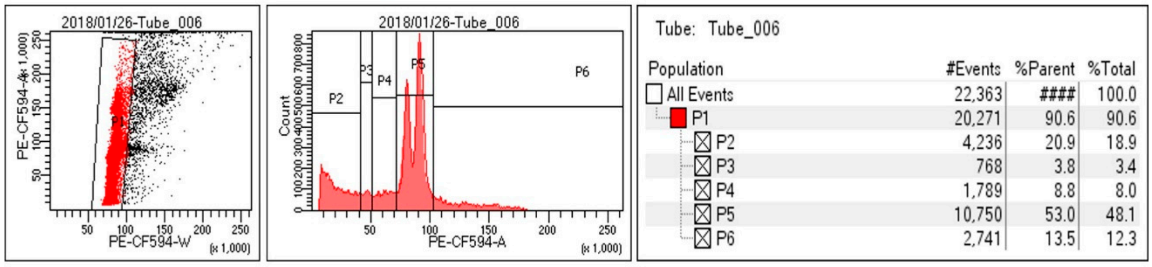

Tube: Tube_006

Population	#Events	%Parent	%Total
All Events	22,363	####	100.0
P1	20,271	90.6	90.6
P2	4,236	20.9	18.9
P3	768	3.8	3.4
P4	1,789	8.8	8.0
P5	10,750	53.0	48.1
P6	2,741	13.5	12.3

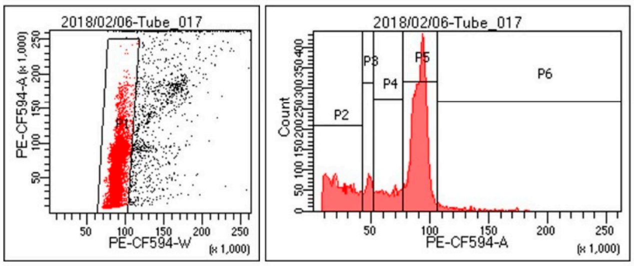

Tube: Tube_017

Population	#Events	%Parent	%Total
All Events	11,524	####	100.0
P1	10,329	89.6	89.6
P2	2,322	22.5	20.1
P3	667	6.5	5.8
P4	1,235	12.0	10.7
P5	5,496	53.2	47.7
P6	622	6.0	5.4

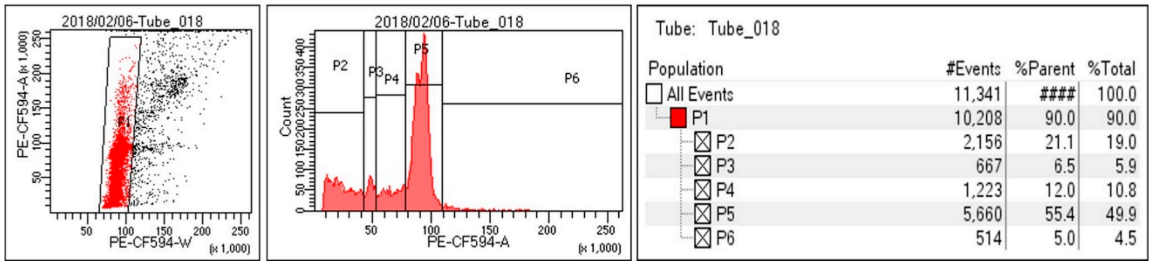

Tube: Tube_018

Population	#Events	%Parent	%Total
All Events	11,341	####	100.0
P1	10,208	90.0	90.0
P2	2,156	21.1	19.0
P3	667	6.5	5.9
P4	1,223	12.0	10.8
P5	5,660	55.4	49.9
P6	514	5.0	4.5

HT29_DGG20064_50nM_6h

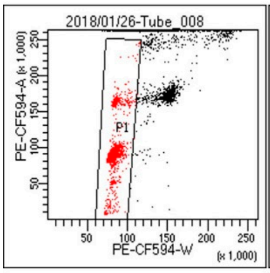

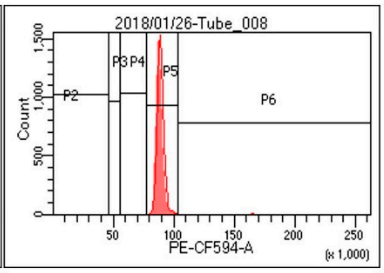

Tube: Tube_008

Population	#Events	%Parent	%Total
All Events	11,461	####	100.0
P1	10,044	87.6	87.6
P2	88	0.9	0.8
P3	57	0.6	0.5
P4	41	0.4	0.4
P5	9,587	95.5	83.6
P6	272	2.7	2.4

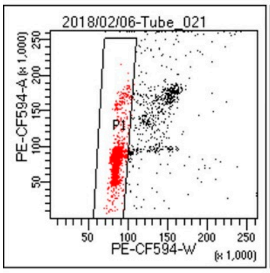

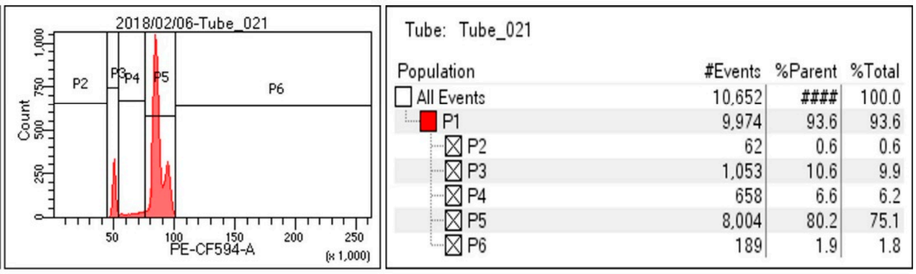

Tube: Tube_021

Population	#Events	%Parent	%Total
All Events	10,652	####	100.0
P1	9,974	93.6	93.6
P2	62	0.6	0.6
P3	1,053	10.6	9.9
P4	658	6.6	6.2
P5	8,004	80.2	75.1
P6	189	1.9	1.8

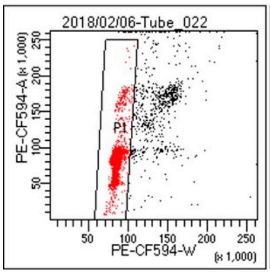

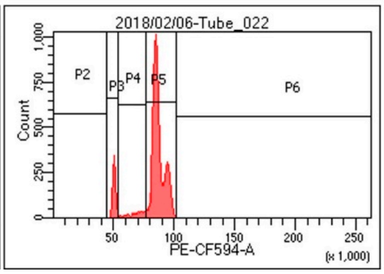

Tube: Tube_022

Population	#Events	%Parent	%Total
All Events	10,692	####	100.0
P1	9,985	93.4	93.4
P2	72	0.7	0.7
P3	1,086	10.9	10.2
P4	680	6.8	6.4
P5	7,929	79.4	74.2
P6	206	2.1	1.9

HT29_DGG20064_50nM_6h

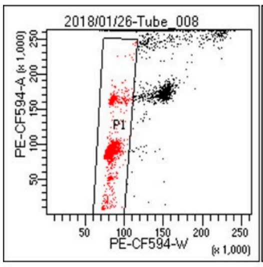

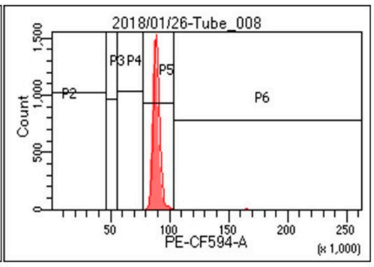

Tube: Tube_008

Population	#Events	%Parent	%Total
All Events	11,461	####	100.0
P1	10,044	87.6	87.6
P2	88	0.9	0.8
P3	57	0.6	0.5
P4	41	0.4	0.4
P5	9,587	95.5	83.6
P6	272	2.7	2.4

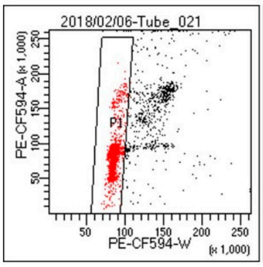

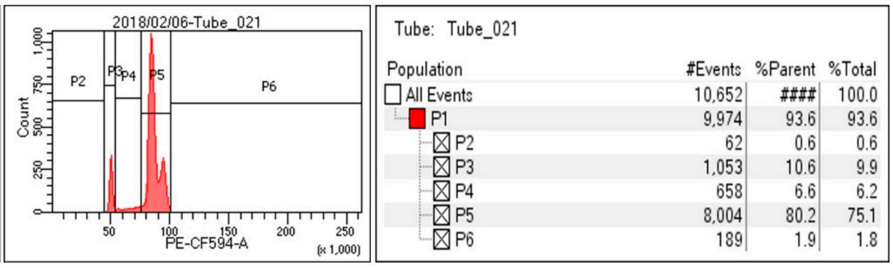

Tube: Tube_021

Population	#Events	%Parent	%Total
All Events	10,652	####	100.0
P1	9,974	93.6	93.6
P2	62	0.6	0.6
P3	1,053	10.6	9.9
P4	658	6.6	6.2
P5	8,004	80.2	75.1
P6	189	1.9	1.8

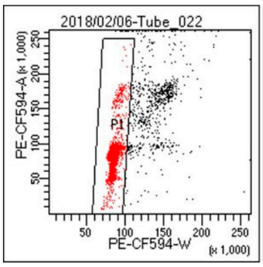

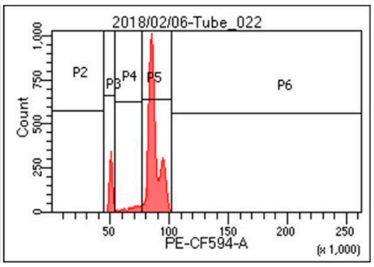

Tube: Tube_022

Population	#Events	%Parent	%Total
All Events	10,692	####	100.0
P1	9,985	93.4	93.4
P2	72	0.7	0.7
P3	1,086	10.9	10.2
P4	680	6.8	6.4
P5	7,929	79.4	74.2
P6	206	2.1	1.9

COLO-205_DMSO_6h

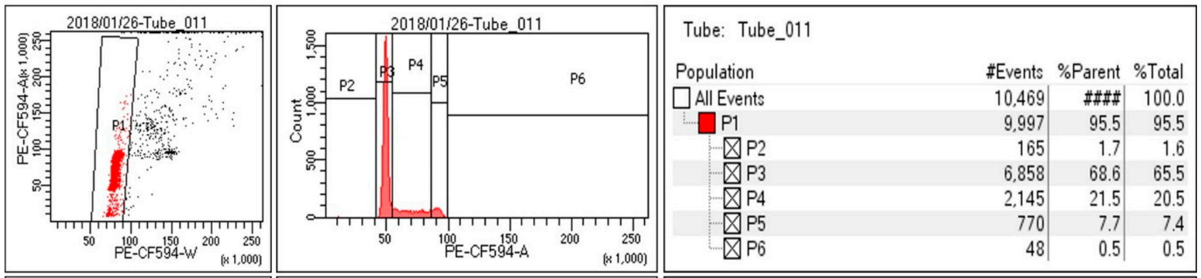

Tube: Tube_011

Population	#Events	%Parent	%Total
All Events	10,469	####	100.0
P1	9,997	95.5	95.5
P2	165	1.7	1.6
P3	6,858	68.6	65.5
P4	2,145	21.5	20.5
P5	770	7.7	7.4
P6	48	0.5	0.5

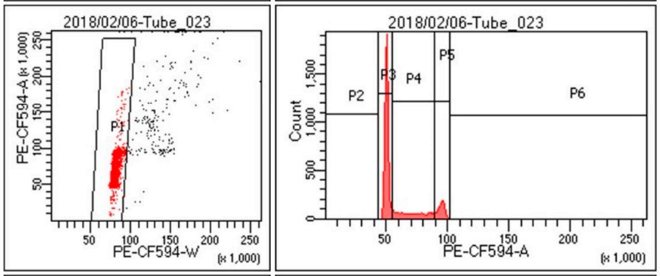

Tube: Tube_023

Population	#Events	%Parent	%Total
All Events	10,209	####	100.0
P1	9,990	97.9	97.9
P2	46	0.5	0.5
P3	6,181	61.9	60.5
P4	2,375	23.8	23.3
P5	1,322	13.2	12.9
P6	52	0.5	0.5

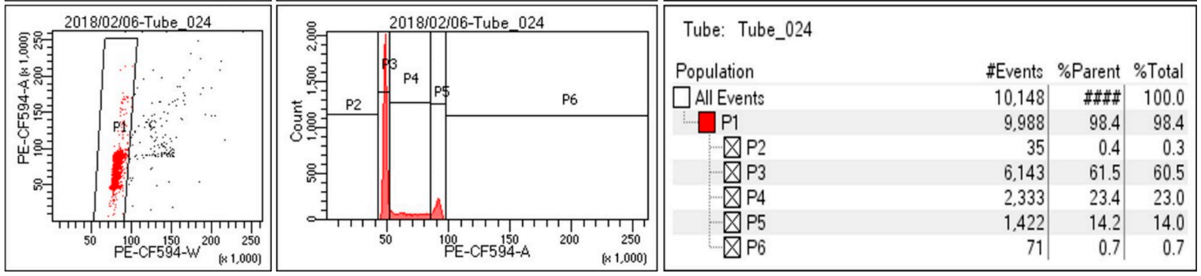

Tube: Tube_024

Population	#Events	%Parent	%Total
All Events	10,148	####	100.0
P1	9,988	98.4	98.4
P2	35	0.4	0.3
P3	6,143	61.5	60.5
P4	2,333	23.4	23.0
P5	1,422	14.2	14.0
P6	71	0.7	0.7

COLO-205_DGG20064_50nM_6h

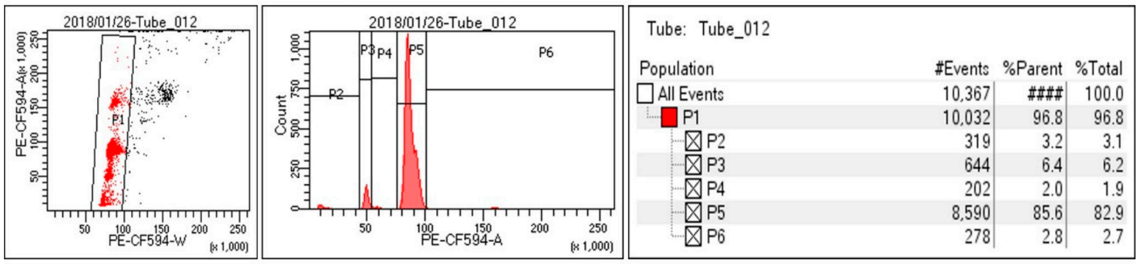

Tube: Tube_012

Population	#Events	%Parent	%Total
All Events	10,367	####	100.0
P1	10,032	96.8	96.8
P2	319	3.2	3.1
P3	644	6.4	6.2
P4	202	2.0	1.9
P5	8,590	85.6	82.9
P6	278	2.8	2.7

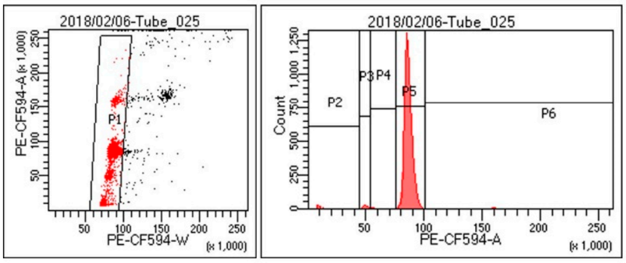

Tube: Tube_025

Population	#Events	%Parent	%Total
All Events	10,317	####	100.0
P1	9,977	96.7	96.7
P2	264	2.6	2.6
P3	217	2.2	2.1
P4	160	1.6	1.6
P5	9,111	91.3	88.3
P6	222	2.2	2.2

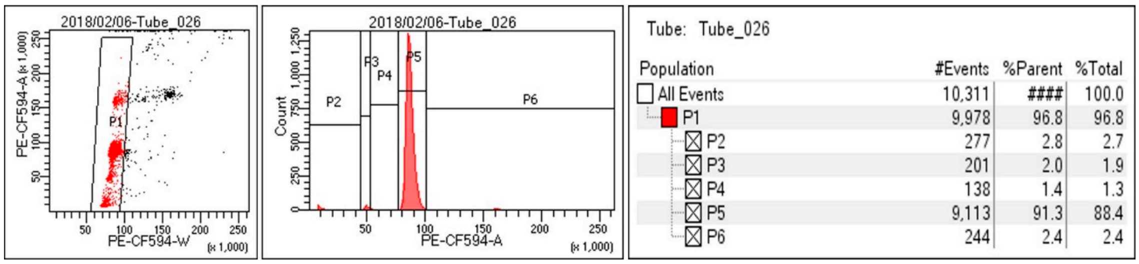

Tube: Tube_026

Population	#Events	%Parent	%Total
All Events	10,311	####	100.0
P1	9,978	96.8	96.8
P2	277	2.8	2.7
P3	201	2.0	1.9
P4	138	1.4	1.3
P5	9,113	91.3	88.4
P6	244	2.4	2.4

SW620_DMSO_6h

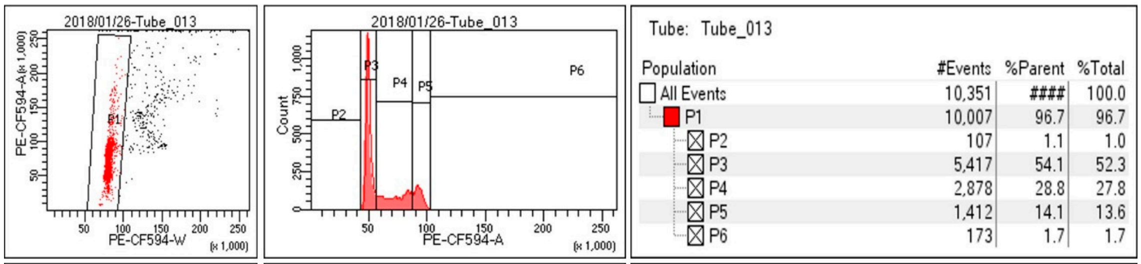

Tube: Tube_013

Population	#Events	%Parent	%Total
All Events	10,351	####	100.0
P1	10,007	96.7	96.7
P2	107	1.1	1.0
P3	5,417	54.1	52.3
P4	2,878	28.8	27.8
P5	1,412	14.1	13.6
P6	173	1.7	1.7

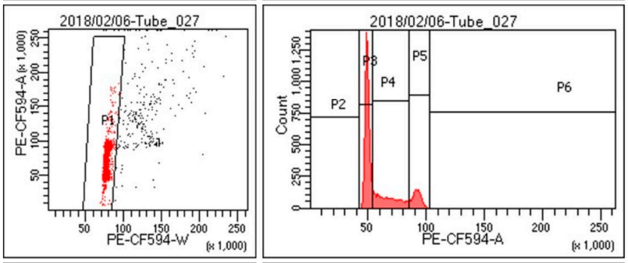

Tube: Tube_027

Population	#Events	%Parent	%Total
All Events	10,224	####	100.0
P1	9,969	97.5	97.5
P2	75	0.8	0.7
P3	5,773	57.9	56.5
P4	2,561	25.7	25.0
P5	1,458	14.6	14.3
P6	71	0.7	0.7

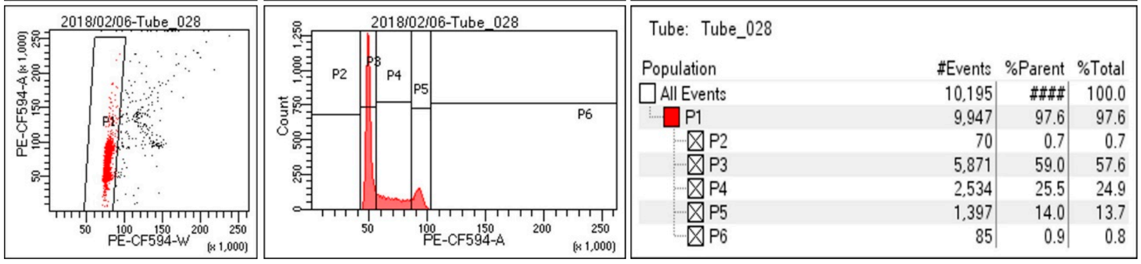

Tube: Tube_028

Population	#Events	%Parent	%Total
All Events	10,195	####	100.0
P1	9,947	97.6	97.6
P2	70	0.7	0.7
P3	5,871	59.0	57.6
P4	2,534	25.5	24.9
P5	1,397	14.0	13.7
P6	85	0.9	0.8

SW620_DGG20064_50nM_6h

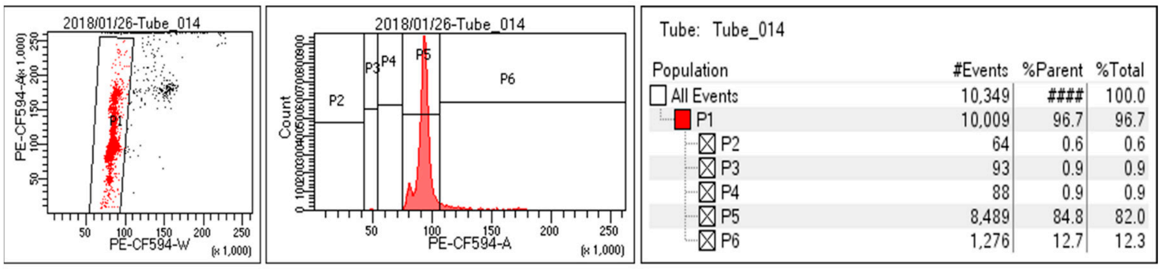

Tube: Tube_014

Population	#Events	%Parent	%Total
All Events	10,349	####	100.0
P1	10,009	96.7	96.7
P2	64	0.6	0.6
P3	93	0.9	0.9
P4	88	0.9	0.9
P5	8,489	84.8	82.0
P6	1,276	12.7	12.3

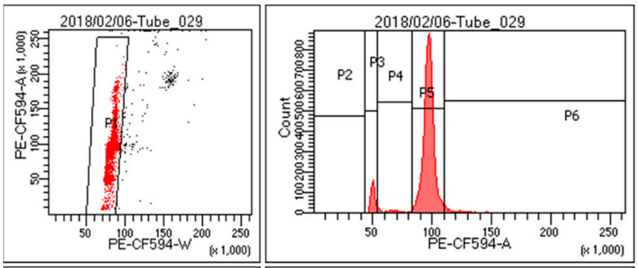

Tube: Tube_029

Population	#Events	%Parent	%Total
All Events	10,139	####	100.0
P1	9,970	98.3	98.3
P2	110	1.1	1.1
P3	744	7.5	7.3
P4	401	4.0	4.0
P5	8,092	81.2	79.8
P6	630	6.3	6.2

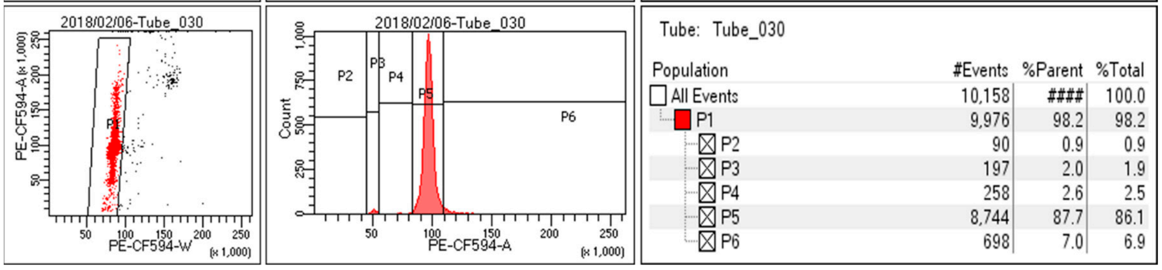

Tube: Tube_030

Population	#Events	%Parent	%Total
All Events	10,158	####	100.0
P1	9,976	98.2	98.2
P2	90	0.9	0.9
P3	197	2.0	1.9
P4	258	2.6	2.5
P5	8,744	87.7	86.1
P6	698	7.0	6.9

Figure 2A HCT116 and DLD1_DGG200064 dose-dependent treatment

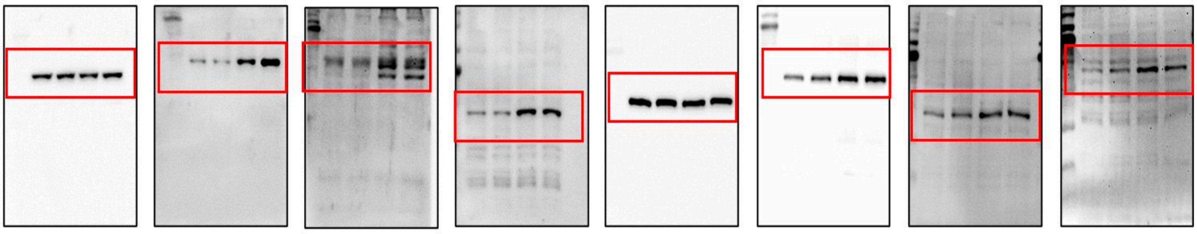

Figure 2C HCT116_c-Jun siRNA

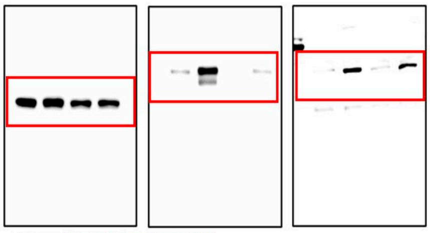

Figure 2D. HCT116_FBW7 siRNA

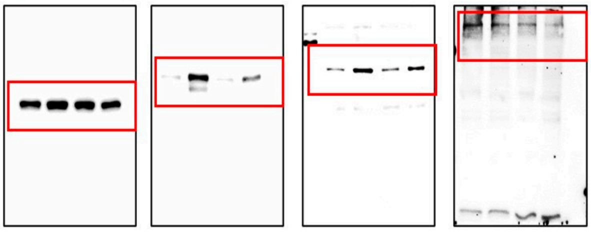

Figure 2C. DLD1_c-Jun siRNA

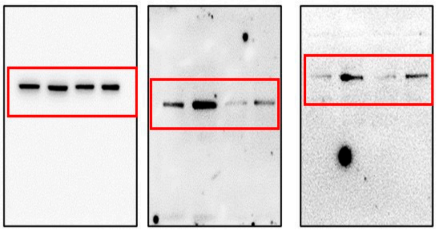

Figure 2D. DLD1_FBW7 siRNA

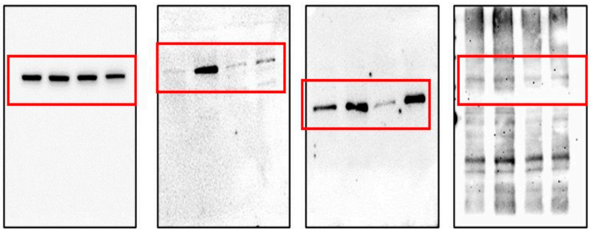

Figure 3.A HCT116

Figure 3.B DLD1

Figure 4.B

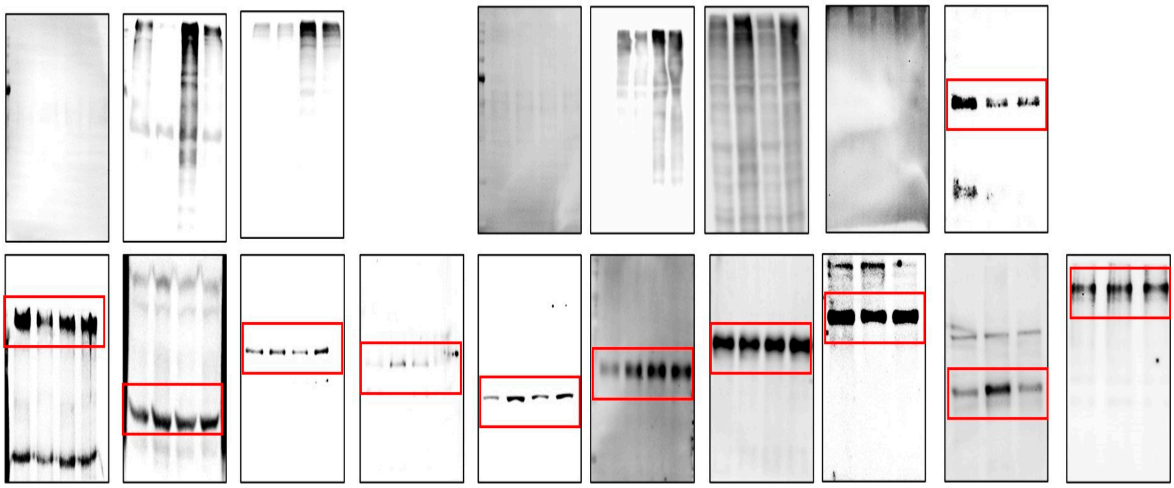

Figure 2A

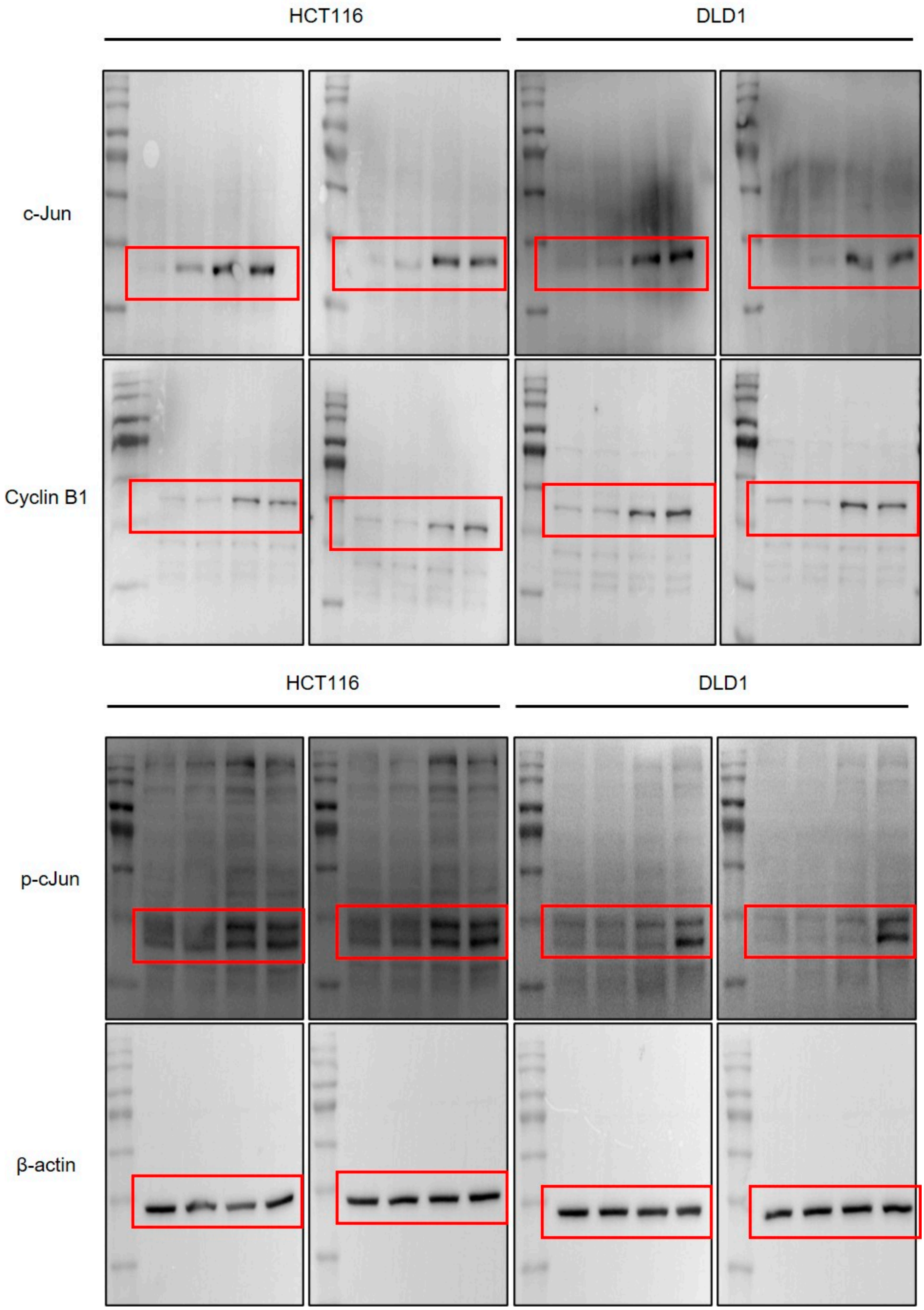

Figure 2B

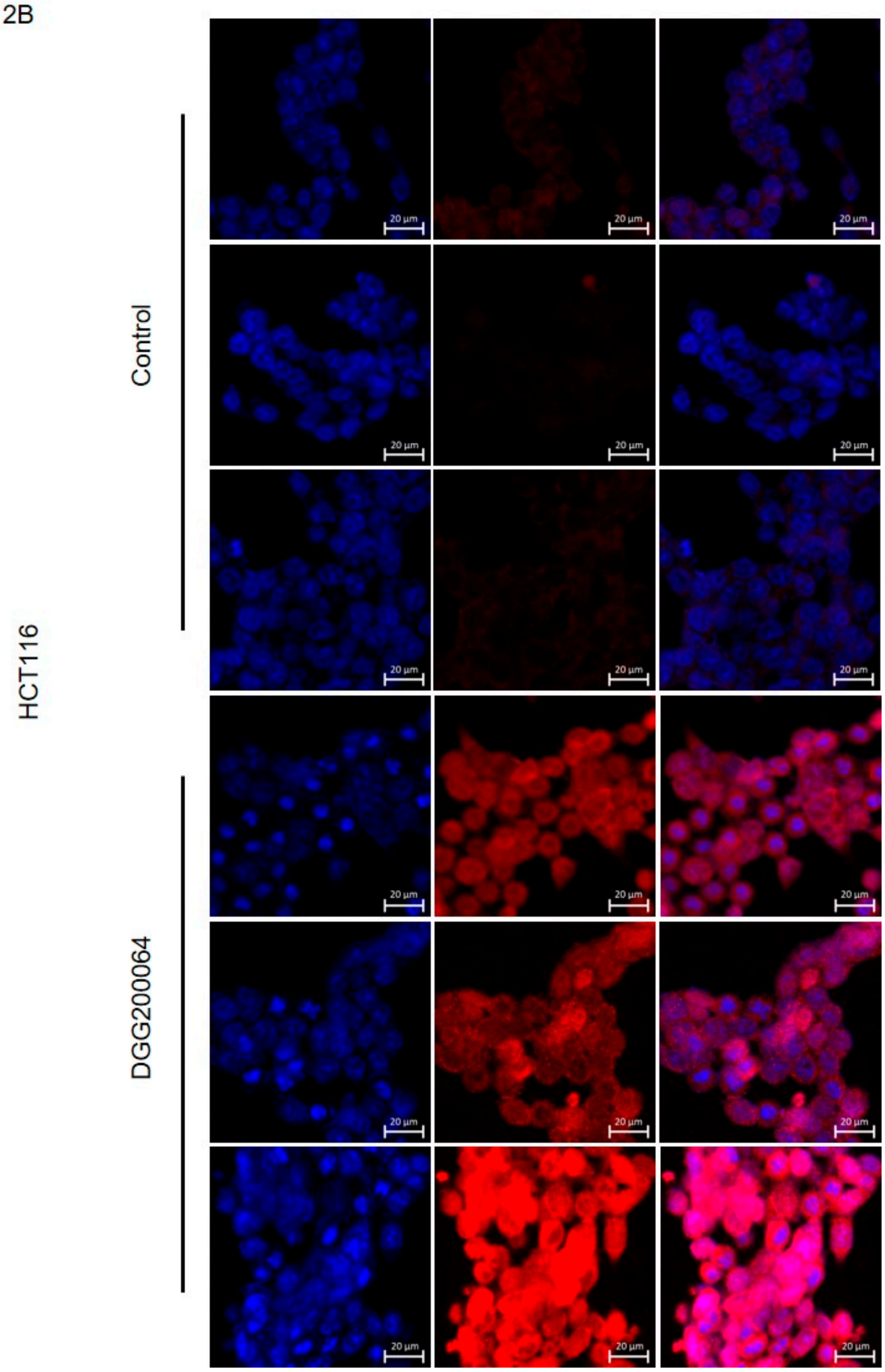

Figure 2C

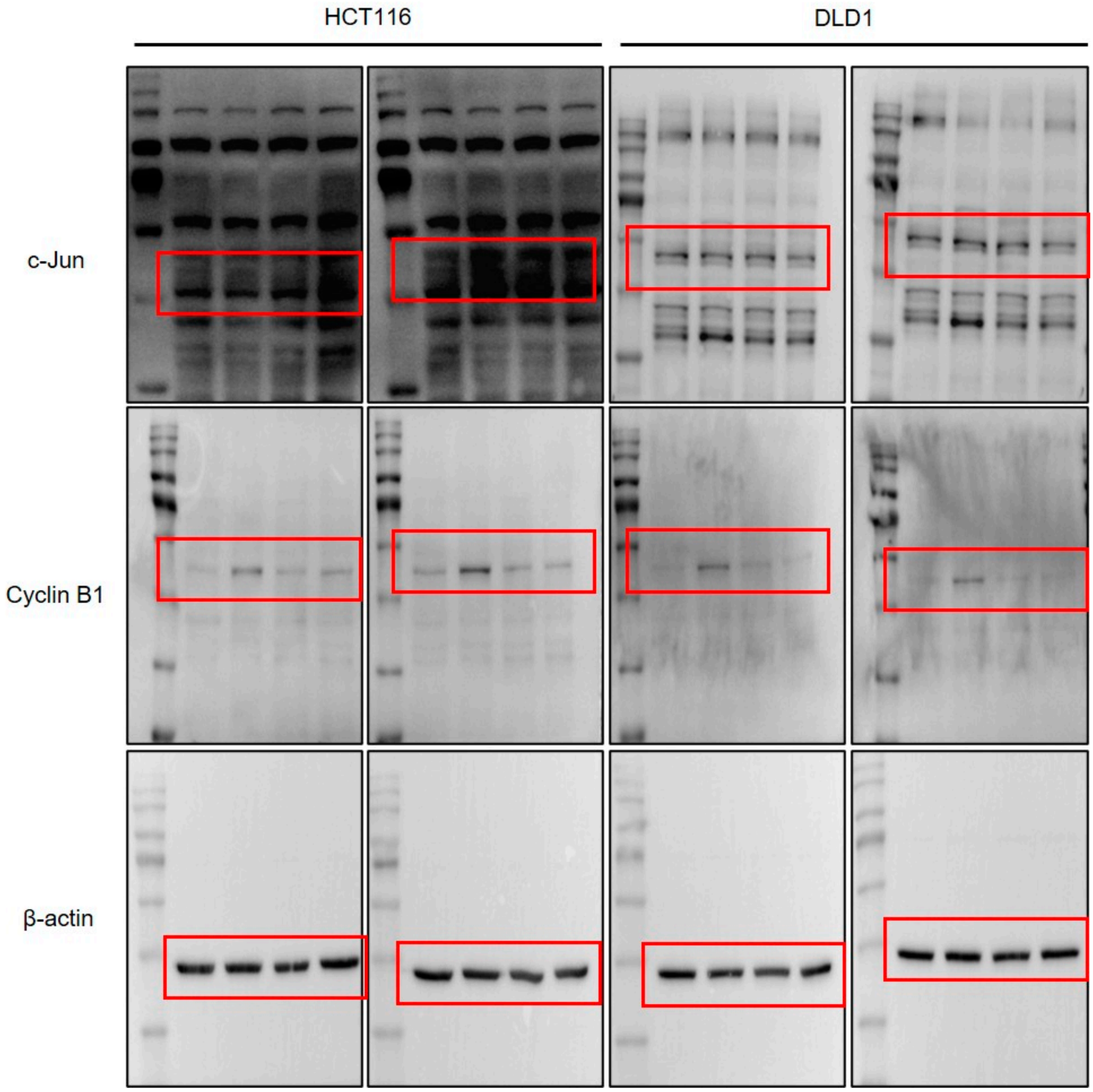

Figure 2D

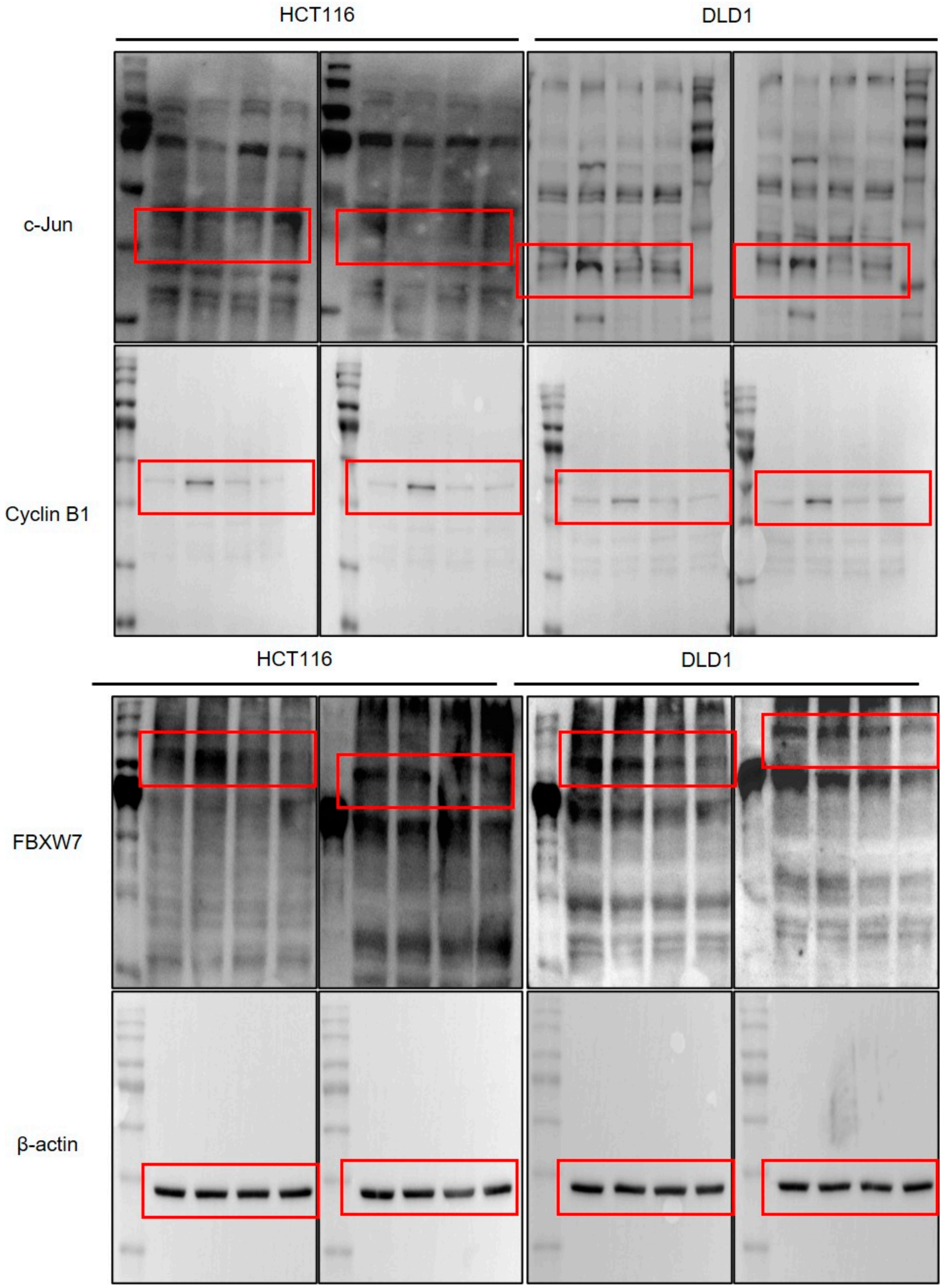

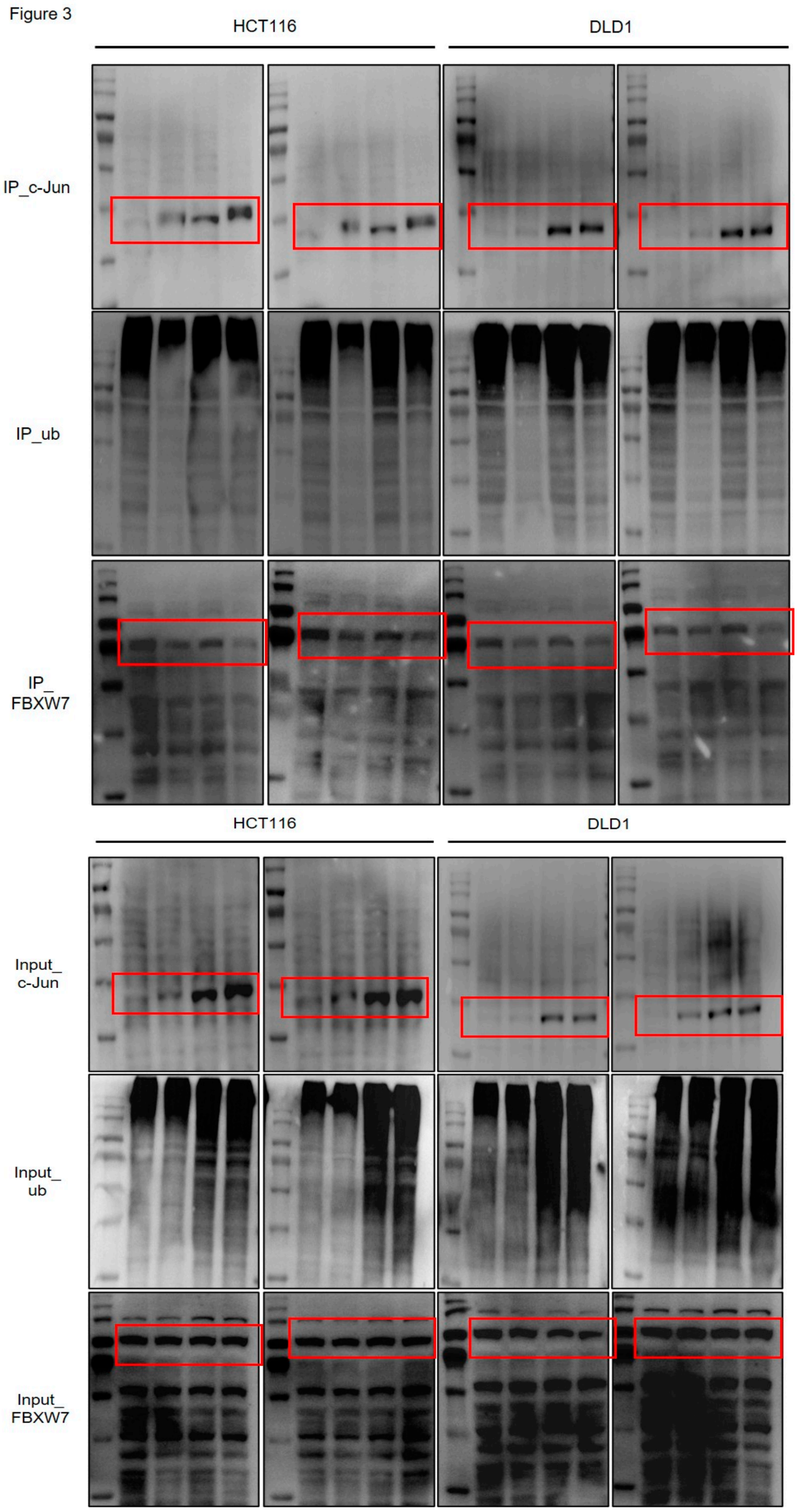
Figure 3
HCT116
DLD1
IP_c-Jun
IP_ub
IP_
FBXW7
HCT116
DLD1
Input_
c-Jun
Input_
ub
Input_
FBXW7

Figure 4

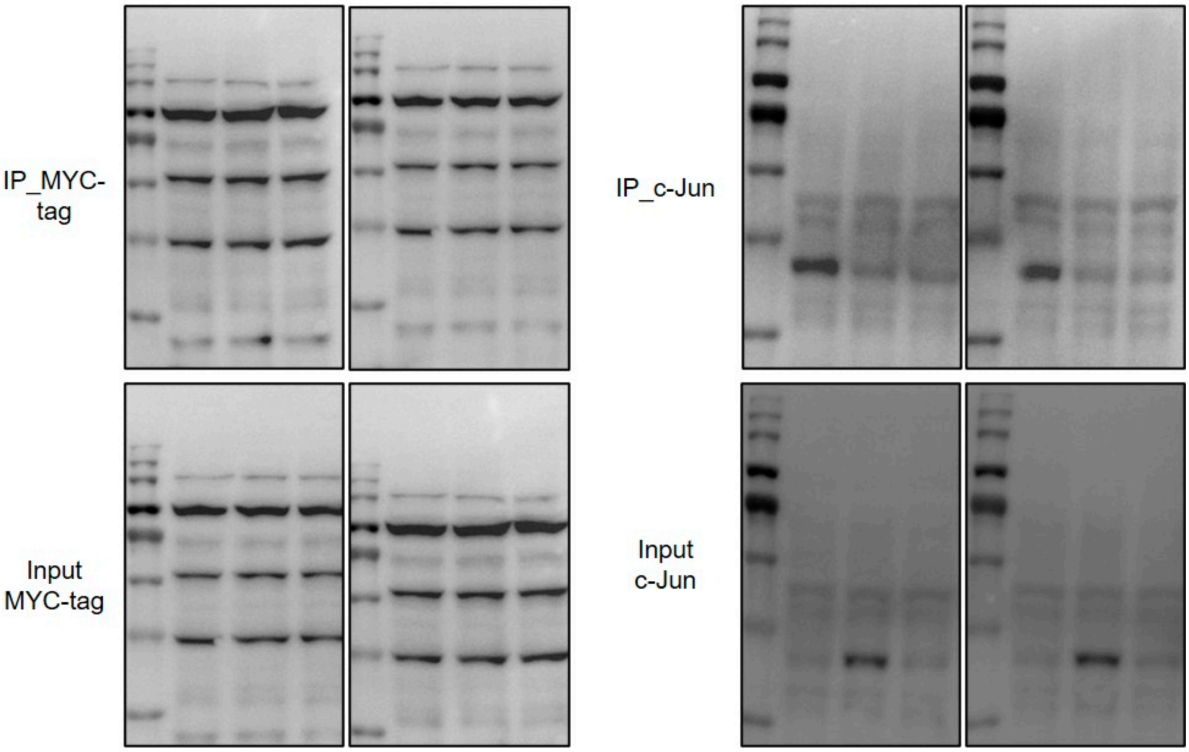

Figure S1 DGG200064 treatment on Colon cancer cells

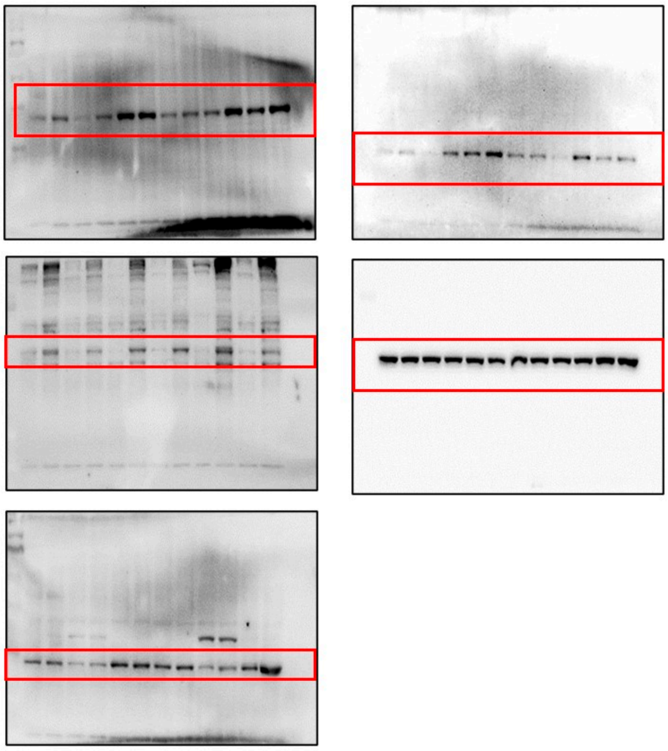

Figure S2 (a) DGG200064 treatment

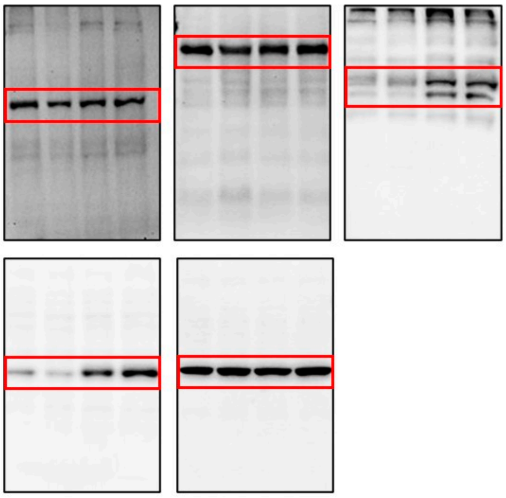

Figure S2 (b) DGG200064 treatment IP with Cyclin E

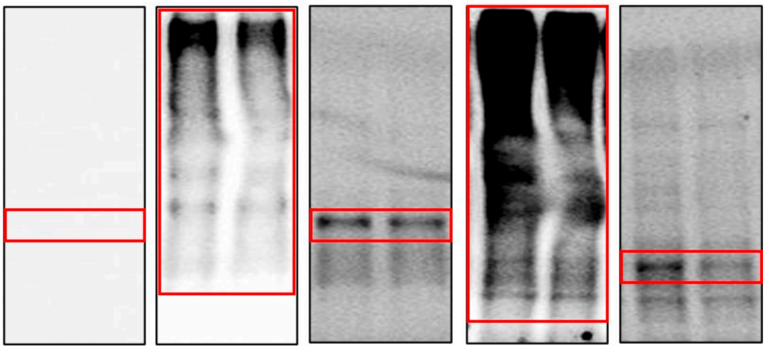

Table S1. ***In vivo*** **evaluation of the DGG200066 suppressed colon cancer tumor growth against HCT1116 cells.**

Tumor size (mm3)								
		0	5	8	11	14	18	21
평균	Control	212.05	651.8625	794.9103	941.4363	1074.954	1179.893	1203.246
	DGG200064 60 mg/kg	188.7437	298.6004	319.7996	426.3946	508.0443	536.6033	606.2556
	DGG200064 120 mg/kg	171.0132	246.6181	272.2828	305.558	316.6981	327.5194	374.4137
		0	5	8	11	14	18	21
편차	Control	116.7022	217.3067	386.7541	407.5402	339.1767	305.9519	236.0849
	DGG200064 60 mg/kg	92.02225	197.2219	196.8343	225.5302	212.5568	279.4415	287.8924
	DGG200064 120 mg/kg	66.22986	151.8436	156.099	182.5079	142.3078	143.5347	99.41429
Bodyweight (g)								
		0	5	8	11	14	18	21
평균	Control	18.16667	19	20.16667	19.16667	19.33333	20.16667	20.5
	DGG200064 60 mg/kg	18.66667	18.83333	19	19.16667	19.16667	19.66667	20.33333
	DGG200064 120 mg/kg	18.33333	18.33333	18.83333	18.66667	17.83333	18.83333	19
		0	5	8	11	14	18	21
편차	Control	0.897527	1	1.213352	0.897527	0.745356	1.213352	0.957427
	DGG200064 60 mg/kg	0.745356	0.897527	1.290994	1.213352	1.213352	1.105542	1.247219
	DGG200064 120 mg/kg	0.471405	0.471405	0.897527	0.942809	1.57233	1.34371	1.632993

Tumor size (mm^3)											
	DGG200338	1	4	7	10	13	16	19	22	25	28
평균	0 mg/kg	82.66304	151.2889	246.1779	325.6998	463.7817	574.7773	782.8934	893.7176	983.6118	1080.272
	35 mg/kg	80.14855	115.5963	161.3056	173.9752	192.7666	208.7519	227.6788	271.1424	340.3376	339.7049
	70 mg/kg	81.25178	93.0595	115.7923	144.3401	165.0344	180.452	190.4336	220.6202	245.5077	243.8313
	DGG200338										
편차	0 mg/kg	48.98778	78.38627	114.5712	116.5257	196.5681	178.5368	301.0909	358.0715	415.5241	402.2574
	35 mg/kg	56.85341	66.18676	80.66902	95.0728	101.3432	102.1129	90.66668	110.2231	94.23366	117.5697
	70 mg/kg	43.2349	53.58528	76.1347	73.33556	99.90373	95.57271	68.48331	65.1613	92.32454	97.97786
body weight (g)											
	DGG200338	1	4	7	10	13	16	19	22	25	28
평균	0 mg/kg	19.14286	19.42857	19.85714	19.57143	20.14286	19.57143	20	19.71429	20	19.71429
	35 mg/kg	19.42857	19.71429	19.85714	19.57143	20.71429	20.28571	21	21.42857	22.71429	22
	70 mg/kg	18.85714	18.71429	18.28571	18	19	19.85714	19.28571	19.14286	20.57143	20
	DGG200338	1	4	7	10	13	16	19	22	25	28
편차	0 mg/kg	1.46385	1.511858	1.345185	1.272418	1.772811	1.511858	1.825742	2.21467	2.645751	2.429972
	35 mg/kg	1.272418	1.496026	1.46385	1.618347	1.380131	1.704336	1.825742	2.225395	2.13809	1.914854
	70 mg/kg	1.46385	1.496026	1.799471	1	1.732051	1.573592	1.496026	1.214986	1.511858	1.632993
